# Valorization of Mushroom By‐Products From *Agaricus bisporus* and *Pleurotus ostreatus* as Novel Feed Ingredients for Rainbow Trout (*Oncorhynchus mykiss*) Diets

**DOI:** 10.1155/anu/6294426

**Published:** 2026-08-03

**Authors:** Carl John Saromines, Enric Gisbert, Samira Reinoso, Maria Luisa Tello Martín, Margarita Pérez Clavijo, Silvia Torrecillas

**Affiliations:** ^1^ Institute of Agrifood Research and Technology (IRTA), Aquaculture, Centre de La Ràpita, 43540, La Ràpita, Catalonia, Spain; ^2^ Mushroom Technological Research Center of La Rioja (CTICH), Autol 26650, La Rioja, Spain

**Keywords:** alternative ingredient, digestibility, soybean meal replacement, sustainable aquafeed

## Abstract

A 60‐day feeding trial evaluated meals derived from discarded mushroom biomass, including stems and nonmarketable mushrooms, generated during commercial cultivation and processing of *Agaricus bisporus* (AB) and *Pleurotus ostreatus* (PO) as lower‐impact alternatives to soybean meal (SBM) in rainbow trout (*Oncorhynchus mykiss*, BWi: 22.1 ± 0.3 g) diets. Five isoproteic (47%) and isolipidic (23%) diets were tested: a control diet and four diets replacing 25%–50% of SBM with AB or PO meals (AB25, AB50, PO25, PO50). Fish fed the AB25 diet showed growth performance, feed intake (FI), and nutrient utilization comparable to the control (*p* > 0.05), whereas AB50 reduced growth (*p* < 0.05) without affecting FI or nutrient utilization (*p* > 0.05). In contrast, PO inclusion caused a progressive decline in these parameters, more pronounced at PO50, accompanied by increased hepatosomatic index (HSI) and reduced perivisceral fat index (PFI) (*p* < 0.05). Apparent protein digestibility decreased in the AB50 diet (*p* < 0.05). In PO‐fed fish, digestive enzyme activities reflected adaptive responses to reduced FI, including lower pepsin (PEP) and higher total alkaline proteases (TAPs), trypsin (TRY), lipase (LIP), aminopeptidase‐N (APN), and maltase (MAL) activities. Hematological and plasma markers remained within normal ranges, and liver histological organization was similar among treatments (*p* > 0.05). Hepatic glutathione reductase (GR) activity increased in PO diets (*p* < 0.05), whereas AB diets remained comparable to the control group (*p* > 0.05). Overall, mushroom by‐product meals were physiologically safe for *O. mykiss*, while AB meal emerged as a promising circular alternative to SBM, supporting up to 25% replacement without compromising growth performance, feed efficiency, or nutrient utilization.

## 1. Introduction

The global expansion of aquaculture places increasing pressure on the production and sustainability of aquafeeds [1, 2]. This challenge involves not only producing higher aquafeed volumes but also developing nutritionally adequate, economically viable, and environmentally sustainable feed formulations [3]. Over recent decades, aquafeed formulations have progressively shifted from predominantly marine‐based ingredients toward more diversified compositions [2]. Modern commercial aquafeed formulations increasingly rely on plant‐derived ingredients, among which soybean meal (SBM) has become a major substitute for traditional marine ingredients such as fishmeal (FM) [2, 4, 5]. Advances in processing technologies including heat treatment, fermentation, enzyme supplementation, and protein concentration have improved its nutritional quality and reduced the content of antinutritional factors (ANFs) [6, 7]. Nevertheless, the continued reliance on SBM raises environmental and socioeconomic concerns, including deforestation, greenhouse gas emissions, and vulnerability to supply chains. For example, the EU relies on soybean imports for ~70% of its protein feed supply, leading to competition with food and livestock production and increased price volatility [4, 8–10]. These challenges reinforce the need to diversify aquafeed formulations by incorporating novel ingredients with reduced environmental impact and improved compatibility with circular economy approaches [3].

In this context, agrifood by‐product‐derived raw materials are a promising strategy to enhance the sustainability of aquafeed formulations [11]. In particular, the mushroom industry generates substantial quantities of by‐products, often discarded through composting, landfilling, or incineration [12]. At the global scale, the mushroom industry generates substantial quantities of residual biomass, with an estimated 60 Mt of mushroom by‐products produced annually, representing up to 50% of total mushroom biomass generated worldwide [12–14], while in Europe, mushroom production (*ca*. 1.3 million ton in 2024) generates *ca*. 150 kg of by‐products per ton of mushrooms was produced [15]. Owing to their high availability, low economic value, and noncompetition with human food systems, mushroom production by‐products represent a potentially sustainable alternative ingredient for aquafeed formulations.

Evaluating alternative aquafeed ingredients involves several key criteria, such as evaluation of digestibility, nutrient and energy utilization, and assessments of physiological parameters through nutritional trials [16]. Within this framework, recent studies have highlighted the high apparent digestibility of mushroom by‐products meals from three commercially cultivated edible species, *Agaricus bisporus* (AB) (white button mushroom), *Lentinula edodes* (Shiitake mushroom), and *Pleurotus ostreatus* (PO) (oyster mushroom) in rainbow trout (*Oncorhynchus mykiss*), with protein digestibility values ranging from 77% and up to 90% when included in commercial‐like diets [17]. Indeed, at an inclusion level of 300 g kg^−1^ feed (30%), digestibility trials showed no negative effects on growth performance or feed efficiency once differences in dietary crude protein (CP) and lipid content were considered, whereas such high dietary inclusion levels induced region‐specific changes in the gut microbiota [18]. In addition, several studies have reported unaltered or enhanced nutrient utilization, health, and immune parameters in fish fed diets including mushroom biomass and extracts obtained from different mushroom species [19–21]. However, most of these studies have focused on their use as feed additives due to their content of bioactive compounds, such as β‐glucans or bioactive compounds with direct antioxidant properties [19, 20]. Despite these promising results, the evaluation of mushrooms as alternative raw materials remains limited, and their evaluation has been predominantly focused on fish species with herbivorous or omnivorous feeding habits. For instance, in *Oreochromis niloticus*, *Pleurotus sajor-caju* and *Schizophyllum commune* by‐products have been successfully used to replace up to 60% of FM; however, higher inclusion levels resulted in reduced growth, likely linked to increased dietary fiber and decreased protein digestibility [22]. Complete replacement of rice bran with *P. florida* stalks in *O. niloticus* has also been achieved without adverse effects, probably because of their fermentable carbohydrate content and acceptable nutrient profile [23]. Additionally, *P. djamor* meal replaced up to 25% of SBM, improving growth and feed efficiency, in relation to enhanced gut enzyme activity and the presence of bioactive compounds, including polysaccharides such as β‐glucans, polyphenols (e.g., p‐coumaric acid, gallic acid, and ferulic acid), and terpenes and terpenoids [24, 25].

Under this scenario, we hypothesized that mushroom industry by‐products from AB and PO could effectively replace SBM in commercial‐type rainbow trout diets without compromising growth performance or feed efficiency based on their previously reported protein digestibility and demonstrated efficacy in aquafeeds [17, 22, 23, 25]. To advance understanding of sustainable aquafeed alternative ingredients, this study evaluated edible mushroom by‐products from industrially produced AB and PO as potential replacements for SBM in rainbow trout diets. The objectives were to determine the optimal mushroom species and SBM replacement level based on the effects on growth performance, feed and nutrient utilization efficiency, and key physiological and biochemical markers of fish growth and welfare.

## 2. Materials and Methods

### 2.1. Experimental Diets

Five isoproteic and isolipidic experimental diets (CP: *ca*. 47%, crude lipid, CL: *ca*. 23%) were formulated with graded levels of SBM replacement (0%, 25%, and 50%) by AB and PO meals, corresponding to a nominal dietary inclusion of 0, 50 g kg^−1^ and 100 g kg^−1^ diet. Diets were named as CTRL (control diet), AB25, AB50, PO25, and PO50 according to the increasing levels of SBM replacement by AB and PO meals. Mushroom stems were obtained from a commercial farm in La Rioja, Spain, and processed at the Mushroom Technological Research Center of La Rioja (CTICH, Autol, Spain). Mushroom processing consisted of cutting, drying at 35°C, and milling into powder, which was subsequently analyzed for nutrient composition and incorporated into experimental diets. The proximate composition of the mushroom meals and formulation and chemical composition of experimental diets are presented in Tables 1 and 2, respectively [17]. Experimental diets (2.5 mm pellets) were produced by Sparos Lda. (Portugal) following the procedure described by Ruiz et al. [26]. In brief, dry ingredients and oils were mixed, pulverized (<200 µm), extruded using a twin‐screw extruder (Clextral BC45, Firminy, France) under standard conditions, and dried in a vibrating fluid bed dryer (TGC Extrusion DR100, Saint‐Étienne, France).

**Table 1:** Proximate composition, amino acid profile, and glucan content on a dry matter basis (DM) of mushroom stem meals from *Agaricus bisporus* and *Pleurotus ostreatus*.

Proximate composition (% DM)	Mushroom stem meals	
	*Agaricus bisporus*	*Pleurotus ostreatus*
Dry matter	91.5	89.4
Crude protein	21.1	10.8
Crude lipids	3.8	3.0
SFA	1.0	0.5
MUFA	0.2	0.3
* n −* 6 PUFA	2.5	2.2
* n* − 3 PUFA	0.0	0.0
Ash	17.1	9.0
Gross energy (cal/g)	3376	3525
Phosphorus (mg/g)	6.2	3.3
Calcium (mg/g)	34.7	0.7
Amino acids (% DM)		
Aspartic acid	1.19	0.66
Threonine	0.59	0.34
Serine	0.55	0.34
Glutamic acid	2.28	1.54
Glycine	0.64	0.36
Alanine	1.55	0.69
Valine	0.58	0.31
Methionine	1.02	0.39
Isoleucine	0.45	0.24
Leucine	0.81	0.47
Tyrosine	0.38	0.22
Phenylalanine	0.60	0.32
Lysine	1.25	0.64
Histidine	0.35	0.20
Tryptophan	0.07	0.03
Cysteine	0.17	0.07
Arginine	3.65	1.10
Proline	0.80	0.27
Glucans composition^a^ (% DM)		
Total glucans	11.67	37.46
Alpha‐glucans	5.96	7.40
Beta‐glucans	5.72	30.06

^**a**^The glucan contents of mushroom stem meals were analyzed using a β‐glucan assay kit (K‐YBGL, Megazyme, Michigan, USA) according to the manufacturer’s protocol.

**Table 2:** Formulation and proximate composition of the experimental diets replacing soybean meal with graded levels of *A. bisporus* (AB) and *P. ostreatus* (PO) meals for rainbow trout (*Oncorhynchus mykiss*).

Ingredients (%)	Experimental diets				
	CTRL	AB25	AB50	PO25	PO50
Fishmeal super prime^a^	10.00	10.00	10.00	10.00	10.00
Poultry meal^b^	8.00	8.00	8.00	8.00	8.00
Blood meal^c^	2.00	2.00	2.00	2.00	2.00
Soybean meal 48^d^	12.00	9.00	6.00	9.00	6.00
*Agaricus bisporus* meal (AB)^e^	—	5.00	10.00	—	—
*Pleurotus ostreatus* meal (PO)^f^	—	—	—	5.00	10.00
Soy protein concentrate^g^	14.00	14.00	14.00	14.00	14.00
Pea protein concentrate^h^	3.10	3.10	3.10	3.10	3.10
Corn gluten meal^i^	5.00	5.00	5.00	5.00	5.00
Wheat meal^j^	6.00	6.00	6.00	6.00	6.00
Wheat gluten^k^	10.00	10.60	11.30	10.70	11.60
Wheat starch (raw)^l^	7.00	4.50	1.90	4.40	1.60
Fish oil^m^	6.60	6.60	6.60	6.60	6.60
Rapeseed oil^n^	13.80	13.70	13.60	13.70	13.60
Vitamin and mineral premix^o^	1.00	1.00	1.00	1.00	1.00
Antioxidant^p^	0.18	0.18	0.18	0.18	0.18
Monoammonium phosphate^q^	1.00	1.00	1.00	1.00	1.00
L‐Lysine 99%^r^	0.20	0.20	0.20	0.20	0.20
DL‐methionine^s^	0.10	0.10	0.10	0.10	0.10
Yttrium oxide^t^	0.02	0.02	0.02	0.02	0.02
Proximate composition					
Crude protein	47.71	47.10	47.18	47.50	46.90
Crude fat	24.03	23.29	23.51	24.89	23.28
Crude fiber	1.01	1.30	1.46	1.51	1.82
Ash	4.98	5.92	6.23	5.32	5.71
Gross energy (MJ/kg feed)	23.40	23.38	23.52	23.32	23.76

^a^Fishmeal super prime: Pesquera Diamante, Peru; CP, 66.3%; CL, 11.5%.

^b^Poultry meal: SAVINOR UTS, Portugal; CP, 62.4%; CL, 12.5%.

^c^Blood meal: SONAC BV, The Netherlands; CP, 89.1%; CL, 0.4%.

^d^Soybean meal 48 (dehulled, solvent extracted): CARGILL, Spain; CP, 47.4%; CL, 2.6%.

^e^ *Agaricus bisporus* (AB) stem meal.

^f^ *Pleurotus ostreatus* (PO) stem meal.

^g^Soy protein concentrate: ADM, The Netherlands; CP, 62.2%; CL, 0.7%.

^h^Pea protein concentrate: Roquette, France; CP, 78.1%; CL, 8.3%.

^i^Corn gluten meal: COPAM, Portugal; CP, 61.2%; CF, 5.2%.

^j^Wheat meal: Molisur, Spain; CP, 11.7%; CL, 1.6%.

^k^Wheat gluten: Roquette, France; CP, 80.4%; CL, 5.8%.

^l^Wheat starch (90% starch): Tereos, France; CP, 0.4%; CL, 0.1%.

^m^Fish oil: Sopropêche, France; 16% EPA; 12% DHA.

^n^Rapeseed oil: JC Coimbra, Portugal; CL, 98.2%.

^o^Vitamin and mineral premix: Premix Lda, Portugal; vitamins—DL‐α‐tocopherol acetate 100 mg, sodium menadione bisulfate 25 mg, retinyl acetate 20,000 IU, DL‐cholecalciferol 2000 IU, thiamin 30 mg, riboflavin 30 mg, pyridoxine 20 mg, cyanocobalamin 0.1 mg, nicotinamide 200 mg, folic acid 15 mg, ascorbic acid 1000 mg, inositol 500 mg, biotin 3 mg, calcium pantothenate 100 mg, betaine 500 mg; minerals—cobalt carbonate 0.65 mg, copper sulfate 9 mg, ferric sulfate 6 mg, potassium iodide 0.5 mg, manganese oxide 9.6 mg, sodium selenite 0.01 mg, zinc sulfate 7.5 mg, sodium chloride 400 mg, calcium carbonate 1.86 g; excipient: wheat middlings.

^p^Antioxidant: Kemin Europe NV, Belgium.

^q^MAP (monoammonium phosphate): Phosphea, Serbia; P, 26%.

^r^L‐Lysine: Dongxiao Biotechnology, China; Lys, 99%.

^s^DL‐methionine: ADISSEO, France; Met, 99%.

^t^Yttrium oxide: Höganäs Germany GmbH, Germany.

### 2.2. Fish and Experimental Design

Juvenile rainbow trout were obtained from Truchas de Leiza SL (Leiza, Navarra, Spain) and transported to the research facilities of the IRTA (La Ràpita, Tarragona, Spain). Fish were acclimated for 2 weeks in a 10 m^3^ tank integrated into a single recirculating aquaculture system (IRTAmar) and fed the CTRL diet at a daily rate of 3% of the stocked biomass. During the experimental period, all tanks were connected to the same IRTAmar recirculating aquaculture system, which included mechanical and biological filtration units and a UV sterilization system. The tank was considered the experimental unit for statistical analyses because dietary treatments were applied at the tank level, whereas all tanks were maintained under identical environmental conditions to minimize system‐related variability among treatments. Following acclimation, all individuals (*N* = 800) were weighed and measured (body weight, BW: 22.1 ± 0.3 g; standard length, SL: 13.2 ± 0.1 cm; mean ± standard deviation [SD]) and randomly distributed into twenty 0.5 m^3^ tanks (0.44 kg m^−3^). Experimental diets were fed to rainbow trout for 60 days at an initial feeding rate of 2%–3% of the tank biomass, which was periodically adjusted by maintaining an uneaten percentage of 10%–15%, ensuring that fish were fed *ad libitum*. Fish were fed twice daily, at 08:00 and 13:00, using automatic feeders (ARVO‐TEC T Drum 2000; Arvotec, Huutokoski, Finland). Two hours after each meal, the uneaten pellets were recovered and dried overnight to determine the quantity of feed ingested daily. Water quality parameters were monitored and maintained within optimal ranges, and their values were as follows: temperature: 16.02 ± 0.19°C; dissolved oxygen: 7.20 ± 0.18 mg L^−1^ (OXI330, Crison Instruments, Alella, Spain); salinity: 1.65 ± 0.05 ppt; pH: 7.93 ± 0.12 (pH meter 507, Crison Instruments); ammonium: 0.49 ± 0.73 ppm, nitrite: 0.30 ± 0.14 ppm, and nitrate: 51.43 ± 14.60 ppm (DR 900 Colorimeter, Hach, Loveland, CO, USA). Water hardness (2515 µS cm^−1^) reflected the natural characteristics of the source water supplying the research facility. The photoperiod followed natural seasonal variations corresponding to the experimental period (October–December; 40.63°N, 0.66°E). Changes in water quality parameters during the experiment are presented in Figure S1.

### 2.3. Fish Sampling, Growth, and Key Performance Indicators (KPIs) Analyses

Fish biometry was performed after 30 and 60 days of feeding the experimental diets. Before sampling, fish were fasted overnight and anesthetized using MS‐222 (100 mg L^−1^). Individual fish BW (g) and SL (cm) were then recorded. The KPIs related to somatic growth and feed and nutrient utilization were calculated as follows: specific growth rate (SGR, % BW day^−1^) = ([ln BWf − ln BWi] x100)/(time [days]), total feed intake (FI, g) = (total feed given [g] – uneaten feed [g]), feed conversion ratio (FCR) = (total feed given [g] – uneaten feed [g])/(live weight gain [g]), protein efficiency ratio (PER) = (weight gain)/(protein intake [g dry weight basis]), lipid efficiency ratio (LER) = (weight gain)/(lipid intake [g dry weight basis]), survival (%) = (100 × final number of fish)/(initial number of fish), Fulton’s condition factor (K) = (BWf)/SLf^3^ x 100, viscerosomatic index (VSI) = (wet viscera weight)/(BWf) x 100, hepatosomatic index (HSI) = (wet liver weight)/(BWf) x100, perivisceral fat index (PFI) = (wet perivisceral fat weight)/(BWf) x 100, CP retention (%) = ([BWf × CPf] − [BWi × CPi])/(FI × Feed CP) × 100, CL retention (%) = ([BWf × CLf] − [BWi × CLi])/(FI × Feed CL) × 100; where CPf, CPi, CLi, and CLf are the protein and lipid content of fish at the beginning (i) and final (f) of the trial, and CL and CP are the crude lipid and crude protein feed contents, respectively.

At the end of the 60‐day trial, following fish biometry, 15 fish per tank were selected and euthanized with an overdose of MS‐222 (350 mg L^−1^) for tissue sampling (i.e., liver histology and analysis of digestive and oxidative stress enzymes). Specimens chosen for blood collection were first gently anesthetized with MS‐222 (100 mg L^−1^) prior to sampling and were subsequently euthanized, as previously indicated. Fish samples were distributed to different groups for biological sample collection (*n* = 3 fish per tank, 12 per diet). Samples collected include blood for biochemical and hematological analyses, whole fish for proximate composition, stomach and intestine samples for digestive enzyme analyses, and liver for histological and oxidative stress analyses. Representative whole fish samples (*n* = 15) were also collected at day 0 for whole‐body proximate composition analysis, which were used for calculating nutrient retention values.

### 2.4. Diet Apparent Digestibility Analysis

Fecal sampling was conducted at 30 days after the start of feeding the experimental diets, following the recommendations of Glencross et al. [27]. The procedures for the collection of excreted feces were previously detailed in the study by Saromines et al. [17]. Briefly, tanks and fecal settling columns were cleaned after feeding to remove uneaten pellets. Feces that settled overnight were collected the next morning and stored at −20°C until sufficient material was obtained for analysis. Dry matter (DM), CP, CL, and gross energy (GE) apparent digestibility coefficients (ADCs) were calculated as follows [28]: ADC DM (%) = 100 − (100 × [marker diet/marker feces]); ADC CP, CL, or GE (%) = 100 − (100 × [(nutrient diet/nutrient feces) x (marker feces/marker diet)]); where marker diet and marker feces are the content of yttrium oxide (Y_2_O_3_) in the diet and feces, respectively; and nutrient diet and nutrient feces are the content of the targeted nutrient in diets and feces, respectively.

### 2.5. Chemical Analysis

The proximate composition of the experimental diets, fecal samples, and whole fish, as well as the marker concentrations in diets and feces, was determined based on AOAC [29] methods. DM was measured by drying samples at 105°C for 14 h (AOAC 925.09), while ash content was obtained through incineration in a muffle furnace (Nabertherm, Germany) at 500°C for 5 h (AOAC 942.05). CP was quantified using the Dumas combustion method with a nitrogen analyzer (FP‐528, Leco, USA; AOAC 968.06), and CL was determined following Folch’s procedure [30]. The content of GE was analyzed using an adiabatic bomb calorimeter in accordance with DIN 51900 standards, while Y_2_O_3_ concentrations were quantified using inductively coupled plasma optical emission spectroscopy (ICP‐OES; Optima 2100DV, PerkinElmer Life and Analytical Sciences, Shelton, USA; AOAC 984.27). Carbohydrates (CHO) were determined following the phenol–sulfuric acid assay [31]. Crude fiber (CF) was analyzed using an Ankom fiber analyzer (Ankom, USA) based on filter bag technology (AOAC 962.09).

### 2.6. Digestive Enzymes Analyses

At the end of the trial, fish were fasted for 24 h prior to the collection of their gastrointestinal tract for assessing the activity of gastric, pancreatic, and intestinal enzymes. This choice was done in order to guarantee that all fish were under the same basal digestive condition and minimize interindividual postprandial fluctuations in digestive enzyme activities due to different FI rates among dietary groups. This fasting period ensured that the measured enzyme activities reflected basal digestive capacity and enabled standardized comparisons among experimental treatments [32–34]. Digestive enzyme activities were analyzed in the stomach and intestine (including the pyloric ceca) from three fish per tank. The tissue from those specimens was pooled and processed according to standard spectrophotometric methods [35]. Sample handling and storage procedures followed recommended protocols to minimize enzymatic degradation [36].

Stomach samples were homogenized in Milli‐Q water (1:1 w/v), followed by sonication, and centrifuged. The resulting supernatant was collected and stored at −80°C for subsequent determination of pepsin (PEP) activity using hemoglobin as a substrate in a 1 N HCl buffer (pH 3.0) [34]. Intestinal samples, including pyloric ceca, were homogenized in a Tris‐mannitol buffer containing CaCl_2_ (pH 7.0) (1:30 w/v), sonicated, centrifuged, and the supernatants stored for pancreatic and brush‐border enzyme analyses. Pancreatic enzyme activities were determined as follows: α‐amylase (α‐AMY) activity was measured using soluble starch as the substrate in Na_2_HPO_4_ buffer (pH 7.4) [37]; trypsin (TRY) activity was determined using BAPNA in 50 mM Tris‐HCl and 20 mM CaCl_2_ buffer (pH 8.2) [38]; chymotrypsin (CHY) activity was measured using BTEE as the substrate in 80 mM Tris‐HCl and 100 mM CaCl_2_ buffer (pH 7.2) [39]; bile salt‐activated lipase (LIP) with p‐nitrophenyl myristate as the substrate in 0.25 mM Tris‐HCl, 0.25 mM 2‐methoxyethanol, and 5 mM sodium cholate buffer (pH 9.0) [40]; and total alkaline protease (TAP) activity with azo‐casein as the substrate in 50 mM Tris‐HCl buffer (pH 8.0) [41]. The activities of brush border membrane enzymes were determined as follows: alkaline phosphatase (ALPi) activity using p‐nitrophenyl phosphate as a substrate in 50 mM Na_2_CO_3_ buffer containing 40 mM MgCl_2_ (pH 9.8) [33], aminopeptidase‐N (APN) activity using L‐leucine p‐nitroanilide in 0.2 M phosphate buffer (pH 7.0) [42], and maltase (MAL) activity using D(+)‐maltose as a substrate in sodium maleate buffer (pH 6.0). Cytosolic leucine–alanine peptidase (LAP) was analyzed using L‐alanine as a substrate in 50 mM Tris‐HCl buffer (pH 8.0). All enzymatic determinations were performed in triplicate (methodological replications) using a multiplate spectrophotometer (Tecan Infinite M200 Plate Reader, Tecan, Männedorf, Switzerland) under controlled temperature conditions (20°C). Results were expressed as total enzymatic activity (U g tissue^−1^) consistent with internal analytical protocols and with previous published studies [33, 34, 43, 44].

### 2.7. Blood Biochemical and Hematological Analysis

Blood was collected by caudal puncture (*n* = 3 fish per tank) in gently anesthetized specimens. Blood samples were immediately transferred into tubes with lithium heparin or without anticoagulant for plasma and serum separation, respectively. Blood biochemical parameters, including albumin (ALB), total protein (TP), globulin (GLOB), plasma alkaline phosphatase (ALPp), amylase (AMY), plasma LIP (LIPp), creatine kinase (CK), lactate dehydrogenase (LDH), aspartate aminotransferase (AST‐SGOT), alanine aminotransferase (ALT/GPT), total bilirubin (TBIL), cholesterol (CHOL), triglycerides (TG), glucose (GLU), creatinine (CREA), calcium (Ca^2+^), phosphorus (PO_4_^3+^), magnesium (Mg^2+^), sodium (Na^+^), potassium (K^+^), and chloride (Cl^−^) were analyzed using a UV/Vis spectrophotometer (Vitros 5600 Integrated System & Analyzer, QuidelOrtho, San Diego, CA, USA). Hematological parameters, including erythrocyte (RBC) and leukocyte (WBC) counts, hematocrit (Ht), hemoglobin (Hb), lymphocytes (LYMs), monocytes (MONs), neutrophils (NEU), mean cell volume (MCV), mean cell hemoglobin (MCH), and MCH concentration (MCHC), were measured using a blood count chamber. Both biochemical and hematological analyses (*n* = 12 fish per diet) were conducted by Laboratorios Echevarne (veterinary division), Barcelona, Spain.

### 2.8. Hepatic Lipid Peroxidation (LPO), Total Antioxidant Capacity (TAC) and Activity of Antioxidant Stress Enzymes

Fish used for liver sampling were randomly selected and fasted overnight prior to the collection. Fish were euthanized by an overdose of MS‐222 (350 mg L^−1^), after which the liver was excised and collected. Three liver samples (~60 mg each) from each tank (*n* = 3 fish per tank, pooled, 4 tanks per diet) were pooled and homogenized in five volumes (w/v) of buffer containing 150 mM KCl and 1 mM EDTA (pH 7.4) using an IKA T25 digital ULTRA‐TURRAX (IKA Works). Pooling was performed at the tank level to ensure that biochemical analyses reflected the experimental unit and to avoid pseudo‐replication in statistical comparisons. The homogenates were centrifuged at 9000 × *g* for 30 min at 4°C, and the resulting supernatants were collected for analysis. Glutathione reductase (GR) activity was determined at λ = 340 nm using NADPH (0.09 mM) as a cofactor and oxidized glutathione (0.9 mM) as a substrate [45]. Catalase (CAT) activity was measured at λ = 240 nm using hydrogen peroxide (50 mM) as a substrate [46], while glutathione peroxidase (GPX) activity was assessed at λ = 340 nm with NADPH (0.292 mM), reduced glutathione (2.5 mM), and cumene hydroperoxide (0.625 mM) as substrates [47]. LPO was quantified using 1‐methyl‐2‐phenylindole (10.3 mM) in acetonitrile:methanol (1:3 v/v), with 1,1,3,3‐tetramethoxypropane as a malondialdehyde (MDA) standard [48]. The TAC was analyzed using the ferric antioxidant power (FRAP) colorimetric test (Invitrogen, Thermo Fisher Scientific, Spain), following the manufacturer’s protocol. All assays were performed in triplicate at 25°C in 96‐well microplates and measured using a UV/Vis multiplate spectrophotometer (Tecan Infinite M200 Plate Reader, Tecan, Männedorf, Switzerland) with data processed using Magellan software (v6, Tecan). Results obtained were normalized to the soluble protein content [49].

### 2.9. Liver Morphological Analysis

Liver samples (*n* = 3 fish per tank, analyzed individually, 4 tanks per diet) were dissected and fixed in 4% neutral‐buffered formalin. Following fixation, samples were embedded in paraffin and sectioned at 4 μm thickness using a Leica 2055‐Autocut microtome (Leica Instruments GmbH, Nussloch, Germany). Sections were stained with hematoxylin and eosin (H&E) and examined under a light microscope (Motic BA310E; Barcelona, Spain). Qualitative histomorphological evaluation included assessment of the hepatocytes size, degree of cytoplasmic vacuolization, alignment of nuclei along sinusoidal lines, blood congestion, and degree of fat deposits within hepatocytes. Morphological differences were scored using a semiquantitative scale ranging from 1 to 5 [26]. All evaluations were conducted independently by two observers, unaware of the experimental conditions. Digital images were taken at a final magnification of 400x using a Motic MOTICAMProS5 LiteCamera (Motic, Barcelona, Spain).

### 2.10. Statistical Analyses

All data are expressed as mean ± SD calculated from the four replicate tanks, with the tank considered as the experimental unit. Plasma and serum samples, however, were analyzed individually to account for the inherent interindividual variability associated with these sample types. In addition, the coefficient of variation (CV, %) was calculated for BW and K to assess the within‐treatment variability. Normality of residuals and homogeneity of variances were assessed using the Shapiro–Wilk and Levene’s tests, respectively. In addition, diagnostic evaluation of model assumptions included inspection of Q–Q plots of residuals, histograms of residual distributions, and residuals versus fitted value plots to assess homoscedasticity. The full diagnostic report, including statistical test outputs and graphical diagnostics, is provided in Table S1. Percentage data, as well as data that violated the ANOVA assumptions, were transformed when necessary to meet the model assumptions [50]. Data were analyzed using a general linear model including the five dietary treatments. A priori orthogonal contrasts were constructed to test the experimental hypotheses: (1) control *vs* all experimental diets, (2) meal source (AB vs. PO), (3) inclusion level (25% vs. 50% SMB replacement), and (4) meal source × inclusion level interaction. Contrasts were mutually orthogonal, allowing the partitioning of treatment variance into independent, hypothesis‐driven components within a single analytical framework. This hypothesis‐driven approach was preferred over post hoc testing because it allows direct testing of biologically meaningful, prespecified questions while controlling the type‐I error rate and partitioning treatment variance into independent components within a single analytical framework. When the contrast comparing the control diet with the experimental diets was significant, pairwise comparisons between the control and each experimental diet were performed using Dunnett’s test. Semiquantitative variables were analyzed using the Kruskal–Wallis test, followed by the Mann–Whitney *U* test. The significance level was set at *α* = 0.05. All analyses were performed using standard statistical procedures [51] with SPSS 21.0 software (IBM Corp., Armonk, NY, USA). Furthermore, to explore multivariate patterns among dietary treatments, a principal component analysis (PCA) was conducted. This approach considered all measured response variables in rainbow trout fed experimental diets, including fish performance, ADCs, whole‐body composition, nutrient retention, digestive enzyme activities, blood biochemical and hematological parameters, and oxidative stress and antioxidant responses. The percentage of variance explained by each principal component (PC) and the contribution of individual variables to component formation were used to interpret multivariate relationships among dietary treatments. The PCA was performed using RStudio (v4.5.1), and graphical visualization of individuals, variable contributions, confidence ellipses, and correlation circles was generated using the *factoextra* (v2.0) and *ggplot2* (v4.0.2) packages.

## 3. Results

### 3.1. PCA

**Figure 1:**
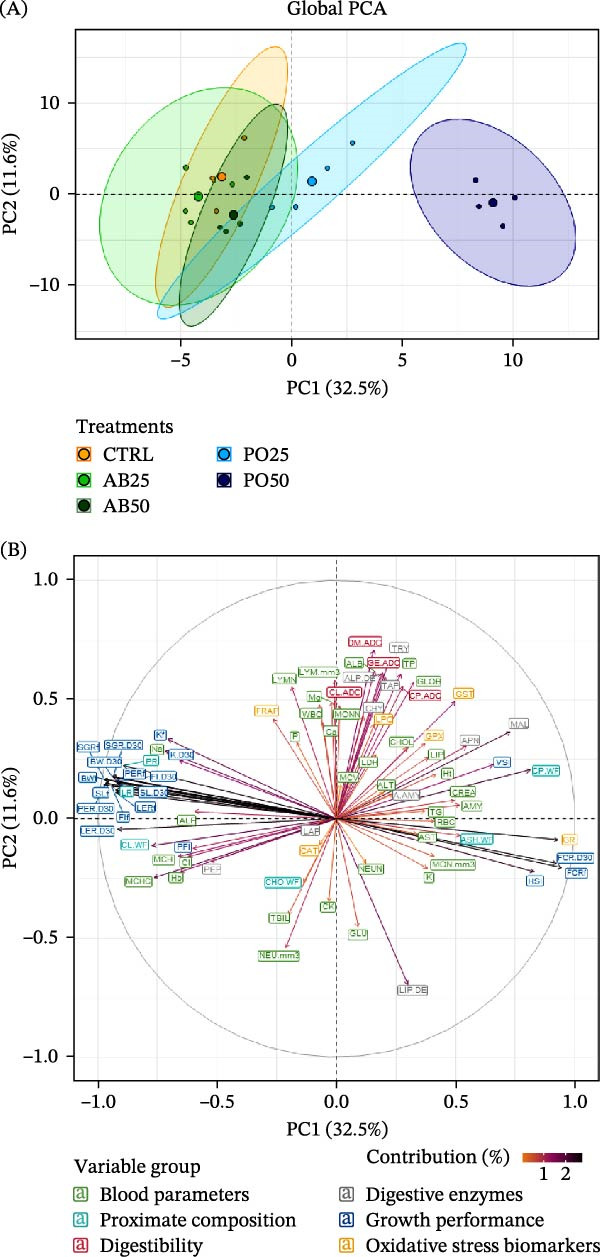
Principal component analysis (PCA) representing the variability in fish performance, diet apparent digestibility coefficients, whole body proximate composition, nutrient retention, digestive enzyme activities, blood biochemical and hematological parameters, lipid peroxidation and total antioxidant capacity, and activity of antioxidant enzymes of rainbow trout (*Oncorhynchus mykiss*) fed experimental diets replacing soybean meal with graded levels of *A. bisporus* (AB) and *P. ostreatus* (PO) meals for 60 days. The global PCA (A) illustrates the separation of variables among dietary treatments. Points represent replicate observations and are colored according to dietary treatment, with confidence ellipses drawn around group centroids (larger circle). The contribution plot (B) shows the contribution of each variable to the formation of PC1 and PC2. Variables with stronger correlations to the PC1 or PC2 have arrowheads positioned closer to the circumference of the correlation circle. Variables included in the PCA were as follows: growth performance and feed utilization (SURV.D30 and SURVf, survival at day 30 and day 60, respectively; BW.D30 and BWf, body weight at day 30 and day 60; SL.D30 and SLf, standard length at day 30 and day 60; K.D30 and Kf, condition factor at day 30 and day 60; SGR.D30 and SGRf, specific growth rate at day 30 and day 60; FCR.D30 and FCRf, feed conversion ratio at day 30 and day 60; FI.D30 and FIf, feed intake at day 30 and day 60; PER.D30 and PERf, protein efficiency ratio at day 30 and day 60; LER.D30 and LERf, lipid efficiency ratio at day 30 and day 60; VSI, viscerosomatic index; HSI, hepatosomatic index; PFI, perivisceral fat index); apparent digestibility coefficients (DM.ADC, dry matter apparent digestibility coefficient; CP.ADC, crude protein apparent digestibility coefficient; CL.ADC, crude lipid apparent digestibility coefficient; GE.ADC, gross energy apparent digestibility coefficient); whole‐body composition and nutrient retention (CP.WF, whole‐body crude protein; CL.WF, whole‐body crude lipid; ASH.WF, whole‐body ash; CHO.WF, whole‐body carbohydrates; PR, protein retention; LR, lipid retention); digestive enzyme activities (PEP, pepsin; α‐AMY, α‐amylase; CHY, chymotrypsin; LIP, bile‐salt activated lipase; TRY, trypsin; TAP, total alkaline protease activity; ALPi, intestinal alkaline phosphatase; APN, aminopeptidase‐N; MAL, maltase; LAP, leucine aminopeptidase); blood biochemical and hematological parameters (Ca^2+^, calcium; Cl^-^, chloride; Mg^2+^, magnesium; PO_4_^3+^, phosphorus; K^+^, potassium; Na^+^, sodium; ALP, alkaline phosphatase; ALT, alanine aminotransferase; AST, aspartate aminotransferase; AMY, amylase; CK, creatine kinase; LDH, lactate dehydrogenase; LIPp, plasma lipase; ALB, albumin; GLOB, globulin; TP, total protein; CHOL, cholesterol; CREA, creatinine; GLU, glucose; TBIL, total bilirubin; TG, triglycerides; RBC, red blood cell count; WBC, white blood cell count; Ht, hematocrit; Hb, hemoglobin; LYM.mm^3^, lymphocyte count (cells mm^−3^); LYMN, lymphocyte percentage; MON.mm^3^, monocyte count (cells mm^−3^); MONN, monocyte percentage; NEU.mm^3^, neutrophil count (cells mm^−3^); NEUN, neutrophil percentage; MCV, mean corpuscular volume; MCH, mean corpuscular hemoglobin; MCHC, mean corpuscular hemoglobin concentration); oxidative stress and antioxidant status (LPO, lipid peroxidation; FRAP, ferric reducing antioxidant power; CAT, catalase; GR, glutathione reductase; GPX, glutathione peroxidase; GST, glutathione S‐transferase).

To identify the variables contributing most to highlight the differences among dietary treatments, a PCA was performed using all measured zootechnical, physiological, biochemical, digestive, and nutrient utilization parameters (Figure 1). The first two PCs explained 44.1% of the total variance, with PC1 and PC2 accounting for 32.5% and 11.6%, respectively (Figure 1A). The PCA revealed a clear separation among dietary treatments, primarily along PC1. Fish fed the PO50 diet formed a distinct cluster on the right side of the axis, whereas CTRL, AB25, and AB50 diets clustered on the opposite side. In contrast, PO25 treatment occupied an intermediate position between the abovementioned dietary groups, indicating a transitional response pattern (Figure 1A).

Variables contributing most strongly to PC1 were mainly related to growth performance, feed utilization, and nutrient retention. In particular, the highest contributions were observed for BW, SGR, FCR, PER, and FI and retention coefficients for CP, CL, and GE (Figure 1B). These variables were positively associated with the separation of the PO50 group, indicating that differences in productive performance were the primary drivers of treatment discrimination. In contrast, PC2 was predominantly influenced by ADC values, particularly DM, GE, CP, and CL, together with digestive enzyme activities, including TRY, TAPs, bile salt‐activated LIP, and alkaline phosphatase. Furthermore, several blood and biochemical variables also contributed to this axis, although to a lesser extent compared to the former ones. The distinct positioning of fish fed the PO50 diet indicates markedly different physiological and productive responses compared with the other dietary treatments. This separation was driven principally by variation in growth performance and feed utilization variables (PC1), while digestibility and digestive enzyme activities contributed mainly to the secondary pattern of variation represented by PC2.

### 3.2. Fish Performance

**Figure 2:**
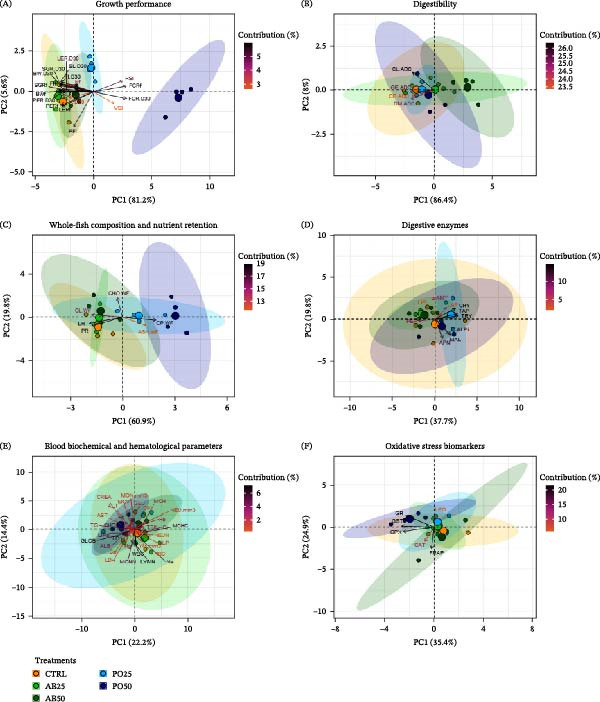
Principal component analyses (PCAs) of the main response categories evaluated in rainbow trout (*Oncorhynchus mykiss*) fed experimental diets replacing soybean meal with graded levels of *A. bisporus* (AB) and *P. ostreatus* (PO) meals for 60 days. Separate PCAs were performed for (A) growth performance and feed utilization, (B) apparent digestibility coefficients, (C) whole‐body composition and nutrient retention, (D) digestive enzyme activities, (E) blood biochemical and hematological parameters, and (F) lipid peroxidation and total antioxidant capacity, and activity of antioxidant enzymes. Points represent replicate observations and are colored according to dietary treatment (CTRL, AB25, AB50, PO25, and PO50), with confidence ellipses drawn around treatment centroids (larger points). Variable vectors indicate the direction and strength of the association of each variable with the principal components, whereas vector color represents the relative contribution (%) of each variable to the PCA ordination. Variables with stronger correlations to PC1 or PC2 have arrowheads positioned closer to the circumference of the correlation circle. Variables included in the PCA were as follows: growth performance and feed utilization (SURV.D30 and SURVf, survival at day 30 and day 60, respectively; BW.D30 and BWf, body weight at day 30 and day 60; SL.D30 and SLf, standard length at day 30 and day 60; K.D30 and Kf, condition factor at day 30 and day 60; SGR.D30 and SGRf, specific growth rate at day 30 and day 60; FCR.D30 and FCRf, feed conversion ratio at day 30 and day 60; FI.D30 and FIf, feed intake at day 30 and day 60; PER.D30 and PERf, protein efficiency ratio at day 30 and day 60; LER.D30 and LERf, lipid efficiency ratio at day 30 and day 60; VSI, viscerosomatic index; HSI, hepatosomatic index; PFI, perivisceral fat index); apparent digestibility coefficients (DM.ADC, dry matter apparent digestibility coefficient; CP.ADC, crude protein apparent digestibility coefficient; CL.ADC, crude lipid apparent digestibility coefficient; GE.ADC, gross energy apparent digestibility coefficient); whole‐body composition and nutrient retention (CP.WF, whole‐body crude protein; CL.WF, whole‐body crude lipid; ASH.WF, whole‐body ash; CHO.WF, whole‐body carbohydrates; PR, protein retention; LR, lipid retention); digestive enzyme activities (PEP, pepsin; α‐AMY, α‐amylase; CHY, chymotrypsin; LIP, bile‐salt‐activated lipase; TRY, trypsin; TAP, total alkaline protease activity; ALPi, intestinal alkaline phosphatase; APN, aminopeptidase‐N; MAL, maltase; LAP, leucine aminopeptidase); blood biochemical and hematological parameters (Ca²⁺, calcium; Cl⁻, chloride; Mg²⁺, magnesium; PO_4_^3+^, phosphorus; K⁺, potassium; Na⁺, sodium; ALP, alkaline phosphatase; ALT, alanine aminotransferase; AST, aspartate aminotransferase; AMY, amylase; CK, creatine kinase; LDH, lactate dehydrogenase; LIPp, plasma lipase; ALB, albumin; GLOB, globulin; TP, total protein; CHOL, cholesterol; CREA, creatinine; GLU, glucose; TBIL, total bilirubin; TG, triglycerides; RBC, red blood cell count; WBC, white blood cell count; Ht, hematocrit; Hb, hemoglobin; LYM.mm3, lymphocyte count (cells mm⁻³); LYMN, lymphocyte percentage; MON.mm^3^, monocyte count (cells mm⁻³); MONN, monocyte percentage; NEU.mm^3^, neutrophil count (cells mm⁻³); NEUN, neutrophil percentage; MCV, mean corpuscular volume; MCH, mean corpuscular hemoglobin; MCHC, mean corpuscular hemoglobin concentration); oxidative stress and antioxidant status (LPO, lipid peroxidation; FRAP, ferric reducing antioxidant power; CAT, catalase; GR, glutathione reductase; GPX, glutathione peroxidase; GST, glutathione S‐transferase).

According to the PCA, growth performance and feed utilization variables were among the major contributors to treatment discrimination, particularly driving the separation of fish fed the PO50 diet from the remaining groups (Figure 2A). Consistent with this pattern, growth performance, feed utilization, nutrient efficiency, and somatic indices were significantly affected by the source of mushroom meal, inclusion level, and their interaction. After 30 days of feeding, fish BW and SL were affected by mushroom meal source, inclusion level, and their interaction (*p* < 0.05, Table 3). Orthogonal polynomial contrasts indicated a quadratic decline in BW with increasing SBM replacement in both AB (*R* ^2^ = 0.75, *p* = 0.003) and PO diets (*R* ^2^ = 0.99, *p* < 0.001). Similarly, SL exhibited a quadratic decline in PO diets (*R* ^2^ = 0.97, *p* < 0.001), whereas no significant trend was detected in AB diets. Accordingly, fish fed the AB50 and PO50 diets showed lower BW than fish fed the CTRL diet (*p* < 0.05), whereas a reduction in SL was only observed in fish fed the PO50 diet (*p* < 0.05). The remaining experimental groups did not differ from the CTRL group (*p* > 0.05). Condition values were significantly affected by mushroom meal source (*p* < 0.05), with fish fed PO‐based diets exhibiting lower values than those fed the AB‐based diets, particularly in the PO50 treatment. However, neither inclusion level nor the interaction between factors significantly affected this parameter (*p* > 0.05), and no significant linear or quadratic response to increasing SBM replacement was detected in both AB and PO diets (*p* > 0.05).

**Table 3:** Initial biometric parameters and survival, growth performance, feed and nutrient efficiencies of rainbow trout (*Oncorhynchus mykiss*) at day 30 of feeding experimental diets replacing soybean meal with different inclusion levels (I) of mushroom stem meals (M) from *A. bisporus* (AB) and *P. ostreatus* (PO) meals.

Parameter	Experimental diets					Mushroom meal (M)	Inclusion level (I)	M x I	Polynomial orthogonal contrast^a^			
	CTRL	AB25	AB50	PO25	PO50							
									AB		PO	
									Linear	Quadratic	Linear	Quadratic
Initial												
BW (g)	22.10 ± 0.20	22.08 ± 0.31	22.12 ± 0.34	22.00 ± 0.21	22.06 ± 0.46	ns	ns	ns	—	—	—	—
CV_BW_ (%)	0.91	1.41	1.54	0.97	2.09	—	—	—	—	—	—	—
SL (cm)	13.19 ± 0.12	13.22 ± 0.07	13.19 ± 0.08	13.13 ± 0.11	13.10 ± 0.11	ns	ns	ns	—	—	—	—
K	0.96 ± 0.02	0.96 ± 0.03	0.96 ± 0.02	0.97 ± 0.02	0.98 ± 0.03	ns	ns	ns	—	—	—	—
CV_K_ (%)	2.25	2.67	2.06	2.16	2.94	—	—	—	—	—	—	—
Day 30												
Survival (%)	100 ± 0.00	100 ± 0.00	100 ± 0.00	100 ± 0.00	100 ± 0.00	ns	ns	ns	—	—	—	—
BW (g)	62.24 ± 0.89	63.33 ± 0.58	60.45 ± 0.89 ^∗^	61.34 ± 0.63	44.25 ± 1.27 ^∗^	<0.05	<0.05	<0.05	*R* ^2^ = 0.28, *p* = 0.01	*R* ^2^ = 0.75, *p* = 0.003	*R* ^2^ = 0.78, *p* < 0.001	*R* ^2^ = 0.99, *p* < 0.001
CV_BW_ (%)	1.42	0.91	1.47	1.03	2.87	—	—	—	—	—	—	—
SL (cm)	15.99 ± 0.18	16.05 ± 0.08	15.80 ± 0.08	15.94 ± 0.1	14.51 ± 0.12 ^∗^	<0.05	<0.05	<0.05	—	—	*R* ^2^ = 0.75, *p* < 0.001	*R* ^2^ = 0.97, *p* < 0.001
K	1.52 ± 0.05	1.53 ± 0.01	1.53 ± 0.04	1.51 ± 0.04	1.45 ± 0.05	<0.05	ns	ns	ns	ns	—	—
CV_K_ (%)	3.26	0.87	2.32	2.46	3.70	—	—	—	—	—	—	—
SGR (% day^−1^)	3.45 ± 0.03	3.51 ± 0.03	3.35 ± 0.02 ^∗^	3.42 ± 0.06	2.32 ± 0.06 ^∗^	<0.05	<0.05	<0.05	*R* ^2^ = 0.34, *p* < 0.001	*R* ^2^ = 0.88, *p* < 0.001	*R* ^2^ = 0.77, *p* < 0.001	*R* ^2^ = 0.99, *p* < 0.001
FCR	0.76 ± 0.01	0.75 ± 0.02	0.80 ± 0.01	0.77 ± 0.02	0.99 ± 0.04 ^∗^	<0.05	<0.05	<0.05	*R* ^2^ = 0.44, *p* = 0.001	*R* ^2^ = 0.76, *p* < 0.001	*R* ^2^ = 0.75, *p* < 0.001	*R* ^2^ = 0.95, *p* < 0.001
FI	1.22 ± 0.01	1.22 ± 0.01	1.22 ± 0.01	1.21 ± 0.01	0.86 ± 0.04 ^∗^	<0.05	<0.05	<0.05	—	—	*R* ^2^ = 0.75, *p* < 0.001	*R* ^2^ = 0.99, *p* < 0.001
PER	3.01 ± 0.05	2.97 ± 0.08	2.86 ± 0.03 ^∗^	2.80 ± 0.06	2.30 ± 0.10 ^∗^	<0.05	<0.05	<0.05	*R* ^2^ = 0.57, *p* = 0.006	ns	*R* ^2^ = 0.91, *p* < 0.001	*R* ^2^ = 0.96, *p* < 0.001
LER	5.37 ± 0.09	6.01 ± 0.16	5.75 ± 0.07	5.42 ± 0.11	4.52 ± 0.19	<0.05	<0.05	<0.05	*R* ^2^ = 0.31, *p* = 0.001	*R* ^2^ = 0.88, *p* < 0.001	*R* ^2^ = 0.65, *p* < 0.001	*R* ^2^ = 0.92, *p* < 0.001

*Note:* Values are presented as mean ± SD (*n* = 4 tanks per treatment). *p*‐Values correspond to the effects of meal source (AB vs. PO), inclusion level (25% vs. 50% SMB replacement), and their interaction (meal × inclusion), as determined by GLM including a priori orthogonal contrasts. Significance was established at *p* < 0.05.

Abbreviations: BW, body weight; FCR, feed conversion ratio; FI, feed intake; I, inclusion level; K, condition factor; LER, lipid efficiency ratio; M, mushroom meal; ns, not significant; PER, protein efficiency ratio; SGR, specific growth rate; SL, standard length.

^a^If statistically significant differences were found among groups (ANOVA *p* <  0.05), the polynomial orthogonal contrast was applied and the regression model that best fitted the data was selected and the *R* ^2^ value was indicated. Dietary treatments: CTRL (control diet), AB25 (25% SBM replacement), AB50 (50% SBM replacement), PO25 (25% SBM replacement), and PO50 (50% SBM replacement).

^∗^ *p* < 0.05,  differences from the control diet were assessed using Dunnett^′^s test.

Regarding FI, significant effects of mushroom meal source, inclusion level, and their interaction were observed among dietary groups (*p* < 0.05). The polynomial orthogonal contrasts revealed a quadratic decline in FI in fish fed the PO diets (*R* ^2^ = 0.99, *p* < 0.001), with fish fed the PO50 diet displaying lower FI than CTRL‐fed fish (*p* < 0.05). Similarly, SGR and FCR were significantly affected by mushroom meal source, inclusion level, and their interaction (*p* < 0.05). Both variables displayed a quadratic response to increasing SBM replacement in AB (SGR: *R* ^2^ = 0.88, *p* < 0.001; FCR: *R* ^2^ = 0.76, *p* < 0.001) and PO diets (SGR: *R* ^2^ = 0.99, *p* < 0.001; FCR: *R* ^2^ = 0.95, *p* < 0.001). In particular, fish fed the AB50 and PO50 diets showed reduced SGR compared with CTRL‐fed fish (*p* < 0.05), whereas a higher FCR was only observed in fish fed the PO50 diet (*p* < 0.05). For PER and LER, these indices were significantly affected by mushroom meal source, inclusion level, and their interaction (*p* < 0.05). A linear decline in PER was detected in fish fed the AB diets (*R* ^2^ = 0.57, *p* = 0.006) and a quadratic decline in PO‐fed group (*R* ^2^ = 0.96, *p* < 0.001). Similarly, LER exhibited a quadratic response in both AB (*R* ^2^ = 0.88, *p* < 0.001) and PO diets (*R* ^2^ = 0.92, *p* < 0.001). Fish fed the AB50 and PO50 diets presented lower PER values than CTRL‐fed fish (*p* < 0.05), whereas the lowest LER values were observed in the PO50 group.

At the end of the 60‐day feeding trial, and in agreement with the PCA and 30‐day results, BWf and SLf remained significantly affected by mushroom meal source, inclusion level, and their interaction (*p* < 0.05, Table 4). The polynomial orthogonal contrasts indicated a quadratic decline in BWf with increasing SBM replacement in both AB (*R* ^2^ = 0.94, *p* < 0.001) and PO diets (*R* ^2^ = 0.99, *p* < 0.001). In contrast, SLf exhibited a linear decline in AB diets (*R* ^2^ = 0.58, *p* < 0.001) and a quadratic decrease in both PO diets (*R* ^2^ = 0.98, *p* < 0.001). Accordingly, fish fed the AB50, PO25, and PO50 diets showed lower BWf and SLf than CTRL‐fed fish (*p* < 0.05), with the greatest reduction observed in the PO50 group. K values were significantly influenced by both the meal source and inclusion level (*p* < 0.05), while their interaction was not significant (*p* > 0.05). Polynomial orthogonal contrasts further revealed a linear decrease in K in fish fed the PO diets (*R* ^2^ = 0.54, *p* = 0.01), while no significant trend was detected in AB‐fed groups.

**Table 4:** Survival, growth performance, feed and nutrient efficiencies, and somatic indices of rainbow trout (*Oncorhynchus mykiss*) at day 60 of feeding experimental diets replacing soybean meal with different inclusion levels (I) of mushroom stem meals (M) from *A. bisporus* (AB) and *P. ostreatus* (PO) meals.

Parameter	Experimental diets					Mushroom meal (M)	Inclusion level (I)	M x I	Polynomial orthogonal contrast^a^			
	CTRL	AB25	AB50	PO25	PO50							
									AB		PO	
									Linear	Quadratic	Linear	Quadratic
Survival (%)	100 ± 0.00	100 ± 0.00	100 ± 0.00	100 ± 0.00	100 ± 0.00	ns	ns	ns	—	—	—	—
BW (g)	172.81 ± 2.05	173.57 ± 1.90	160.20 ± 1.57 ^∗^	151.65 ± 1.36 ^∗^	92.37 ± 5.43 ^∗^	<0.05	<0.05	<0.05	*R* ^2^ = 0.66, *p* < 0.001	*R* ^2^ = 0.94, *p* < 0.001	*R* ^2^ = 0.92, *p* < 0.001	*R* ^2^ = 0.99, *p* < 0.001
CV_BW_ (%)	1.18	1.09	0.98	0.90	5.87	—	—	—	—	—	—	—
SL (cm)	21.79 ± 0.22	21.77 ± 0.10	21.29 ± 0.12 ^∗^	20.89 ± 0.12 ^∗^	17.94 ± 0.38 ^∗^	<0.05	<0.05	<0.05	*R* ^2^ = 0.58, *p* < 0.001	*R* ^2^ = 0.75, *p* = 0.04	*R* ^2^ = 0.90, *p* < 0.001	*R* ^2^ = 0.98, *p* < 0.001
K	1.67 ± 0.04	1.68 ± 0.02	1.66 ± 0.03	1.66 ± 0.02	1.60 ± 0.03	<0.05	<0.05	ns	—	—	*R* ^2^ = 0.54, *p* = 0.01	*R* ^2^ = 0.65, *p* = 0.03
CV_K_ (%)	2.19	1.49	1.78	1.06	1.66	—	—	—	—	—	—	—
SGR (% day^−1^)	3.43 ± 0.02	3.44 ± 0.04	3.30 ± 0.02 ^∗^	3.22 ± 0.02 ^∗^	2.38 ± 0.10 ^∗^	<0.05	<0.05	<0.05	*R* ^2^ = 0.61, *p* < 0.001	*R* ^2^ = 0.88, *p* = 0.002	*R* ^2^ = 0.88, *p* < 0.001	*R* ^2^ = 0.99, *p* < 0.001
FCR	0.86 ± 0.01	0.85 ± 0.02	0.93 ± 0.01	0.96 ± 0.01 ^∗^	1.13 ± 0.08 ^∗^	<0.05	<0.05	<0.05	*R* ^2^ = 0.63, *p* < 0.001	*R* ^2^ = 0.91, *p* = 0.002	*R* ^2^ = 0.87, *p* < 0.001	*R* ^2^ = 0.88, *p* = 0.008
FI	4.68 ± 0.01	4.69 ± 0.01	4.68 ± 0.02	4.54 ± 0.02 ^∗^	2.81 ± 0.05 ^∗^	<0.05	<0.05	<0.05	—	—	*R* ^2^ = 0.81, *p* < 0.001	*R* ^2^ = 1.00, *p* < 0.001
PER	2.66 ± 0.03	2.60 ± 0.05	2.47 ± 0.02 ^∗^	2.25 ± 0.02 ^∗^	2.01 ± 0.14 ^∗^	<0.05	<0.05	ns	*R* ^2^ = 0.84, *p* < 0.001	ns	*R* ^2^ = 0.92, *p* < 0.001	ns
LER	5.29 ± 0.05	5.25 ± 0.11	4.95 ± 0.04 ^∗^	4.36 ± 0.04 ^∗^	3.95 ± 0.27 ^∗^	<0.05	<0.05	ns	*R* ^2^ = 0.71, *p* < 0.001	*R* ^2^ = 0.85, *p* = 0.02	*R* ^2^ = 0.90, *p* < 0.001	ns
VSI	15.71 ± 2.77	15.12 ± 1.04	15.15 ± 0.60	17.25 ± 2.30	18.58 ± 1.16	<0.05	ns	ns	—	—	—	—
HSI	1.52 ± 0.09	1.52 ± 0.08	1.64 ± 0.11	1.87 ± 0.28 ^∗^	2.24 ± 0.13 ^∗^	<0.05	<0.05	ns	—	—	*R* ^2^ = 0.78, *p* < 0.001	ns
PFI	1.61 ± 0.29	1.48 ± 0.22	1.59 ± 0.25	1.07 ± 0.12 ^∗^	0.98 ± 0.11 ^∗^	<0.05	ns	ns	—	—	*R* ^2^ = 0.62, *p* = 0.002	ns

*Note:* Values are presented as mean ± SD (*n* = 4 tanks per treatment). *p*‐Values correspond to the effects of meal source (AB vs. PO), inclusion level (25% vs. 50% SMB replacement), and their interaction (meal × inclusion), as determined by GLM including a priori orthogonal contrasts. Significance was established at *p* < 0.05.

Abbreviations: BW, body weight; FCR, feed conversion ratio; FI, feed intake; HSI, hepatosomatic index; I, inclusion level; K, condition factor; LER, lipid efficiency ratio; M, mushroom meal; ns, not significant; PER, protein efficiency ratio; PFI, perivisceral fat index; SGR, specific growth rate; SL, standard length; VSI, viscerosomatic index.

^a^If statistically significant differences were found among groups (ANOVA *p* <  0.05), the polynomial orthogonal contrast was applied and the regression model that best fitted the data was selected and the *R* ^2^ value was indicated. Dietary treatments: CTRL (control diet), AB25 (25% SBM replacement), AB50 (50% SBM replacement), PO25 (25% SBM replacement), and PO50 (50% SBM replacement).

^∗^ *p* < 0.05, differences from the control diet were assessed using Dunnett’s test.

In terms of FI, it was significantly influenced by mushroom meal source, inclusion level, and their interaction (*p* < 0.05). In particular, FI declined quadratically in fish fed the PO diets (*R* ^2^ = 1.00, *p* < 0.001), with reduced FI values compared with their congeners fed the CTRL diet (*p* < 0.05), with the lowest values recorded in the PO50 group. In a pattern similar to that observed on day 30, SGR and FCR were significantly influenced by the source of mushroom meal, the level of inclusion, and their interaction (*p* < 0.05). Polynomial orthogonal contrasts revealed a linear decrease in SGR values and a linear increase in FCR with increasing SBM replacement in AB diets (SGR: *R* ^2^ = 0.61, *p* < 0.001; FCR: *R* ^2^ = 0.63, *p* < 0.001, respectively). In PO diets, SGR exhibited a quadratic decline (*R* ^2^ = 0.99, *p* < 0.001), whereas FCR increased linearly (*R* ^2^ = 0.87, *p* < 0.001). Fish fed the AB50, PO25, and PO50 diets exhibited lower SGR than CTRL‐fed fish (*p* < 0.05), whereas FCR was increased in fish fed the PO25 and PO50 diets (*p* < 0.05). Furthermore, PER and LER were also affected by both meal sources and inclusion levels (*p* < 0.05), whereas their interaction was not significant (*p* > 0.05). A linear reduction in PER was detected in fish fed AB (*R* ^2^ = 0.84, *p* < 0.001) and PO diets (*R* ^2^ = 0.92, *p* < 0.001). Similarly, LER exhibited a linear decline in AB (*R* ^2^ = 0.71, *p* < 0.001) and PO diets (*R* ^2^ = 0.90, *p* < 0.001). Fish fed AB50, PO25 and PO50 diets showed lower PER and LER values compared with the CTRL group (*p* < 0.05).

Among the somatic indices, VSI was affected by mushroom meal source (*p* < 0.05), whereas HSI was affected by both meal source and inclusion level (*p* < 0.05). Polynomial orthogonal contrasts indicated a linear increase in HSI values within the PO diets (*R* ^2^ = 0.78, *p* < 0.001), with fish fed the PO25 and PO50 diets showing higher HSI values than CTRL‐fed fish (*p* < 0.05). In contrast, PFI was affected by mushroom meal source (*p* < 0.05), with lower (*p* < 0.05) values observed in fish fed PO‐based diets compared with fish fed the CTRL treatment. Polynomial orthogonal contrasts indicated a linear reduction in PFI values with increasing SBM replacement in PO‐based diets (*R* ^2^ = 0.62, *p* = 0.002). Survival did not differ among treatments (*p* > 0.05) during the 60‐day feeding trial.

### 3.3. Diet Apparent Digestibility Coefficients

As the PCA indicated, there was a limited contribution of digestibility‐related variables to the overall separation among dietary treatments, suggesting minor dietary effects on nutrient digestibility (Figure 2B). Consistent with this observation, only moderate effects were detected for the ADC values. In particular, DM ADC was affected by mushroom meal source and inclusion level (*p* < 0.05, Table 5), whereas no significant interaction between both factors was observed (*p* > 0.05). Polynomial orthogonal contrasts revealed a linear reduction in DM ADC with increasing SBM replacement in both AB (*R* ^2^ = 0.50, *p* = 0.01) and PO diets (*R* ^2^ = 0.66, *p* < 0.001). In particular, fish fed diets incorporating AB meal presented slightly lower DM ADC values than those fed PO‐based diets, and within the same mushroom meal group, increasing the replacement level of SBM from 25% to 50% reduced DM digestibility. The CP ADC was influenced by mushroom meal source (*p* < 0.05), and a significant interaction between meal source and inclusion level was also detected (*p* < 0.05). A linear decline in CP ADC with increasing SBM replacement was observed in fish fed AB diets (*R* ^2^ = 0.94, *p* < 0.001). Consistent with this pattern and in relation to fish fed CTRL diet, fish fed the AB50 diet showed lower CP ADC values (*p* < 0.05), whereas the remaining experimental groups did not differ from the CTRL group. Diet CL ADC was significantly affected by the mushroom meal inclusion levels (*p* < 0.05), while neither mushroom meal source nor the interaction between factors influenced this parameter (*p* > 0.05). Polynomial orthogonal contrasts revealed a linear reduction in CL ADC with increasing SBM replacement in AB diets (*R* ^2^ = 0.59, *p* = 0.006). Values for GE ADC were significantly affected by mushroom meal source, inclusion level, and a significant interaction between both factors was also detected (*p* < 0.05). A linear reduction in GE ADC with increasing SBM replacement levels was found in trout fed AB diets (*R* ^2^ = 0.57, *p* = 0.005). Despite these statistical effects, no significant differences for this parameter were observed among the experimental groups when compared to the CTRL‐fed fish (*p* > 0.05).

**Table 5:** Apparent digestibility coefficients (ADCs) of experimental diets replacing soybean meal with different inclusion levels (I) of mushroom stem meals (M) from *A. bisporus* (AB) and *P. ostreatus* (PO) meals in rainbow trout (*Oncorhynchus mykiss*).

Experimental diets						Mushroom meal (M)	Inclusion level (I)	M x I	Polynomial orthogonal contrast^a^			
ADC (%)	CTRL	AB25	AB50	PO25	PO50							
									AB		PO	
									Linear	Quadratic	Linear	Quadratic
DM	78.20 ± 1.28	77.08 ± 1.86	75.24 ± 0.89	78.21 ± 0.45	77.48 ± 0.96	<0.05	<0.05	ns	*R* ^2^ = 0.50, *p* = 0.01	ns	*R* ^2^ = 0.66, *p* < 0.001	ns
CP	94.88 ± 0.31	93.33 ± 0.52	91.40 ± 0.42 ^∗^	94.26 ± 0.10	94.15 ± 0.13	<0.05	ns	<0.05	*R* ^2^ = 0.94, *p* < 0.001	ns	—	—
CL	93.51 ± 0.53	92.70 ± 0.80	91.58 ± 1.07	93.31 ± 0.25	92.69 ± 1.24	ns	<0.05	ns	*R* ^2^ = 0.59, *p* = 0.006	ns	—	—
GE	83.78 ± 0.63	83.26 ± 1.19	81.66 ± 0.52	83.50 ± 0.34	83.51 ± 0.49	<0.05	<0.05	<0.05	*R* ^2^ = 0.57, *p* = 0.005	ns	—	—

*Note:* Values are presented as mean ± SD (*n* = 4 tanks per treatment). *p*‐Values correspond to the effects of meal source (AB vs. PO), inclusion level (25% vs. 50% SMB replacement), and their interaction (meal × inclusion), as determined by GLM including a priori orthogonal contrasts.

Abbreviations: CL, crude lipid; CP, crude protein; DM, dry matter; GE, gross energy; I, inclusion level; M, mushroom meal; ns, not significant.

^a^If statistically significant differences were found among groups (ANOVA *p* <  0.05), the polynomial orthogonal contrast was applied and the regression model that best fitted the data was selected and the *R* ^2^ value was indicated. Significance was established at *p* < 0.05. Dietary treatments: CTRL (control diet), AB25 (25% SBM replacement), AB50 (50% SBM replacement), PO25 (25% SBM replacement), and PO50 (50% SBM replacement).

^∗^ *p* < 0.05, differences from the control diet were assessed using Dunnett’s test.

### 3.4. Whole Body Proximate Composition and Nutrient Retention

Whole body proximate composition contributed to the separation of PO‐based diets from the CTRL and AB‐based treatments, as depicted in the PCA (Figure 2C). In agreement with this result, several differences in body composition and nutrient utilization were detected among dietary groups (Table 6). Whole‐body CP content was significantly affected by mushroom meal source (*p* < 0.05), whereas inclusion level and the interaction between factors were not significant (*p* > 0.05). Polynomial orthogonal contrasts revealed a linear increase in whole‐body CP content with increasing SBM replacement in PO‐based diets (*R* ^2^ = 0.70, *p* = 0.001). Consistent with this response, fish fed PO‐based diets had higher whole‐body CP levels compared with those fed both AB‐based diets. In agreement with this pattern and in relation to the CTRL group, fish fed the PO25 and PO50 diets presented higher CP levels than those fish fed the CTRL diet (*p* < 0.05), whereas fish fed AB‐based diets did not differ from the CTRL group.

**Table 6:** Whole‐body proximate composition (dry weight, DW %) and nutrient retention (DW, %) of rainbow trout (*Oncorhynchus mykiss*) fed experimental diets replacing soybean meal with different inclusion levels (I) of mushroom stem meals (M) from *A. bisporus* (AB) and *P. ostreatus* (PO) meals for 60 days.

Composition (% DW)	Experimental diets					Mushroom meal (M)	Inclusion level (I)	M x I	Polynomial orthogonal contrast^a^			
	CTRL	AB25	AB50	PO25	PO50							
									AB		PO	
									Linear	Quadratic	Linear	Quadratic
Whole‐body												
CP	50.93 ± 1.12	50.89 ± 0.57	50.38 ± 1.36	53.39 ± 1.33 ^∗^	54.49 ± 0.49 ^∗^	<0.05	ns	ns	—	—	R^2^ = 0.70, p = 0.001	ns
CL	36.16 ± 2.26	34.45 ± 0.74	36.33 ± 1.33	33.99 ± 2.19 ^∗^	30.30 ± 1.99 ^∗^	<0.05	ns	<0.05	—	—	*R* ^2^ = 0.61, *p* = 0.006	ns
Ash	6.65 ± 1.18	5.94 ± 0.95	6.71 ± 1.01	7.02 ± 0.79	7.67 ± 0.88	ns	ns	ns	—	—	—	—
CHO	1.43 ± 0.12	1.60 ± 0.27	1.74 ± 0.18	1.58 ± 0.07	1.51 ± 0.33	ns	ns	ns	—	—	—	—
Nutrient retention												
CP	42.08 ± 2.66	42.20 ± 1.17	38.68 ± 2.73	35.25 ± 2.21 ^∗^	29.09 ± 2.23 ^∗^	<0.05	<0.05	<0.05	—	—	R^2^ = 0.87, p < 0.001	ns
CL	63.96 ± 3.77	61.91 ± 2.43	60.82 ± 3.95	47.00 ± 2.74 ^∗^	36.06 ± 2.37 ^∗^	<0.05	<0.05	<0.05	—	—	*R* ^2^ = 0.94, *p* < 0.001	ns

*Note:* Values are presented as mean ± SD (*n* = 4 tanks per treatment). *p*‐Values correspond to the effects of meal source (AB vs. PO), inclusion level (25% vs. 50% SMB replacement), and their interaction (meal × inclusion), as determined by GLM including a priori orthogonal contrasts. Significance was established at *p* < 0.05.

Abbreviations: CHO, carbohydrate; CL, crude lipid; CP, crude protein; DM, dry matter; I, inclusion level; M, mushroom meal; ns, not significant.

^a^If statistically significant differences were found among groups (ANOVA *p* <  0.05), the polynomial orthogonal contrast was applied and the regression model that best fitted the data was selected and the *R* ^2^ value was indicated. Dietary treatments: CTRL (control diet), AB25 (25% SBM replacement), AB50 (50% SBM replacement), PO25 (25% SBM replacement), and PO50 (50% SBM replacement).

^∗^ *p* < 0.05, differences from the control diet were assessed using Dunnett’s test.

Whole‐body CL content was affected by mushroom meal source, and the interaction between meal source and inclusion level (*p* < 0.05). A linear decline in CL content with increasing SBM replacement by PO meal was found in fish fed PO diets (*R* ^2^ = 0.61, *p* = 0.006). Accordingly, fish fed the PO25 and PO50 diets exhibited lower CL levels than CTRL‐fed fish (*p* < 0.05). Ash and CHO body contents were not affected by mushroom meal source, inclusion level, or their interaction (*p* > 0.05), and no significant differences among dietary treatments were detected.

The CP and CL retention indexes of rainbow trout fed the experimental diets were influenced by mushroom meal source, inclusion level, and their interaction (*p* < 0.05; Table 6). Polynomial contrasts revealed a linear decline in CP retention (*R* ^2^ = 0.87, *p* < 0.001) and CL retention (*R* ^2^ = 0.94, *p* < 0.001) with increasing SBM replacement in PO‐based diets. Consistent with these responses, fish fed PO‐based diets presented lower CP and CL retention values compared with those fed AB‐based diets, results that were consistent with the separation observed in the PCA (Figure 2C). Indeed, within the same mushroom meal group, increasing the SBM replacement level from 25% to 50% further reduced nutrient utilization efficiency, with the lowest values recorded in both PO groups (*p* < 0.05). Fish fed the PO‐based diets presented reduced CP and CL retention values compared to fish fed the CTRL diet (*p* < 0.05).

### 3.5. Digestive Enzyme Activities

Results from the PCA revealed only moderate discrimination among treatments based on the activities of gastric, pancreatic, and intestinal digestive enzymes (Figure 2D). The activity of PEP was influenced by mushroom meal source (*p* < 0.05, Table 7), whereas inclusion level and the interaction between both factors were not significant (*p* > 0.05). Fish fed PO‐based diets presented lower PEP activity than those fed AB‐based diets; however, no differences were observed when compared with CTRL‐fed fish.

**Table 7:** Total activities of gastric (pepsin), pancreatic (α‐amylase, chymotrypsin, bile salt‐activated lipase, trypsin, total alkaline proteases) and intestinal (alkaline phosphatase, aminopeptidase‐N, maltase and leucine‐alanine peptidase) digestive enzymes in rainbow trout (*Oncorhynchus mykiss*) fed experimental diets replacing soybean meal with different inclusion levels (I) of mushroom stem meals (M) from *A. bisporus* (AB) and *P. ostreatus* (PO) meals for 60 days.

Activity	Experimental diets					Mushroom meal (M)	Inclusion level (I)	M x I	Polynomial orthogonal contrast^a^			
	CTRL	AB25	AB50	PO25	PO50							
									AB		PO	
									Linear	Quadratic	Linear	Quadratic
Gastric enzyme (U/g tissue)												
PEP	40.75 ± 7.07	39.02 ± 9.37	43.91 ± 17.12	25.26 ± 6.39	26.30 ± 6.04	<0.05	ns	ns	—	—	—	—
Pancreatic enzymes (U/g tissue)												
α‐AMY	166.91 ± 58.16	174.73 ± 56.03	210.09 ± 24.03	250.93 ± 41.57	206.54 ± 40.06	ns	ns	ns	—	—	—	—
CHY	5.20 ± 2.37	4.02 ± 1.19	4.14 ± 1.36	6.98 ± 2.12	4.79 ± 2.43	ns	ns	ns	—	—	—	—
LIP	0.12 ± 0.02	0.18 ± 0.05	0.23 ± 0.02 ^∗^	0.21 ± 0.05 ^∗^	0.22 ± 0.03 ^∗^	ns	ns	ns	*R* ^2^ = 0.71, *p* = 0.001	ns	*R* ^2^ = 0.57, *p* < 0.001	ns
TRY	0.43 ± 0.19	0.32 ± 0.09	0.30 ± 0.06	0.52 ± 0.10	0.41 ± 0.15	<0.05	ns	ns	—	—	—	—
TAP	4.68 ± 2.08	3.49 ± 0.70	3.45 ± 1.21	5.67 ± 0.64	4.40 ± 1.85	<0.05	ns	ns	—	—	—	—
Brush border enzymes (mU/g tissue)												
ALPi	0.50 ± 0.22	0.36 ± 0.07	0.46 ± 0.12	0.48 ± 0.16	0.49 ± 0.18	ns	ns	ns	—	—	—	—
APN	0.019 ± 0.00	0.014 ± 0.00	0.015 ± 0.00	0.020 ± 0.01	0.024 ± 0.00	<0.05	ns	ns	—	—	—	—
MAL	790.00 ± 460.00	490.00 ± 230.00	790.00 ± 270.00	1070.00 ± 290.00	2032.00 ± 780.00	<0.05	<0.05	ns	—	—	—	—
Cytosolic enzyme (kU/g tissue)												
LAP	13.62 ± 1.78	13.38 ± 0.59	13.28 ± 0.87	12.70 ± 3.49	13.37 ± 1.68	ns	ns	ns	—	—	—	—

*Note:* Values are presented as mean ± SD (*n* = 4 tanks per treatment). *p*‐Values correspond to the effects of meal source (AB vs. PO), inclusion level (25% vs. 50% SMB replacement), and their interaction (meal × inclusion), as determined by GLM including a priori orthogonal contrasts. Significance was established at *p* < 0.05.

Abbreviations: α‐AMY, α‐amylase; ALPi, alkaline phosphatase; APN, aminopeptidase‐N; CHY, chymotrypsin; I, inclusion level; LAP, leucine‐alanine peptidase; LIP, bile salt‐activated lipase; M, mushroom meal; MAL, maltase; ns, not significant; PEP, pepsin; TAP, total alkaline proteases; TRY, trypsin.

^a^If statistically significant differences were found among groups (ANOVA *p* <  0.05), the polynomial orthogonal contrast was applied and the regression model that best fitted the data was selected and the *R* ^2^ value was indicated. Dietary treatments: CTRL (control diet), AB25 (25% SBM replacement), AB50 (50% SBM replacement), PO25 (25% SBM replacement), and PO50 (50% SBM replacement).

^∗^ *p* < 0.05, differences from the control diet were assessed using Dunnett’s test.

The activities of TRY and TAP were affected by mushroom meal source (*p* < 0.05), with fish fed PO‐based diets presenting higher activities than those fed AB‐based diets, while inclusion level and the interaction between both factors were not significant (*p* > 0.05). The level of LIP did not show significant effects of mushroom meal source, inclusion level, nor their interaction (*p* < 0.05). However, polynomial orthogonal contrasts revealed a linear increase in LIP with increasing SBM replacement in both AB (*R* ^2^ = 0.71, *p* = 0.001) and PO diets (*R* ^2^ = 0.57, *p* < 0.001). Consistent with this response, fish fed the AB50, PO25, and PO50 diets exhibited higher LIP activity than CTRL‐fed fish (*p* < 0.05). The APN was influenced by mushroom meal source (*p* < 0.05), showing slightly higher values in fish fed PO‐based diets. In terms of MAL activity, it was significantly affected by both mushroom meal source and inclusion level (*p* < 0.05), with fish fed PO‐based diets presenting higher activity. In contrast, α‐AMY, CHY, ALPi, and LAP activities were not affected by mushroom meal source, inclusion level, or their interaction (*p* > 0.05).

### 3.6. Blood Biochemical and Hematological Parameters

Blood biochemical and hematological variables showed substantial overlap among dietary treatments, as shown in the PCA (Figure 2E). The two‐way ANOVA and the polynomial orthogonal contrast analysis showed that Ca^2+^, Mg^2+^, PO_4_^3+^, K^+^, ALT/GPT, AST‐SGOT, CK, LDH, LIPp, ALB, GLOB, TP, CHOL, GLU, TBIL, and TG were not significantly affected by mushroom meal source, inclusion level, or their interaction (*p* > 0.05, Table 8).

**Table 8:** Blood biochemical parameters of rainbow trout (*Oncorhynchus mykiss*) fed experimental diets replacing soybean meal with different inclusion levels (I) of mushroom stem meals (M) from *A. bisporus* (AB) and *P. ostreatus* (PO) meals for 60 days.

Parameter	Experimental diets					Mushroom meal (M)	Inclusion level (I)	M x I	Polynomial orthogonal contrast^a^			
	CTRL	AB25	AB50	PO25	PO50							
									AB		PO	
									Linear	Quadratic	Linear	Quadratic
Ca^2+^ (mg dL^−1^)	12.00 ± 0.54	11.83 ± 0.43	11.83 ± 0.19	11.83 ± 0.33	11.75 ± 0.32	ns	ns	ns	—	—	—	—
Cl^-^ (mmol L^−1^)	132.75 ± 0.83	133.00 ± 1.33	132.42 ± 1.66	131.17 ± 0.88	130.92 ± 0.69	<0.05	ns	ns	—	—	*R* ^2^ = 0.49, *p* = 0.007	ns
Mg^2+^ (mg dL^−1^)	3.50 ± 0.14	3.24 ± 0.23	3.33 ± 0.27	3.32 ± 0.10	3.28 ± 0.22	ns	ns	ns	—	—	—	—
PO_4_^3-^ (mg dL^−1^)	20.43 ± 1.24	19.62 ± 0.98	20.05 ± 0.86	20.55 ± 0.93	19.58 ± 0.76	ns	ns	ns	—	—	—	—
K^+^ (mmol L^−1^)	9.10 ± 2.27	7.56 ± 1.51	8.74 ± 1.33	8.88 ± 0.60	9.87 ± 0.64	ns	ns	ns	—	—	—	—
Na^+^ (mmol L^−1^)	142.42 ± 2.38	143.42 ± 2.28	142.25 ± 1.10	141.42 ± 1.55	138.58 ± 0.92	<0.05	<0.05	ns	—	—	*R* ^2^ = 0.50, *p* = 0.02	ns
ALP (U L^−1^)	315.58 ± 42.12	329.17 ± 42.42	323.00 ± 60.92	181.00 ± 29.60 ^∗^	222.58 ± 10.10 ^∗^	<0.05	ns	ns	—	—	*R* ^2^ = 0.37, *p* < 0.001	*R* ^2^ = 0.82, *p* = 0.01
ALT/GPT (U L^−1^)	10.17 ± 1.04	8.50 ± 2.57	9.58 ± 2.41	9.92 ± 1.26	10.00 ± 1.83	ns	ns	ns	—	—	—	—
AST‐SGOT (U L^−1^)	607.88 ± 76.49	579.96 ± 117.08	560.25 ± 93.36	601.25 ± 166.25	660.83 ± 95.07	ns	ns	ns	—	—	—	—
AMY (U L^−1^)	536.75 ± 81.01	490.67 ± 86.50	453.17 ± 117.04	603.50 ± 114.48	646.92 ± 139.45	<0.05	ns	ns	—	—	—	—
CK (kU L^−1^)	142.05 ± 30.31	115.99 ± 80.30	105.25 ± 51.83	78.81 ± 22.71	126.59 ± 24.63	ns	ns	ns	—	—	ns	*R* ^2^ = 0.59, *p* = 0.01
LDH (kU L^−1^)	301.74 ± 67.85	251.93 ± 72.49	252.03 ± 61.38	278.39 ± 72.03	284.98 ± 38.03	ns	ns	ns	—	—	—	—
LIPp (U L^−1^)	178.67 ± 9.12	190.83 ± 36.31	174.17 ± 28.66	168.50 ± 46.23	208.33 ± 39.10	ns	ns	ns	—	—	—	—
ALB (g L^−1^)	19.08 ± 1.10	18.58 ± 1.00	19.17 ± 0.69	18.92 ± 1.17	19.00 ± 0.86	ns	ns	ns	—	—	—	—
GLOB (g L^−1^)	27.50 ± 1.40	26.75 ± 1.69	27.67 ± 1.19	27.42 ± 2.06	27.92 ± 1.40	ns	ns	ns	—	—	—	—
TP (g L^−1^)	46.58 ± 2.49	45.33 ± 2.62	46.83 ± 1.82	46.33 ± 3.19	46.92 ± 2.13	ns	ns	ns	—	—	—	—
CHOL (mg dL^−1^)	274.83 ± 33.87	269.17 ± 13.11	271.00 ± 11.68	264.33 ± 51.91	284.92 ± 12.42	ns	ns	ns	—	—	—	—
CREA (mg dL^−1^)	0.29 ± 0.04	0.26 ± 0.06	0.27 ± 0.02	0.32 ± 0.06	0.34 ± 0.08	<0.05	ns	ns	—	—	—	—
GLU (mg dL^−1^)	42.83 ± 9.48	54.42 ± 8.51	58.75 ± 13.75	50.17 ± 5.59	53.58 ± 7.51	ns	ns	ns	—	—	—	—
TBIL (mg dL^−1^)	0.74 ± 0.19	0.81 ± 0.23	0.77 ± 0.37	0.67 ± 0.25	0.73 ± 0.22	ns	ns	ns	—	—	—	—
TG (mg dL^−1^)	199.75 ± 58.31	201.92 ± 56.73	180.33 ± 39.18	174.25 ± 65.54	235.42 ± 41.84	ns	ns	ns	—	—	—	—

*Note:* Values are presented as mean ± SD (*n* = 4 tanks per treatment). *p*‐Values correspond to the effects of meal source (AB vs. PO), inclusion level (25% vs. 50% SMB replacement), and their interaction (meal × inclusion), as determined by GLM including a priori orthogonal contrasts. Significance was established at *p* < 0.05.

Abbreviations: ALB, albumin; ALP, alkaline phosphatase; ALT/GPT, alanine aminotransferase; AMY, amylase; AST‐SGOT, aspartate aminotransferase; Ca²⁺, calcium; CHOL, cholesterol; CK, creatine kinase; Cl⁻, chloride; CREA, creatinine; GLOB, total globulin; GLU, glucose; I, Inclusion level; K⁺, potassium; LDH, lactate dehydrogenase; LIPp, lipase; M, mushroom meal; Mg²⁺, magnesium; Na⁺, sodium; ns, not significant; PO₄³⁻, phosphorus; TBIL, total bilirubin; TG, triglycerides; TP, total protein.

^a^If statistically significant differences were found among groups (ANOVA *p* <  0.05), the polynomial orthogonal contrast was applied and the regression model that best fitted the data was selected and the *R* ^2^ value was indicated. Dietary treatments: CTRL (control diet), AB25 (25% SBM replacement), AB50 (50% SBM replacement), PO25 (25% SBM replacement), and PO50 (50% SBM replacement).

^∗^ *p* < 0.05, differences from the control diet were assessed using Dunnett’s test.

However, Cl^-^ concentrations were influenced by mushroom meal source (*p* < 0.05). Polynomial orthogonal contrasts revealed a linear decline in Cl^-^ concentration with increasing SBM replacement in PO‐based diets (*R* ^2^ = 0.49, *p* = 0.007). Similarly, Na^+^ levels were affected by both mushroom meal source and inclusion level (*p* < 0.05) and exhibited a linear decline with increasing SBM replacement in PO‐based diets (*R* ^2^ = 0.50, *p* = 0.02). Consistent with these responses, fish fed PO‐based diets exhibited lower Cl^-^ and Na^+^ concentrations than fish fed AB‐based diets. The plasma levels of ALP and AMY were significantly influenced by mushroom meal source (*p* < 0.05) but presented opposite patterns. Regarding plasma ALP, fish fed PO‐based diets presented lower activity than fish fed AB‐based diets, whereas AMY presented higher activities (*p* < 0.05). Polynomial orthogonal contrasts revealed a negative linear response of ALP activity in PO‐based diets (*R* ^2^ = 0.37, *p* = 0.001). Accordingly, fish fed the PO25 and PO50 diets exhibited lower ALP activity than CTRL‐fed fish (*p* < 0.05).

The levels of CREA were also influenced by mushroom meal source (*p* < 0.05), presenting fish fed PO‐based diets higher values than those fed the AB‐based diets but no different from CTRL‐fed fish (*p* > 0.05). Although CK activity was not affected by mushroom meal source, inclusion level, or their interaction (*p* > 0.05), while polynomial orthogonal contrasts revealed a quadratic response in PO‐based diets (*R* ^2^ = 0.59, *p* = 0.01). In the case of GLU, although no individual diet differed significantly from the CTRL according to the Dunnett’s test, the polynomial orthogonal contrast revealed a significant overall dietary effect, indicating a structured response pattern not detectable through pairwise comparisons that pointed toward a higher concentration of plasma GLU in the groups fed with mushrooms, especially in those fed with the AB50 diet (*p* < 0.05).

Regarding hematological parameters, Ht, HB, RBC, WBC, and leukocyte differential counts (LYM, MON, and NEU) were not affected by mushroom meal source, inclusion level, or their interaction (*p* > 0.05, Table 9). In contrast, MCH values were significantly affected by inclusion level (*p* < 0.05), with a quadratic decline in MCH with increasing SBM replacement in PO‐based diets (*R* ^2^ = 0.60, *p* = 0.02). Meanwhile, MCHC was affected by both the mushroom meal source and the inclusion level (*p* < 0.05), with lower values observed in fish fed PO‐based diets.

**Table 9:** Hematological parameters of rainbow trout (*Oncorhynchus mykiss*) fed experimental diets replacing soybean meal with different inclusion levels (I) of mushroom stem meals (M) from *A. bisporus* (AB) and *P. ostreatus* (PO) meals for 60 days.

Parameter	Experimental diets					Mushroom meal (M)	Inclusion level (I)	M x I	Polynomial orthogonal contrast^a^			
	CTRL	AB25	AB50	PO25	PO50							
									AB		PO	
									Linear	Quadratic	Linear	Quadratic
RBC (Nb mL^−1^)	747.50 ± 45.90	706.67 ± 52.14	747.50 ± 30.23	710.83 ± 57.37	799.17 ± 107.65	ns	ns	ns	—	—	—	—
WBC (Nb mL^−1^)	909.52 ± 606.97	1058.50 ± 405.91	469.17 ± 199.77	646.33 ± 428.89	760.00 ± 432.87	ns	ns	ns	—	—	—	—
Ht (%)	53.33 ± 4.06	50.50 ± 4.75	55.75 ± 4.91	56.75 ± 2.18	56.58 ± 3.21	ns	ns	ns	—	—	—	—
Hb (g dL^−1^)	5.33 ± 0.41	5.25 ± 0.34	5.28 ± 0.23	5.19 ± 0.49	4.63 ± 0.30	ns	ns	ns	—	—	—	—
LYM (%)	77.25 ± 10.70	83.42 ± 4.15	78.83 ± 5.80	81.75 ± 7.23	79.33 ± 10.07	ns	ns	ns	—	—	—	—
LYM (mm^−3)^	698.56 ± 417.69	894.01 ± 360.96	376.92 ± 190.99	551.00 ± 408.27	498.27 ± 229.82	ns	ns	ns	—	—	—	—
MON (%)	7.00 ± 2.23	7.25 ± 1.85	5.50 ± 2.73	7.08 ± 3.24	9.58 ± 5.22	ns	ns	ns	—	—	—	—
MON (mm^−3^)	526.85 ± 218.56	753.23 ± 340.55	251.17 ± 148.24	484.52 ± 354.45	504.00 ± 225.88	ns	ns	ns	—	—	—	—
NEU (%)	15.75 ± 10.53	10.83 ± 4.12	15.75 ± 5.90	12.54 ± 7.35	11.08 ± 5.26	ns	ns	ns	—	—	—	—
NEU (mm^−3^)	600.95 ± 532.61	587.87 ± 189.60	665.83 ± 72.65	617.45 ± 226.75	715.33 ± 566.72	ns	ns	ns	—	—	—	—
MCV (fl)	724.35 ± 39.41	724.56 ± 112.02	751.22 ± 95.33	819.82 ± 105.43	722.94 ± 93.33	ns	ns	ns	—	—	—	—
MCH (pg)	72.63 ± 7.51	75.43 ± 10.67	71.08 ± 5.40	74.25 ± 6.84	59.10 ± 4.43	ns	<0.05	ns	—	—	*R* ^2^ = 0.40, *p* = 0.05	*R* ^2^ = 0.60, *p* = 0.02
MCHC (g dL^−1^)	10.08 ± 0.96	10.57 ± 0.34	9.53 ± 0.70	9.19 ± 1.16	8.24 ± 0.61	<0.05	<0.05	ns	—	—	—	—

*Note:* Values are presented as mean ± SD (*n* = 4 tanks per treatment). *p*‐Values correspond to the effects of meal source (AB vs. PO), inclusion level (25% vs. 50% SMB replacement), and their interaction (meal × inclusion), as determined by GLM including a priori orthogonal contrasts. Significance was established at *p* < 0.05. RBC, erythrocytes; WBC, leukocytes.

Abbreviations: Hb, hemoglobin; Ht, hematocrit; I, inclusion level; LYM, lymphocytes; M, mushroom meal; MCH, mean corpuscular hemoglobin; MCHC, mean corpuscular hemoglobin concentration; MCV, mean corpuscular volume; MON, monocytes; NEU, neutrophils; ns, not significant.

^a^If statistically significant differences were found among groups (ANOVA *p* <  0.05), the polynomial orthogonal contrast was applied and the regression model that best fitted the data was selected and the *R* ^2^ value was indicated. Dietary treatments: CTRL (control diet), AB25 (25% SBM replacement), AB50 (50% SBM replacement), PO25 (25% SBM replacement), and PO50 (50% SBM replacement).

^∗^ *p* < 0.05, differences from the control diet were assessed using Dunnett’s test.

### 3.7. LPO, TAC, and Activity of Antioxidant Enzymes

Oxidative stress‐related variables contributed to the separation of PO‐fed fish from the rest of the dietary groups as shown in the PCA, particularly at the highest inclusion level (Figure 2F). Consistent with this pattern, significant differences were observed in LPO and GR levels (Table 10). In particular, LPO was affected by mushroom meal source and inclusion level (*p* < 0.05). There was a quadratic response for LPO in PO‐based diets (*R* ^2^ = 0.54, *p* = 0.01). Fish fed AB‐based diets exhibited lower LPO values than fish fed PO‐based diets, with the highest values recorded in the PO25 group. Furthermore, FRAPS, CAT, and GST activities were not affected by mushroom meal source, inclusion level, or their interaction (*p* > 0.05), whereas GR activity was influenced by mushroom meal source, inclusion level, and their interaction (*p* < 0.05). Polynomial orthogonal contrasts revealed a linear increase in GR activity with increasing SBM replacement in PO‐based diets (*R* ^2^ = 0.93, *p* < 0.001). Consistent with this observation, fish fed PO25 and PO50 diets presented higher GR activity values than CTRL‐fed fish (*p* < 0.05). GPX activity was not affected by mushroom meal source or inclusion level (*p* > 0.05), although a significant interaction between both factors was detected (*p* < 0.05). There was a quadratic response in PO‐based diets (*R* ^2^ = 0.53, *p* = 0.01), with the highest GPX activity observed in fish fed the PO50 diet.

**Table 10:** Lipid peroxidation (nmol MDA/mg protein), ferric reducing antioxidant power (μM FeCl_2_), and activity of antioxidant enzymes (nmol min^−1^ mg protein^−1^) in rainbow trout (*Oncorhynchus mykiss*) fed the experimental diets replacing soybean meal with different inclusion levels (I) of mushroom stem meals (M) from *A. bisporus* (AB) and *P. ostreatus* (PO) meals for 60 days.

Parameter	Experimental diets					Mushroom meal (M)	Inclusion level (I)	M x I	Polynomial orthogonal contrast^a^			
	CTRL	AB25	AB50	PO25	PO50							
									AB		PO	
									Linear	Quadratic	Linear	Quadratic
LPO	26.32 ± 2.34	23.96 ± 2.44	22.49 ± 1.90	31.18 ± 3.57	24.81 ± 2.65	<0.05	<0.05	ns	—	—	ns	*R* ^2^ = 0.54, *p* = 0.01
FRAPS	2584.60 ± 124.48	2299.98 ± 201.78	2797.54 ± 743.57	2441.31 ± 230.84	2211.06 ± 242.14	ns	ns	ns	—	—	—	—
CAT	382.59 ± 67.58	362.08 ± 83.76	409.22 ± 100.10	412.58 ± 57.19	373.38 ± 70.11	ns	ns	ns	—	—	—	—
GR	13.07 ± 1.21	14.49 ± 1.00	14.35 ± 0.90	17.22 ± 0.93 ^∗^	22.71 ± 1.46 ^∗^	<0.05	<0.05	<0.05	—	—	*R* ^2^ = 0.93, *p* < 0.001	*R* ^2^ = 0.94, *p* = 0.003
GPX	25.56 ± 3.57	26.64 ± 2.13	24.59 ± 3.52	23.01 ± 2.35	29.05 ± 1.78	ns	ns	<0.05	—	—	ns	*R* ^2^ = 0.53, *p* = 0.01
GST	78.01 ± 20.03	81.58 ± 7.48	77.40 ± 15.78	86.64 ± 19.25	100.37 ± 19.68	ns	ns	ns	—	—	—	—

*Note:* Values are presented as mean ± SD (*n* = 4 tanks per treatment). *p*‐Values correspond to the effects of meal source (AB vs. PO), inclusion level (25% vs. 50% SMB replacement), and their interaction (meal × inclusion), as determined by GLM including a priori orthogonal contrasts. Significance was established at *p* < 0.05.

Abbreviations: CAT, catalase; FRAPS, ferric reducing antioxidant power; GPX, glutathione peroxidase; GR, glutathione reductase; GST, glutathione S‐transferase; I, inclusion level; LPO, lipid peroxidation; M, mushroom meal; ns, not significant.

^a^If statistically significant differences were found among groups (ANOVA *p* < 0.05), the polynomial orthogonal contrast was applied and the regression model that best fitted the data was selected and the *R* ^2^ value was indicated. Dietary treatments: CTRL (control diet), AB25 (25% SBM replacement), AB50 (50% SBM replacement), PO25 (25% SBM replacement), and PO50 (50% SBM replacement).

^∗^ *p* < 0.05, differences from the control diet were assessed using Dunnett’s test.

### 3.8. Liver Morphology

**Figure 3:**
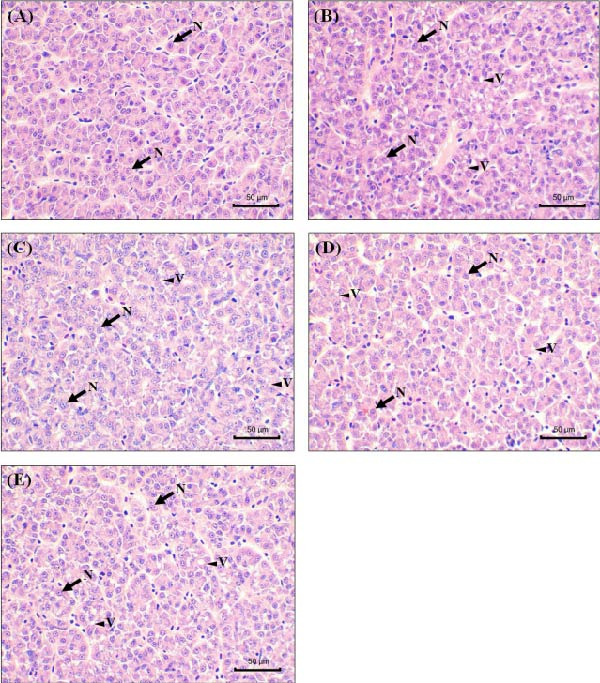
Liver morphology of rainbow trout (*Oncorhynchus mykiss*) fed experimental diets replacing soybean meal with graded levels of *A. bisporus* (AB) and *P. ostreatus* (PO) meals for 60 days. (A–E) Hematoxylin and eosin (H&E) stain, (A) CTRL, (B) AB25, (C) AB50, (D) PO25, (E) PO50. (N, arrow) hepatocytes nuclei, (V, arrowhead) hepatocyte cytoplasmic vacuole. Scale bar 50 μm. Dietary treatments: CTRL (control diet), AB25 (25% SBM replacement), AB50 (50% SBM replacement), PO25 (25% SBM replacement), and PO50 (50% SBM replacement).

The histological evaluation of H&E‐stained liver sections at the end of the feeding trial showed a well‐organized morphological pattern and regular hepatocyte size, with nuclei properly aligned around sinusoidal spaces across experimental diets (Figure 3). Although fish fed mushroom‐based diets showed slight changes in hepatocyte cytoplasmic vacuolation and nuclear positioning, this histological organization of the hepatic parenchyma was consistent with normal liver morphology, showing no significant deviations from the CTRL diet (*p* > 0.05).

## 4. Discussion

The present study demonstrates that mushroom stem‐based ingredients can serve as sustainable nutritional replacers of SBM in rainbow trout aquafeeds, with their applicability strongly dependent on the mushroom species, dietary inclusion levels, and duration of the administration period. This overall pattern was supported by the PCA results, which revealed a clear separation among dietary groups and confirmed that the observed responses were not isolated effects but part of an integrated physiological response of rainbow trout fed experimental diets. In particular, PCA highlighted a distinct clustering of fish fed PO‐based diets, especially at higher inclusion levels, whereas CTRL and AB‐based diets displayed similar responses regardless of the set of performance, nutritional, and biochemical indicators considered. The partial replacement of SBM, particularly at 25% with the AB mushroom stem meal, supported growth performance and feed utilization comparable to the CTRL diet, whereas higher replacement levels (up to 50%), and using both PO mushroom stem‐based diets, progressively impaired growth and feed efficiency over time. These contrasting responses underscore a pronounced species‐specific mushroom effect, which may determine their long‐term suitability as SBM replacers. Indeed, the progressive decline in performance at higher inclusion levels suggests that, beyond nutritional composition, cumulative effects associated with bioactive compounds or ANFs in mushroom stem meals may contribute to the observed time‐dependent responses and will determine the degree of physiological adaptation of rainbow trout to these novel ingredients. In addition, biochemical composition, including fiber fractions, chitin content, the presence of bioactive compounds such as vitamins B and D2, β‐glucans, essential minerals, terpenes, phenolic compounds, and sterols [52], may influence not only nutrient digestibility but also feed acceptance in carnivorous fish species such as rainbow trout. In the present study, we focused on meals derived not from the edible portions of mushrooms but rather from their by‐products. In certain instances, particularly with regard to AB, these by‐products may contain remnants of cultivation substrate that are rich in ash and structural carbohydrates such as lignin and cellulose, thereby increasing the mineral fraction and indigestible components of the final ingredient [53]. Under present experimental conditions, in the short term (30 days of feeding), fish fed the AB25 and PO25 diets (25% replacement of SBM) presented similar growth performance and feed efficiency comparable to the CTRL group, together with a trend to improved LER values, indicating good short‐term tolerance and effective utilization of these ingredients at moderate inclusion levels. Regarding the AB meal, the results observed are consistent with reports in channel catfish (*Ictalurus punctatus*) and beluga (*Huso huso*) juveniles fed AB‐based diets [54, 55]. In fact, the trend to a more efficient use of dietary lipids observed aligns with findings from other animal models, suggesting that AB may modulate lipid metabolism, such as the regulation of cholesterol synthesis and fatty acid metabolism via bioactive compounds like sterols and β‐glucans that modulate gene expression related to lipid processing and bile acid metabolism [56–60]. However, higher dietary levels (AB50; 50% SBM replacement) resulted in a gradual time‐dependent decline in growth performance but without reducing the FI values. This dose‐time‐dependent effect suggests an exceedance of the capacity for long‐term metabolic adaptation, which on one hand, could be related to the relatively high content of bioactive polysaccharides such as β‐glucans [61] and/or mannans that may be overstimulating the gut‐associated lymphoid tissue (GALT) response. However, it also may be linked to the decreased protein digestibility of the AB meal reported previously by Saromines et al. [17], which may become physiologically relevant during prolonged feeding with this meal. Indeed, the increasing dietary ash levels and complex polysaccharides (e.g., β‐glucans and mannans) dilute nutrient density, reduce digestibility, and influence gut microbiota composition and metabolic activity [17, 18, 61, 62]. In line with this, at the end of the feeding trial, fish fed the AB50 diet presented lower PER and LER values than fish fed the reference diet, which aligns with the results of ADC values, where CP ADC decreased at higher AB inclusion levels, which are in agreement with previous studies [17].

In contrast, rainbow trout showed lower short‐term adaptability to PO‐based diets. While fish fed the PO25 diet presented growth and FI values similar to the CTRL group during the initial feeding period, early signs of reduced performance were already evident at higher inclusion levels (50% SBM replacement; PO50), with prolonged exposure resulting in reduced FI and nutrient utilization values that ultimately compromised somatic growth and body condition. Instead, these results point toward a hedonic regulation of FI [63], which was likely masked in a previous digestibility‐focused study in which diets contained higher FM levels compared to the current diets [17]. Notably, these responses were dependent on both inclusion levels and feeding duration, indicating a clear time‐dose effect, with reduced fish performance in both PO treatments primarily driven by decreased FI rather than impaired digestibility, as similar ADC values were observed among dietary groups. These results suggest that nutrient supply was limited at higher inclusion levels and under prolonged exposure, thereby reducing the overall energy intake and shifting absorbed nutrients toward maintenance processes rather than somatic growth. In agreement with nutritional studies conducted in Nile tilapia (*O. niloticus*) [25] and Amur catfish (*Silurus asotus*) [64], PO inclusion above moderate levels reduced growth, primarily due to decreased FI values. Several factors may explain these effects, including the presence of ANFs in mushroom stem meals such as phytic acid and tannins, volatile compounds affecting feed aroma and flavor [65–67], and the type and quantity of dietary fiber, which affect feed acceptance as well as nutrient absorption in carnivorous fish [68, 69]. In this context, *Pleurotus* species are also known to contain bioactive compounds with immunomodulatory properties [70]. As previously indicated, excessive concentrations of these compounds may compromise the fish local immune function. This may occur not only via response to damage‐associated molecular patterns (DAMPs) related to the presence of ANFs but also to pathogen‐associated molecular patterns (PAMPs) associated with the higher content of complex β‐glucans in PO compared to AB, leading to diminished gut functionality [71] and an increased energy demand. This, in conjunction with reduced FI, may account for the observed reduction in growth and PFI, in agreement with similar studies in other animal models [72]. Targeted immunological studies are being conducted to confirm this mechanism, as this fact can also contribute to the inclusion‐level‐dependent response observed for HSI values in fish fed mushroom‐based diets. Since increases in the relative size of the liver may be related to changes in hepatic glycogen and/or lipid deposition [17, 73] but also with intensified protein turnover due to changes in dietary amino acid profiles [74, 75]. However, when considered alongside the observed reductions in growth, FI, and nutrient utilization efficiency, these findings suggest heightened hepatic metabolic activity rather than enhanced energy storage. Indeed, β‐glucans and other fungal polysaccharides present in PO may trigger β‐oxidation or modify gut microbiota‐mediated nutrient partitioning [17, 18, 61], inducing a redistribution of energy, which may lead to reduced peripheral fat deposition and altered lipid and protein hepatic metabolism [76–78].

Consistent with these findings, whole‐body composition revealed a clear effect of the mushroom meal type on total CP and CL contents, whereas ash and CHO were unaffected. Fish fed both PO diets showed the highest whole‐body CP content, results that are in agreement with previous studies in rainbow trout fed diets containing gargal mushroom (*Grifola gargal*) [79]. In particular, the PO‐fed group showed higher body CP than AB‐fed fish, which may be likely due to both reduced growth, as smaller fish typically present higher protein, as well as lower CL contents [79, 80] due to reduced FI, under which situation nutrient allocation prioritizes maintaining body protein over energy storage [81]. On the other hand, fish fed the AB diets maintained whole‐body CP comparable to the CTRL group and intermediate CL levels, indicating a preservation of whole‐body nutrient composition [23, 25].

These results highlight the importance of taking into consideration the feeding duration when assessing novel feed ingredients and indicate that both mushroom species and the level of SBM replacement should be carefully optimized to maintain sustained growth performance in rainbow trout in accordance with the ADC results. This effect was particularly evident in DM, energy, and CP digestibility, indicating a mushroom species‐specific response. A significant reduction in ADCs was observed in fish fed the AB50 diet compared to fish fed the CTRL diet, whereas no such effect occurred in PO‐based diets. The reduced protein ADC at high levels of AB inclusion may indicate a diminished nutrient bioavailability, coupled with increased concentrations of lignified fiber, chitin [82], and ANFs that become proportionally more significant as inclusion levels rise. Additionally, the relatively high ash content of this by‐product may further limit nutrient utilization [17]. In contrast, the reduction in CL digestibility observed at higher inclusion levels appears primarily related to the dilution of more digestible lipid sources by mushroom stem‐meal biomass rather than to diet type or interaction effects. However, the abovementioned changes in ADC values may be considered minor and not directly associated with dietary fiber content, which in tested mushroom‐stem meals was below 10%, a value considered safe for rainbow trout [83]. In this sense, as fiber was not directly digested by rainbow trout, it may be fermented by the gut microbiota, modulating gut condition as pointed out by Reinoso et al. [61] when evaluating the effects of AB diets on rainbow trout gut microbiota. These digestibility patterns were partly reflected in nutrient retention, a key indicator of dietary assimilation efficiency [84], which was significantly affected by the type of mushroom meal, inclusion level, and their interaction. Values of CP and CL retention in fish fed both AB diets were comparable to those fed the CTRL diet; however, CP retention tended to be reduced in absolute values for the AB50 group, in agreement with previous in vitro digestibility studies [17]. In contrast, PO‐fed fish showed a gradual decline in CP and CL retention as SBM replacement increased, in parallel with reduced FI, nutrient efficiency, and poorer growth performance. This negative dose–response relationship suggests that elevated levels of PO inclusion compromise the efficiency of protein and lipid utilization due to lower FI and nutrient assimilation. As pointed out by Reinoso et al. [61], the inclusion of mushroom stem meal from PO also triggered a mild dysbiosis in the gut of rainbow trout, reinforcing the negative effects of PO inclusion in rainbow trout aquafeeds. As mentioned for AB‐based treatments, this fact could be related not only to a nutritional component but also to the presence of bioactive compounds and ANFs in PO stem‐based meals. Similar trends have been reported by Pascual et al. [79], supporting the existence of an optimal inclusion threshold for mushroom‐based ingredients.

Regarding the impact of mushroom meals on the activity of digestive enzymes, the present results showed that mushroom meal type as well as their inclusion level in rainbow trout diets did not compromise the activity of α‐AMY, CHY, LIP, ALPi, and LAP. On the contrary, the inclusion of PO meals at both tested levels significantly reduced the activity of PEP and increased the activity of TRY and TAP compared to fish fed mushroom‐based diets. In this sense, as samples for the determination of digestive enzymes were taken after overnight fasting, the reduction in PEP activity may not be attributed to the presence of ANFs in the PO meal rather than to an adaptive response of rainbow trout to prolonged reduced FI and to the endocrine regulation of gut function [85]. This hypothesis may be supported by the fact that vagal sensory neurons monitor stomach volume by means of mechanosensory cells, assisting in the regulation of the production and secretion of gastric secretions, including pepsinogen and hydrochloric acid [86, 87]. Thus, reduced FI levels found in the PO25 and PO50 groups due to palatability issues may have resulted in an adaptative response of the stomach to feeding [88]. The decrease in PEP production may be counterbalanced by an increase in TAP and TRY in fish fed both PO diets [36, 89], a hypothesis that may be supported by similar protein ADC values observed among dietary groups. In terms of other pancreatic enzymes, no significant effects on α‐AMY activity were observed for both mushroom meals tested; however, as the level of mushroom‐stem meal inclusion increased, the activity of LIP increased compared to the CTRL group, especially in fish fed PO diets. The increase in LIP production levels may be interpreted as a compensatory mechanism to the lower numerical lipid ADC values found in the AB50 and PO50 groups [90–92], although these results require further investigation, as if food is not present in the gut, which is the situation in the current study, pancreatic digestive enzymes are accumulated in the pancreas in the form of zymogens [89].

Concerning intestinal enzymes, results indicated that the inclusion of AB and PO meals at both levels tested did not affect the activity of the brush border enzyme ALP, nor that of the cytosolic protease LAP. These results indicate that the inclusion of such ingredients did not cause any damage to the intestinal epithelium, as previously demonstrated when these ingredients were tested at higher levels of inclusion [17] nor did it affected peptide digestion into free amino acids within enterocytes [93]. In contrast, the inclusion of PO meal resulted in changes in the MAL and APN activities in fish fed PO‐based diets compared to AB‐fed fish. As MAL is involved in the digestion of CHO such as maltose, maltotriose, or α‐1,4‐oligoglucans that may be present in the PO meal or derived from α‐AMY digestion, the higher activity of MAL in the brush border of enterocytes in fish fed PO‐based diets may be considered an adaptation to the CHO content of this mushroom meal and the adaptation of the intestinal mucosa to the diet [61, 94, 95]. Furthermore, the increase in the activity of APN, a brush‐border protease involved in cleaving oligopeptides derived from pancreatic and gastric proteases into free amino acids, may be considered a counterbalance digestive mechanism to guarantee protein digestion and assimilation, similarly to that observed in pancreatic alkaline proteases [95, 96].

Under current experimental conditions, the overall stability of plasma biochemical and hematological profiles indicates that the dietary inclusion of AB and PO by‐product meals did not compromise the physiological condition and health of rainbow trout, in agreement with similar studies in this and other freshwater species [17, 97]. In this regard, only small variations associated with the mushroom meal used or the level of inclusion were observed for some parameters. For instance, the changes observed in plasma Na^+^ and Cl^−^ concentrations after feeding the mushroom‐based diets were particularly evident in fish fed PO‐based diets in agreement with their poorest growth performance. This fact suggests minor osmoregulatory adjustments as observed in both fish and mammals exposed to *Pleurotus* spp. extracts or dietary modulation [98–101] given that no other major ion concentrations (Ca^2+^, Mg^2+^, K^+^, PO_4_^3+^) were affected. Indeed, plasmatic ALP was also reduced in fish fed PO‐based diets in relation to AB‐fed fish but also to the CTRL treatment. In fish, as in higher vertebrates, plasma ALP activity represents the combined contribution of isoenzymes primarily derived from the liver, bone, and intestine. Although distinct isoforms are produced by hepatic (tissue nonspecific) and intestinal tissues, the hepatic isoform predominates in circulation and plays a central role in regulating overall plasma ALP levels and the clearance of ALPi [102]. In this sense, changes in plasma ALP levels are generally associated with hepatic conditions [103, 104]. In fact, despite the values observed for plasma ALP, the stability of ALT/GPT and AST/SGOT ratios, along with the absence of significant changes in liver histological organization, points to good hepatic health in fish fed the PO‐based diets and may indicate that lower ALP levels may be attributed to the lower size of fish but also, although to a lesser extent, to associated changes in intestinal ALP due to a potential ANF‐related effect and consequent changes in intestinal microbiota composition [61, 105–107]. Indeed, while overall metabolic homeostasis appeared to be maintained, elevated GLU levels in fish fed mushroom‐supplemented diets suggest a response to altered CHO metabolism, which is in agreement with recent results addressing microbiota profiles [61, 105–107].

The levels of Hb remained stable, with a decreasing trend in MCH and MCHC at high levels of SBM replacement, especially when replaced by PO, probably related to the reduction in nutrient availability detected without compromising oxygen transport or immunity [108, 109], consistent with previous studies on *Pleurotus* spp. supplementation in rainbow trout and Nile tilapia (*O. niloticus*) [110, 111]. However, again, this observation may also be partially related to the presence of ANFs, such as phytates, which could potentially impair iron absorption [108]. Although Hb levels remained unchanged, a reduced availability of iron cannot be ruled out as a contributing factor to the observed decrease in MCH and MCHC values.

Oxidative stress‐related results revealed significant effects of the type of mushroom meal and inclusion levels on LPO levels and GR activity, as well as significant interactions for GR and GPX, highlighting differential redox modulation according to the ingredient source and level of SBM replacement. Fish fed PO‐based diets exhibited an LPO nonlinear response, with elevated levels in the PO25 group, followed by a reduction at the higher inclusion level. This decrease coincided with increased GR activity, together with a tendency toward higher GPX and GST activities, suggesting a coordinated regulation of the glutathione redox cycle in PO50‐fed fish [74, 112]. This adaptation may represent a compensatory mechanism aimed at enhancing peroxide detoxification and preserving redox homeostasis through more efficient recycling of reduced glutathione, potentially in response to an oxidative challenge or to elevated levels of bioactive dietary compounds, including β‐glucans and phenolic compounds but also to a potential microbiota‐ingredient interaction, as PO diets resulted in mild intestinal dysbiosis [61].

## 5. Conclusion

The present study demonstrates that the suitability of mushroom stem meals as replacements for SBM in rainbow trout diets is strongly dependent on mushroom species and inclusion levels, with distinct impacts on growth performance, nutrient utilization, digestive physiology, and metabolic homeostasis. Moderate replacement of SBM with AB at 25% supported growth, feed efficiency, and nutrient utilization comparable to conventional SBM‐based diets, whereas higher SBM replacement (50%; AB50) gradually impaired growth performance and feed efficiency. This decline likely reflects reduced nutrient digestibility and bioavailability, compounded by structural polysaccharides, ANFs, and ash content at higher inclusion levels. In contrast, replacement of SBM with PO led to more pronounced declines in growth and nutrient efficiency, particularly at 50% (PO50) replacement, primarily driven by reduced FI. Reduced intake appeared related to palatability limitations, elevated dietary fiber and complex carbohydrates, and potential bioactive compounds or ANFs triggering enhanced hepatic metabolism and antioxidant defenses, including increased GR activity, rather than systemic stress or impaired digestibility. Furthermore, the inclusion of mushroom‐stem meals modulated the activity of gastric, pancreatic, and intestinal enzymes in order to guarantee nutrient assimilation and availability as ADC values indicated. Among the treatments tested, AB at 25% SBM replacement (50 g kg^−1^) represented the most balanced dietary strategy for rainbow trout, maintaining growth, feed and nutrient efficiency, digestive function, and metabolic stability comparable to commercial SBM‐based diets. Beyond nutritional performance, the use of mushroom stem meals supports circular bioeconomy and zero‐waste principles, providing a sustainable approach to valorize agro‐industrial by‐products. Future research should explore the immunological and long‐term physiological effects of AB and PO diets and investigate strategies to enhance the functional properties and palatability of mushroom meals, particularly PO, through processing techniques such as fermentation, enzymatic treatment, or feed attractant supplementation, to maximize their nutritional efficiency and acceptance in aquafeeds.

## Author Contributions

**Carl John Saromines**: writing of the original draft, visualization, methodology, formal analysis, data curation. **Enric Gisbert**: writing – review and editing, methodology, supervision, formal analysis, project administration, funding acquisition, project administration. **Samira Reinoso**: formal analysis, writing – review and editing. **Maria Luisa Tello Martín and Margarita Pérez Clavijo**: writing – review and editing, methodology (mushroom meal production), funding acquisition. **Silvia Torrecillas**: writing – review and editing, supervision, methodology, data curation, formal analysis, project administration.

## Funding

This work was conducted within the GreenBlueCircle project (Grant TED2021‐132054B‐C21/C22) funded by the Ministerio de Ciencia e Innovación (Spain). Carl John Saromines was supported by a predoctoral grant from IRTA. Silvia Torrecillas is financed by a Ramón y Cajal fellowship (Grant RYC2021‐031414‐I) funded by MICIU/AEI.

## Ethics Statement

The study followed the guidelines and received approval from the Ethical Committee of the Institute for Research and Technology in Food and Agriculture (IRTA, Spain) for the use of laboratory animals (E‐10/2020), Spanish laws (32/2007 and RD 1201/2015), Generalitat de Catalunya (CEEA 219/2020), and in accordance with EU2010/63 guidelines (Guiding Principles for Biomedical Research Involving Animals).

## Conflicts of Interest

The authors declare no conflicts of interest.

## Supporting Information

Additional supporting information can be found online in the Supporting Information section.

## Supporting information


**Supporting Information 1** Figure 1: Temporal variation of water quality parameters monitored throughout the 60‐day feeding trial.

**Supporting Information 2** Table 1: Diagnostic statistics (Shapiro–Wilk and Levene’s tests) and plots for all analyzed response variables.

## Data Availability

The raw data supporting the conclusions of this article will be made available by the authors upon request.
