# Supplementary material for: Valorization of Mushroom By‐Products From Agaricus bisporus and Pleurotus ostreatus as Novel Feed Ingredients for Rainbow Trout (Oncorhynchus mykiss) Diets
Source: Aquac Nutr. 2026 Aug 3;2026:6294426. doi: 10.1155/anu/6294426 (PMC13434034; doi:10.1155/anu/6294426)
Supplement: Supplementary file 1 [file ANU-2026-6294426-s001.pdf]

Supplementary Table 1. Diagnostic statistics for all analyzed variables. Residual normality was assessed using the Shapiro–Wilk test, and homogeneity of variance was evaluated using Levene's test based on the factors Diet type and Inclusion level. Results are presented for each response variable as test statistics and associated p-values.

	Shapiro-Wilk Test		Levene's Test	
**Variable**	***F value***	***p value***	***F value***	***p value***
Initial fish performance data				
BW (g)	0.96	0.57	0.71	0.60
SL (cm)	0.96	0.56	0.13	0.97
K	0.92	0.08	0.29	0.88
Day 30 fish performance data				
BW (g)	0.96	0.53	0.56	0.70
SL (cm)	0.95	0.30	1.64	0.22
K	0.98	0.94	0.97	0.45
SGR (% day $^{-1}$)	0.96	0.50	3.82	0.02
FCR	0.94	0.27	2.47	0.09
FI	0.77	0.00	3.09	0.05
PER	0.96	0.53	0.82	0.53
LER	0.97	0.71	0.84	0.52
Day 60 fish performance data				
BW (g)	0.97	0.77	6.15	0.00
SL (cm)	0.94	0.24	3.14	0.05
K	0.97	0.75	0.33	0.85
SGR (% day $^{-1}$)	0.89	0.03	2.14	0.13
FCR	0.78	0.00	4.47	0.01
FI	0.91	0.07	1.47	0.26
PER	0.88	0.02	4.41	0.01
LER	0.88	0.02	4.45	0.01
VSI	0.91	0.07	0.57	0.69
HSI	0.95	0.31	1.31	0.31
PFI	0.97	0.68	1.20	0.35
Apparent digestibility coefficients (ADC)				
Dry matter	0.96	0.62	0.40	0.80
Protein	0.94	0.29	0.57	0.69
Lipid	0.97	0.84	2.10	0.13
Energy	0.96	0.55	0.99	0.44
Whole-body proximate composition				
Protein	0.97	0.75	1.90	0.16
Lipid	0.98	0.86	1.53	0.24
Ash	0.95	0.37	0.20	0.93
Carbohydrate	0.98	0.94	1.85	0.17
Nutrient retention				
Protein	0.97	0.79	0.27	0.89
Lipid	0.98	0.89	0.35	0.84

Digestive enzymes activities				
Pepsin	0.98	0.92	2.21	0.12
Alpha-amylase	0.96	0.50	0.33	0.86
Chymotrypsin	0.95	0.43	0.53	0.72
Bile salt-activated lipase	0.95	0.41	1.91	0.16
Trypsin	0.98	0.87	0.51	0.73
Total alkaline proteases	0.96	0.63	0.82	0.53
Alkaline phosphatase	0.94	0.20	1.60	0.23
Aminopeptidase-N	0.89	0.03	2.21	0.12
Maltase (U/g tissue)	0.99	0.99	2.85	0.06
Leucine-alanine peptidase	0.93	0.19	1.28	0.32
Blood biochemical parameters				
Calcium (mg dL^{-1})	0.96	0.45	0.31	0.86
Chlorides (mmol L^{-1})	0.95	0.37	0.36	0.83
Magnesium (mg dL^{-1})	0.95	0.44	0.49	0.74
Phosphorus (mg dL^{-1})	0.90	0.04	0.39	0.81
Potassium (mmol L^{-1})	0.86	0.01	0.30	0.87
Sodium (mmol L^{-1})	0.94	0.26	0.86	0.51
ALP (U L^{-1})	0.96	0.55	0.83	0.53
ALT/GPT ((U L^{-1})	0.97	0.70	0.81	0.54
AST-SGOT (U L^{-1})	0.94	0.29	0.20	0.93
Amylase (U L^{-1})	0.97	0.77	0.20	0.93
Creatine kinase (kU L^{-1})	0.97	0.81	5.06	0.01
Ldh (kU L^{-1})	0.95	0.39	0.54	0.71
Lipase (U L^{-1})	0.95	0.36	1.85	0.17
Albumin (g L^{-1})	0.95	0.43	0.12	0.97
Total globulin (g L^{-1})	0.95	0.31	0.17	0.95
Total protein (g L^{-1})	0.92	0.12	0.15	0.96
Cholesterol (mg dL^{-1})	0.92	0.10	1.85	0.17
Creatinine (mg dL^{-1})	0.97	0.66	1.85	0.17
Glucose (mg dL^{-1})	0.92	0.08	0.18	0.95
Total bilirubin (mg dL^{-1})	0.96	0.60	1.01	0.43
Triglycerides (mg dL^{-1})	0.93	0.18	0.11	0.98
Hematological parameters				
Erythrocytes (Nb mL^{-1})	0.95	0.40	1.44	0.27
Leukocytes (Nb mL^{-1})	0.95	0.39	0.45	0.77
Hematocrit (%)	0.88	0.01	0.52	0.72
Hemoglobin (g dL^{-1})	0.95	0.30	0.71	0.60
Lymphocytes (%)	0.95	0.40	0.63	0.65
Lymphocytes ($mm^{-3)}$	0.95	0.40	0.40	0.81
Monocytes (%)	0.91	0.07	0.79	0.55
Monocytes (mm^{-3})	0.97	0.75	1.20	0.35

Neutrophils (%)	0.95	0.43	0.59	0.67
Neutrophils (mm^{-3})	0.88	0.02	0.69	0.61
MCV (fl)	0.97	0.72	0.96	0.46
MCH (pg)	0.93	0.16	0.40	0.81
MCHC (g dL^{-1})	0.95	0.32	0.46	0.76
Lipid peroxidation and activity of antioxidant enzymes				
LPO	0.93	0.19	0.36	0.84
FRAPS	0.88	0.02	1.07	0.41
CAT	0.95	0.32	0.85	0.51
GR	0.97	0.66	0.05	1.00
GPX	0.98	0.93	0.73	0.59
GST	0.91	0.07	1.02	0.43

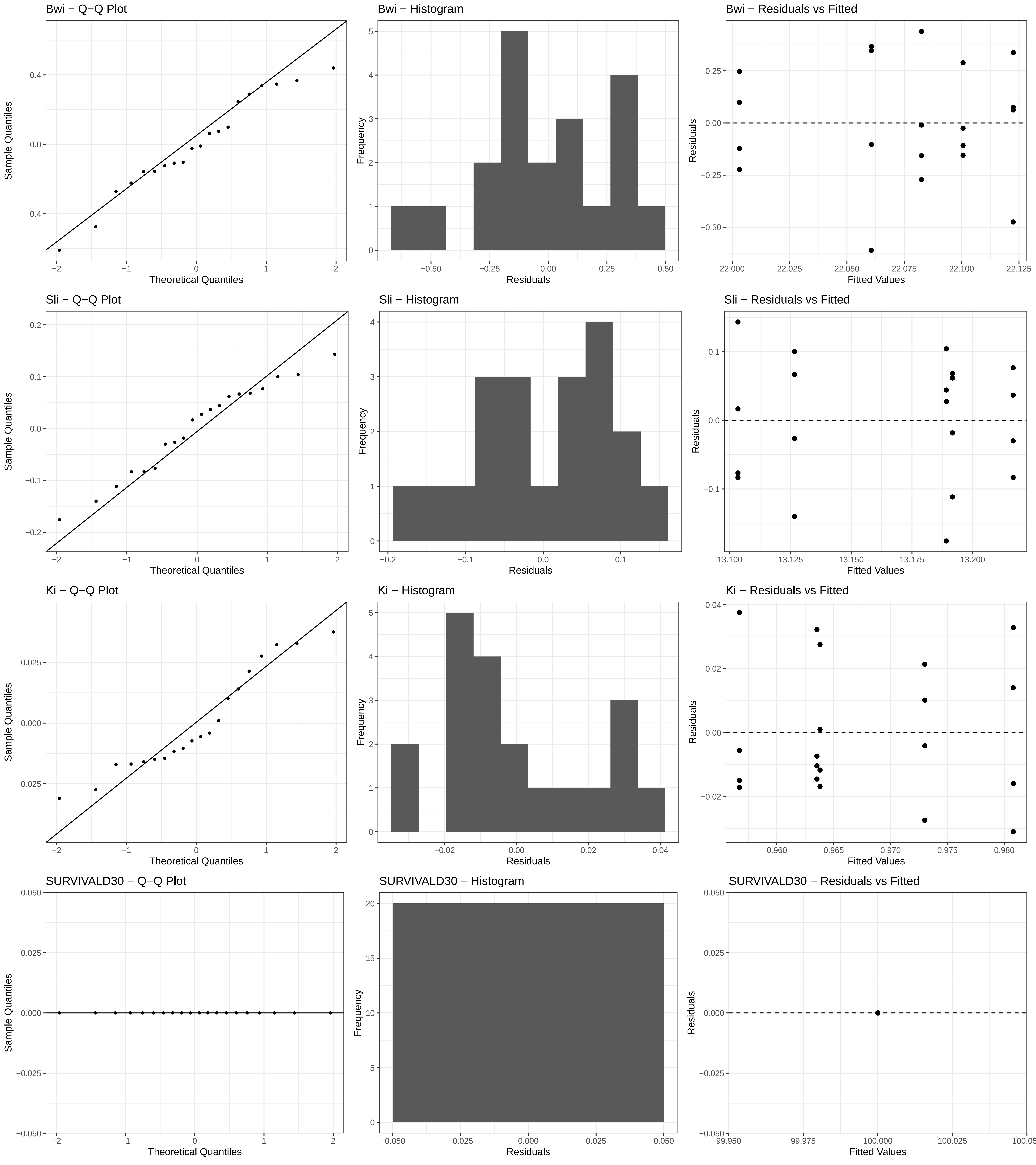
Bwi − Q−Q Plot
Sample Quantiles
Theoretical Quantiles
Bwi − Histogram
Frequency
Residuals
Bwi − Residuals vs Fitted
Residuals
Fitted Values
Sli − Q−Q Plot
Sample Quantiles
Theoretical Quantiles
Sli − Histogram
Frequency
Residuals
Sli − Residuals vs Fitted
Residuals
Fitted Values
Ki − Q−Q Plot
Sample Quantiles
Theoretical Quantiles
Ki − Histogram
Frequency
Residuals
Ki − Residuals vs Fitted
Residuals
Fitted Values
SURVIVALD30 − Q−Q Plot
Sample Quantiles
Theoretical Quantiles
SURVIVALD30 − Histogram
Frequency
Residuals
SURVIVALD30 − Residuals vs Fitted
Residuals
Fitted Values

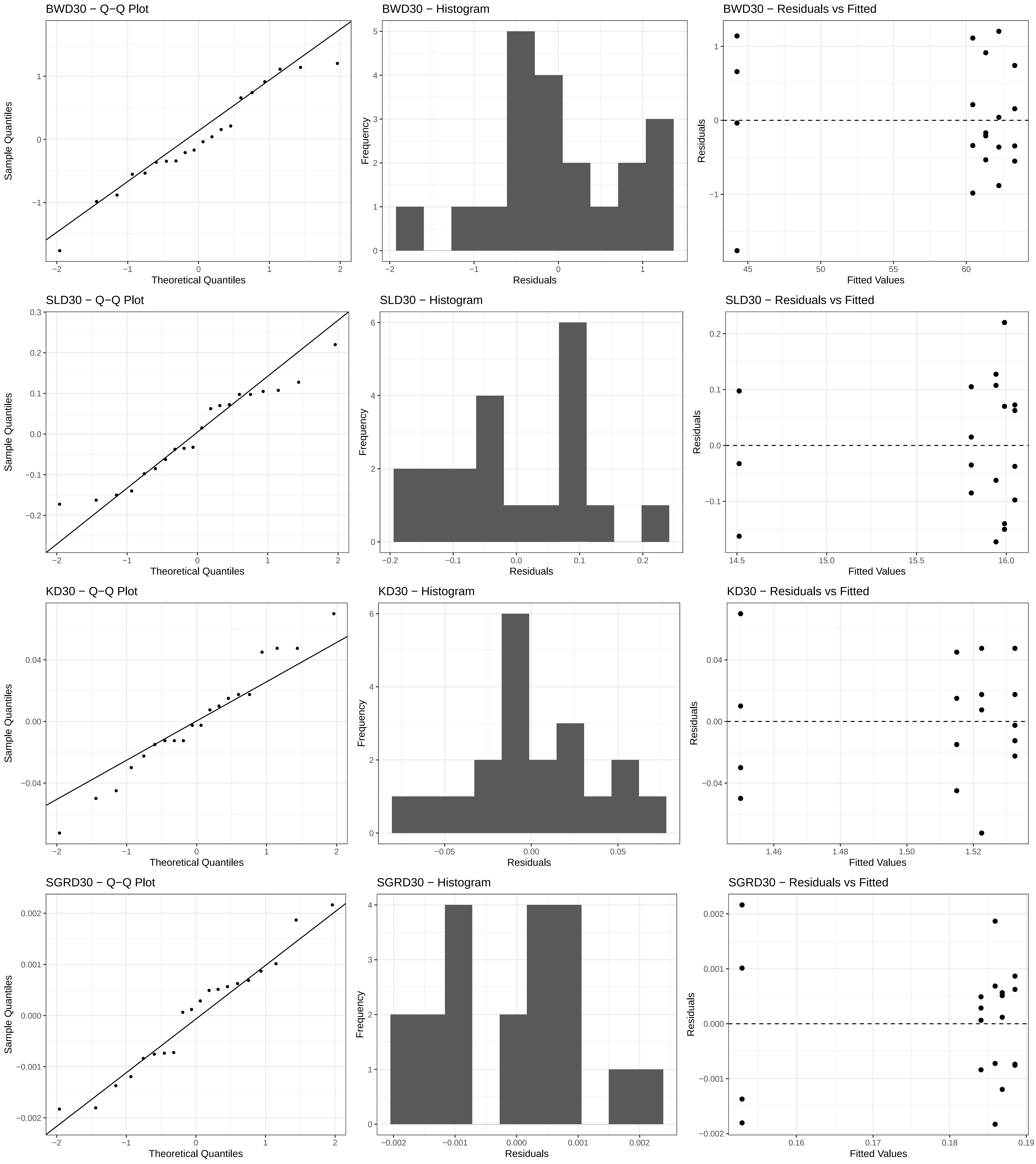

BWD30 − Q−Q Plot
Sample Quantiles
Theoretical Quantiles
BWD30 − Histogram
Frequency
Residuals
BWD30 − Residuals vs Fitted
Residuals
Fitted Values
SLD30 − Q−Q Plot
Sample Quantiles
Theoretical Quantiles
SLD30 − Histogram
Frequency
Residuals
SLD30 − Residuals vs Fitted
Residuals
Fitted Values
KD30 − Q−Q Plot
Sample Quantiles
Theoretical Quantiles
KD30 − Histogram
Frequency
Residuals
KD30 − Residuals vs Fitted
Residuals
Fitted Values
SGRD30 − Q−Q Plot
Sample Quantiles
Theoretical Quantiles
SGRD30 − Histogram
Frequency
Residuals
SGRD30 − Residuals vs Fitted
Residuals
Fitted Values

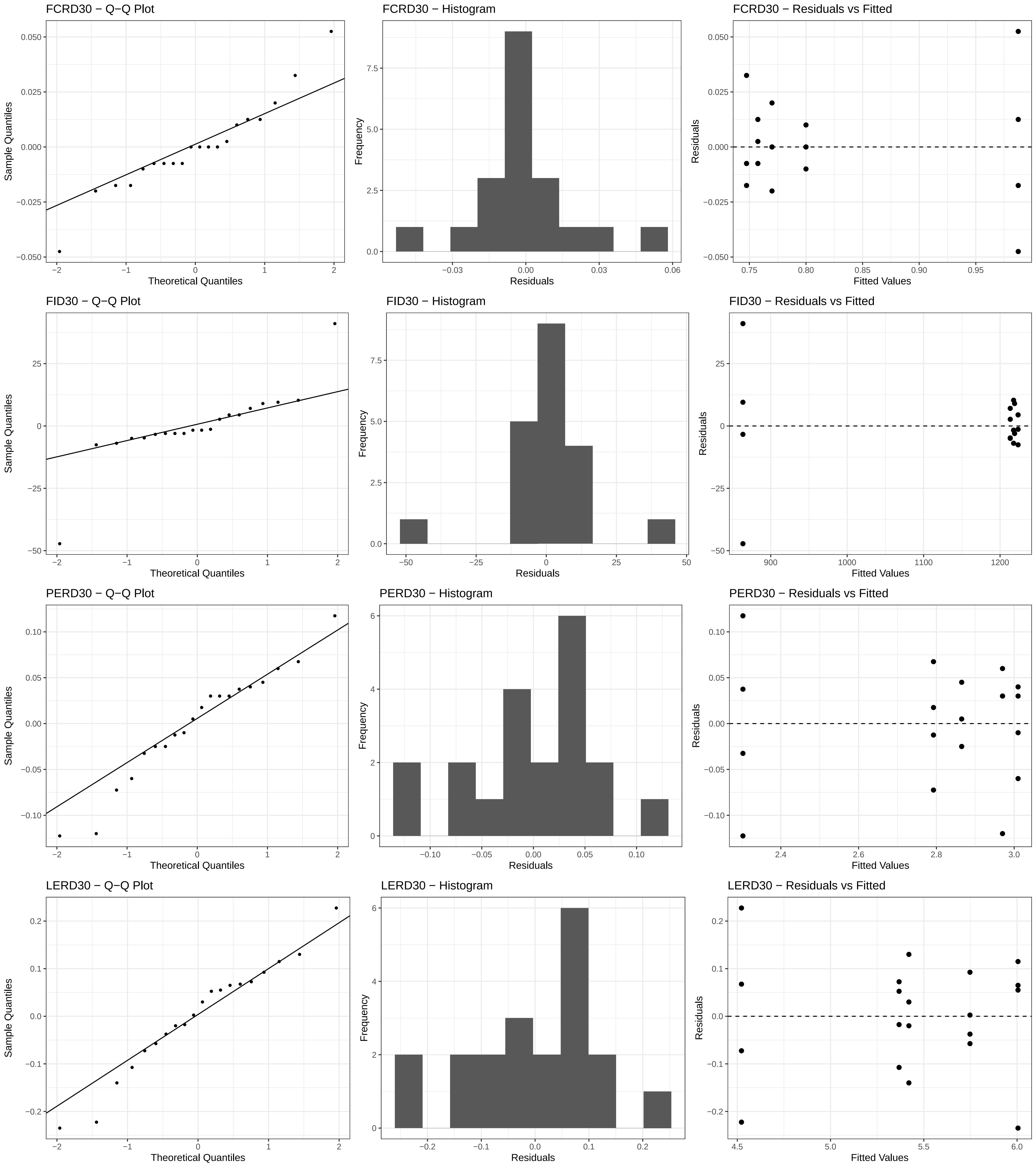
FCRD30 − Q−Q Plot
FCRD30 − Histogram
FCRD30 − Residuals vs Fitted
FID30 − Q−Q Plot
FID30 − Histogram
FID30 − Residuals vs Fitted
PERD30 − Q−Q Plot
PERD30 − Histogram
PERD30 − Residuals vs Fitted
LERD30 − Q−Q Plot
LERD30 − Histogram
LERD30 − Residuals vs Fitted
Sample Quantiles
Theoretical Quantiles
Frequency
Residuals
Fitted Values

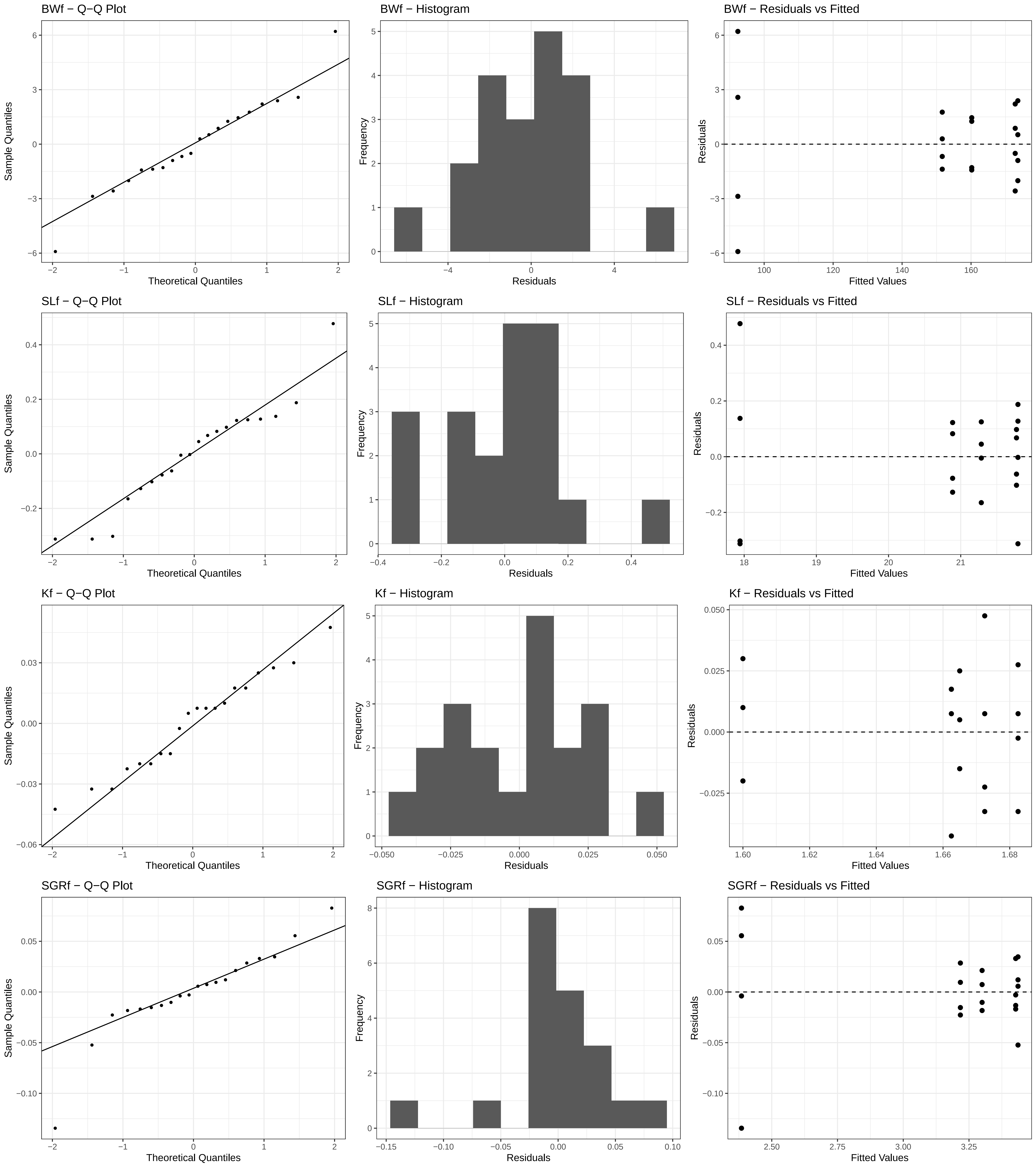

BWf − Q−Q Plot
Sample Quantiles
Theoretical Quantiles
BWf − Histogram
Frequency
Residuals
BWf − Residuals vs Fitted
Residuals
Fitted Values
SLf − Q−Q Plot
Sample Quantiles
Theoretical Quantiles
SLf − Histogram
Frequency
Residuals
SLf − Residuals vs Fitted
Residuals
Fitted Values
Kf − Q−Q Plot
Sample Quantiles
Theoretical Quantiles
Kf − Histogram
Frequency
Residuals
Kf − Residuals vs Fitted
Residuals
Fitted Values
SGRf − Q−Q Plot
Sample Quantiles
Theoretical Quantiles
SGRf − Histogram
Frequency
Residuals
SGRf − Residuals vs Fitted
Residuals
Fitted Values

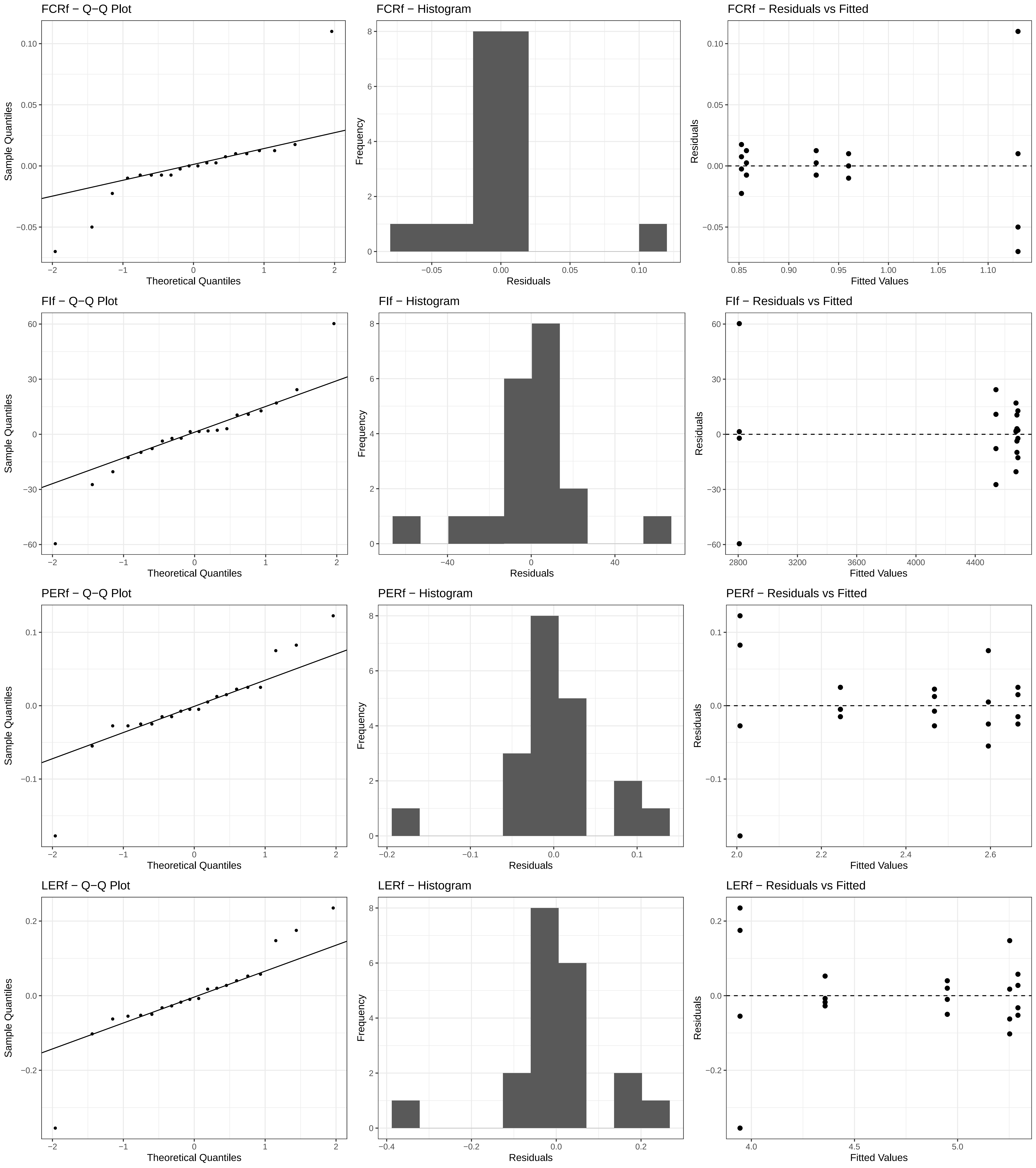
FCRf − Q−Q Plot
Sample Quantiles
Theoretical Quantiles
FCRf − Histogram
Frequency
Residuals
FCRf − Residuals vs Fitted
Residuals
Fitted Values
FIf − Q−Q Plot
Sample Quantiles
Theoretical Quantiles
FIf − Histogram
Frequency
Residuals
FIf − Residuals vs Fitted
Residuals
Fitted Values
PERf − Q−Q Plot
Sample Quantiles
Theoretical Quantiles
PERf − Histogram
Frequency
Residuals
PERf − Residuals vs Fitted
Residuals
Fitted Values
LERf − Q−Q Plot
Sample Quantiles
Theoretical Quantiles
LERf − Histogram
Frequency
Residuals
LERf − Residuals vs Fitted
Residuals
Fitted Values

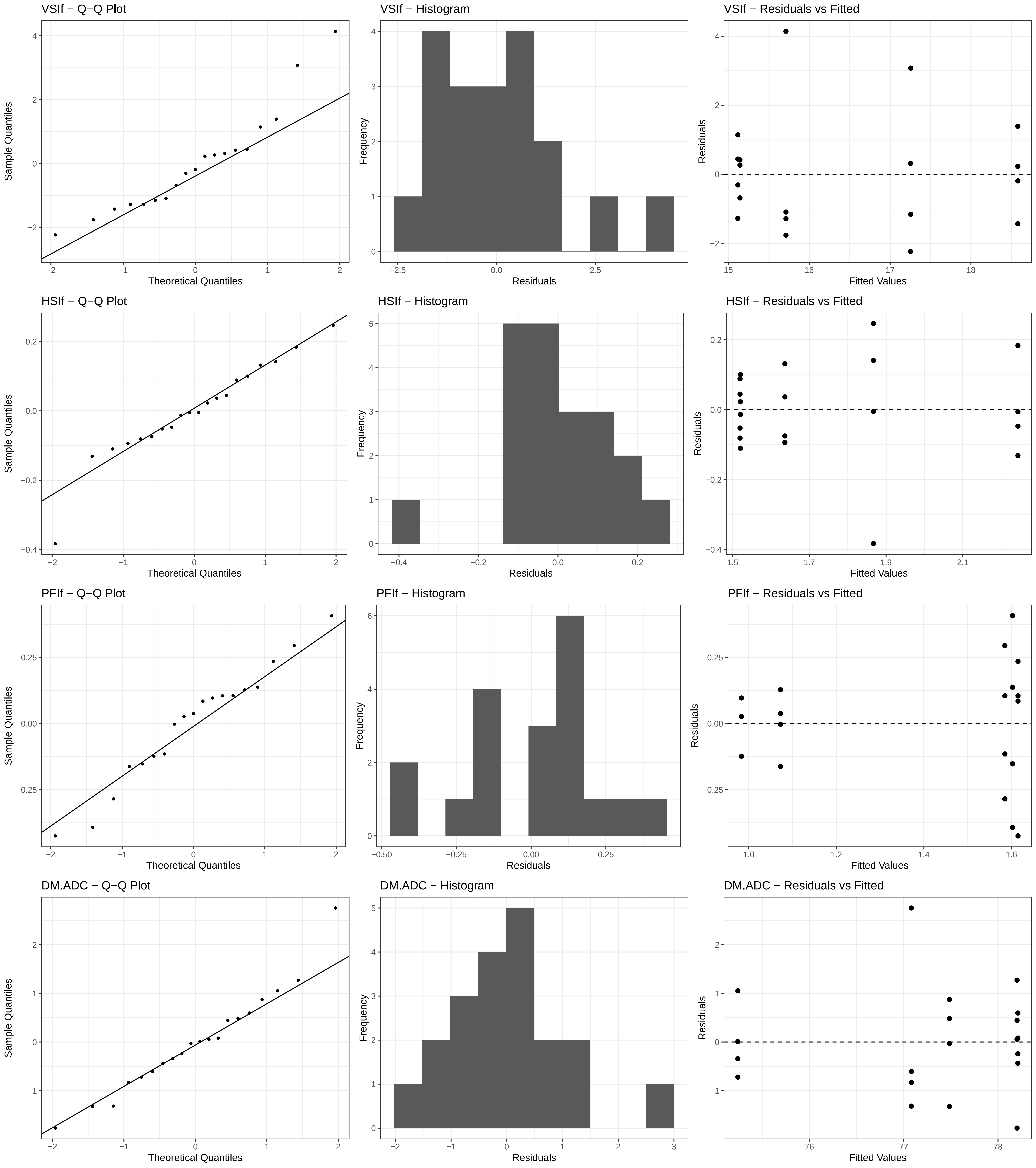
VSIf − Q−Q Plot
Sample Quantiles
Theoretical Quantiles
VSIf − Histogram
Frequency
Residuals
VSIf − Residuals vs Fitted
Residuals
Fitted Values
HSIf − Q−Q Plot
Sample Quantiles
Theoretical Quantiles
HSIf − Histogram
Frequency
Residuals
HSIf − Residuals vs Fitted
Residuals
Fitted Values
PFIf − Q−Q Plot
Sample Quantiles
Theoretical Quantiles
PFIf − Histogram
Frequency
Residuals
PFIf − Residuals vs Fitted
Residuals
Fitted Values
DM.ADC − Q−Q Plot
Sample Quantiles
Theoretical Quantiles
DM.ADC − Histogram
Frequency
Residuals
DM.ADC − Residuals vs Fitted
Residuals
Fitted Values

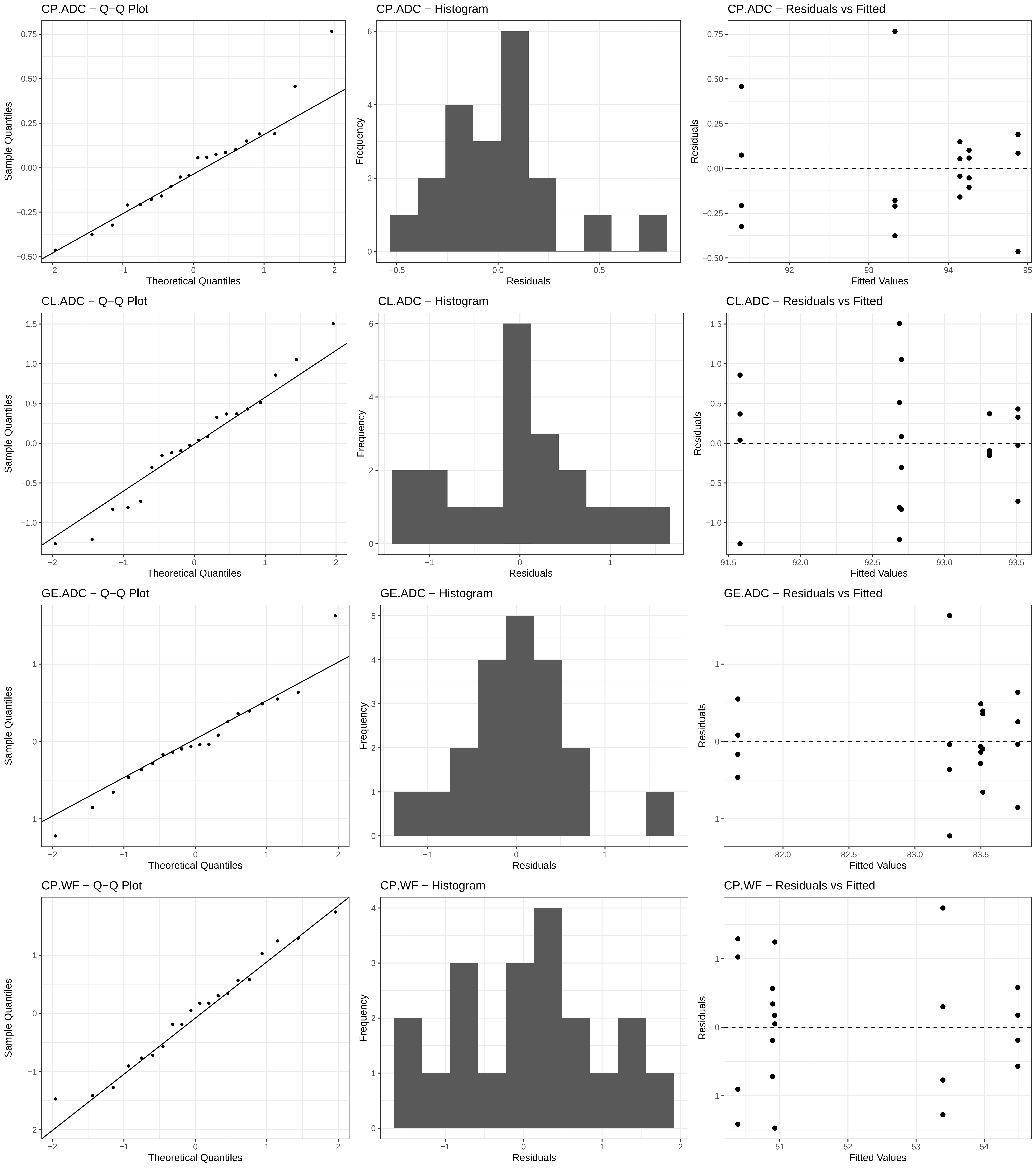
CP.ADC − Q−Q Plot
Sample Quantiles
Theoretical Quantiles
CP.ADC − Histogram
Frequency
Residuals
CP.ADC − Residuals vs Fitted
Residuals
Fitted Values
CL.ADC − Q−Q Plot
Sample Quantiles
Theoretical Quantiles
CL.ADC − Histogram
Frequency
Residuals
CL.ADC − Residuals vs Fitted
Residuals
Fitted Values
GE.ADC − Q−Q Plot
Sample Quantiles
Theoretical Quantiles
GE.ADC − Histogram
Frequency
Residuals
GE.ADC − Residuals vs Fitted
Residuals
Fitted Values
CP.WF − Q−Q Plot
Sample Quantiles
Theoretical Quantiles
CP.WF − Histogram
Frequency
Residuals
CP.WF − Residuals vs Fitted
Residuals
Fitted Values

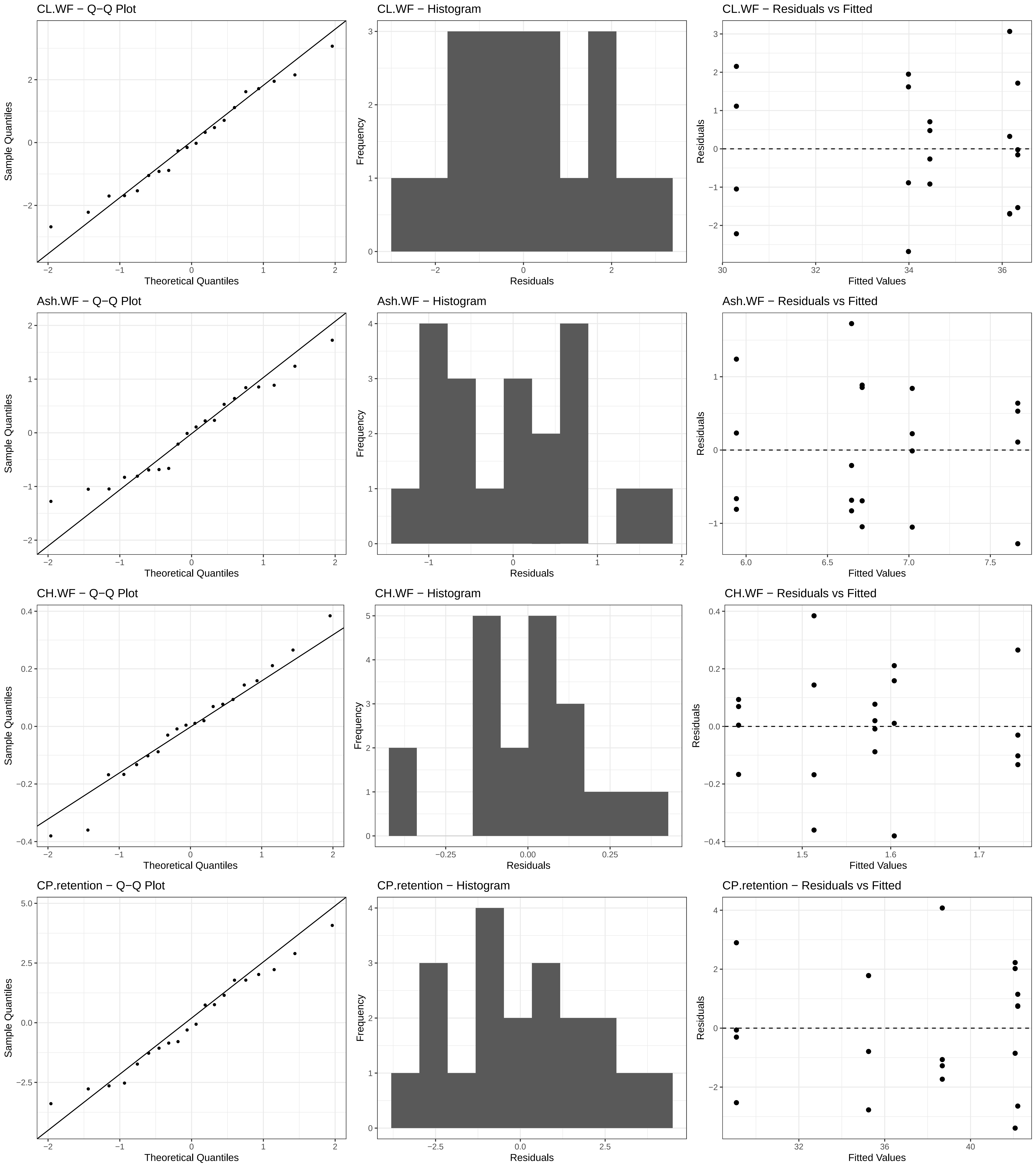
CL.WF − Q−Q Plot
Sample Quantiles
Theoretical Quantiles
CL.WF − Histogram
Frequency
Residuals
CL.WF − Residuals vs Fitted
Residuals
Fitted Values
Ash.WF − Q−Q Plot
Sample Quantiles
Theoretical Quantiles
Ash.WF − Histogram
Frequency
Residuals
Ash.WF − Residuals vs Fitted
Residuals
Fitted Values
CH.WF − Q−Q Plot
Sample Quantiles
Theoretical Quantiles
CH.WF − Histogram
Frequency
Residuals
CH.WF − Residuals vs Fitted
Residuals
Fitted Values
CP.retention − Q−Q Plot
Sample Quantiles
Theoretical Quantiles
CP.retention − Histogram
Frequency
Residuals
CP.retention − Residuals vs Fitted
Residuals
Fitted Values

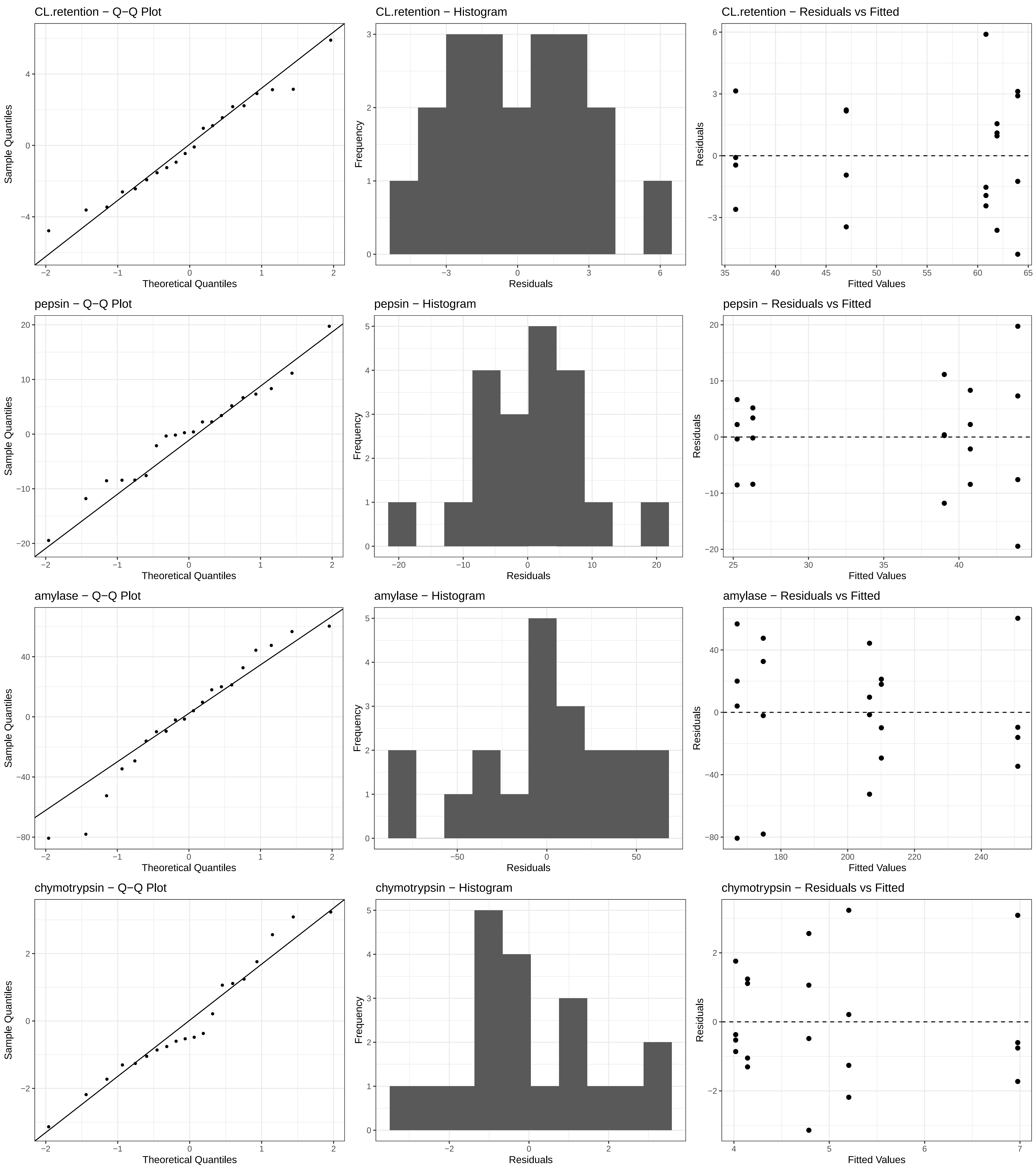
CL.retention − Q−Q Plot
Sample Quantiles
Theoretical Quantiles
CL.retention − Histogram
Frequency
Residuals
CL.retention − Residuals vs Fitted
Residuals
Fitted Values
pepsin − Q−Q Plot
Sample Quantiles
Theoretical Quantiles
pepsin − Histogram
Frequency
Residuals
pepsin − Residuals vs Fitted
Residuals
Fitted Values
amylase − Q−Q Plot
Sample Quantiles
Theoretical Quantiles
amylase − Histogram
Frequency
Residuals
amylase − Residuals vs Fitted
Residuals
Fitted Values
chymotrypsin − Q−Q Plot
Sample Quantiles
Theoretical Quantiles
chymotrypsin − Histogram
Frequency
Residuals
chymotrypsin − Residuals vs Fitted
Residuals
Fitted Values

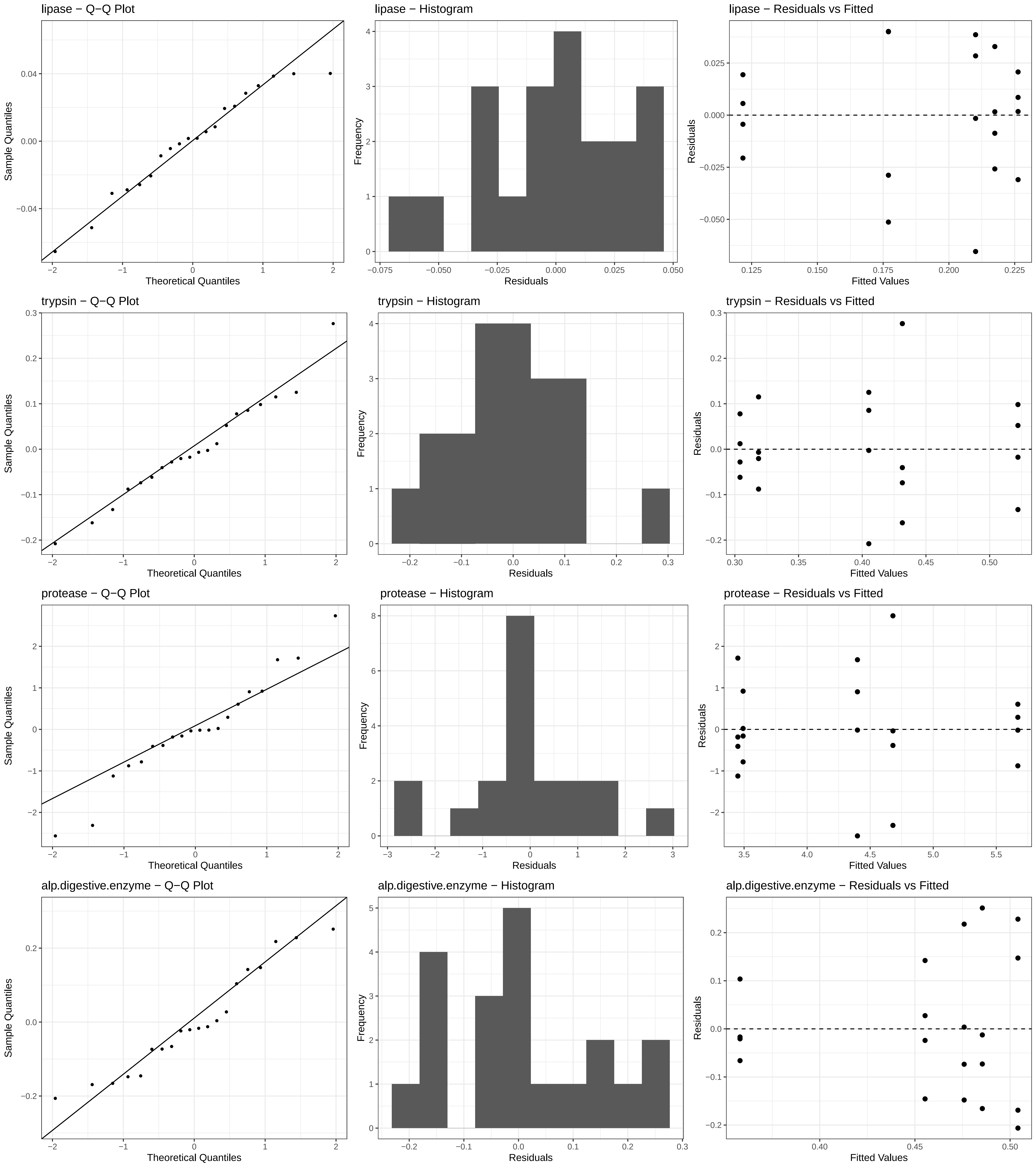
lipase − Q−Q Plot
Sample Quantiles
Theoretical Quantiles
lipase − Histogram
Frequency
Residuals
lipase − Residuals vs Fitted
Residuals
Fitted Values
trypsin − Q−Q Plot
Sample Quantiles
Theoretical Quantiles
trypsin − Histogram
Frequency
Residuals
trypsin − Residuals vs Fitted
Residuals
Fitted Values
protease − Q−Q Plot
Sample Quantiles
Theoretical Quantiles
protease − Histogram
Frequency
Residuals
protease − Residuals vs Fitted
Residuals
Fitted Values
alp.digestive.enzyme − Q−Q Plot
Sample Quantiles
Theoretical Quantiles
alp.digestive.enzyme − Histogram
Frequency
Residuals
alp.digestive.enzyme − Residuals vs Fitted
Residuals
Fitted Values

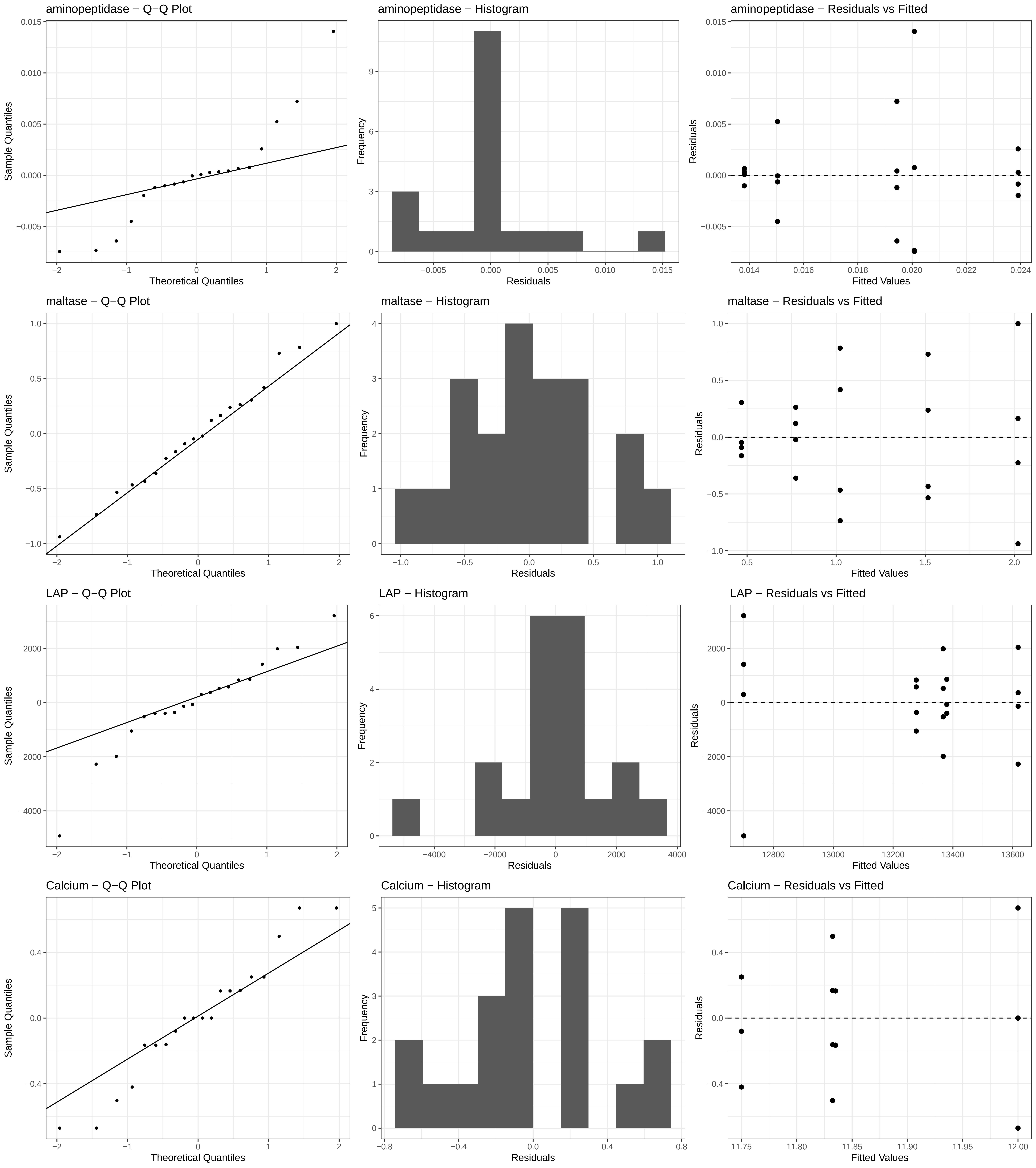
aminopeptidase − Q−Q Plot
Sample Quantiles
Theoretical Quantiles
aminopeptidase − Histogram
Frequency
Residuals
aminopeptidase − Residuals vs Fitted
Residuals
Fitted Values
maltase − Q−Q Plot
Sample Quantiles
Theoretical Quantiles
maltase − Histogram
Frequency
Residuals
maltase − Residuals vs Fitted
Residuals
Fitted Values
LAP − Q−Q Plot
Sample Quantiles
Theoretical Quantiles
LAP − Histogram
Frequency
Residuals
LAP − Residuals vs Fitted
Residuals
Fitted Values
Calcium − Q−Q Plot
Sample Quantiles
Theoretical Quantiles
Calcium − Histogram
Frequency
Residuals
Calcium − Residuals vs Fitted
Residuals
Fitted Values

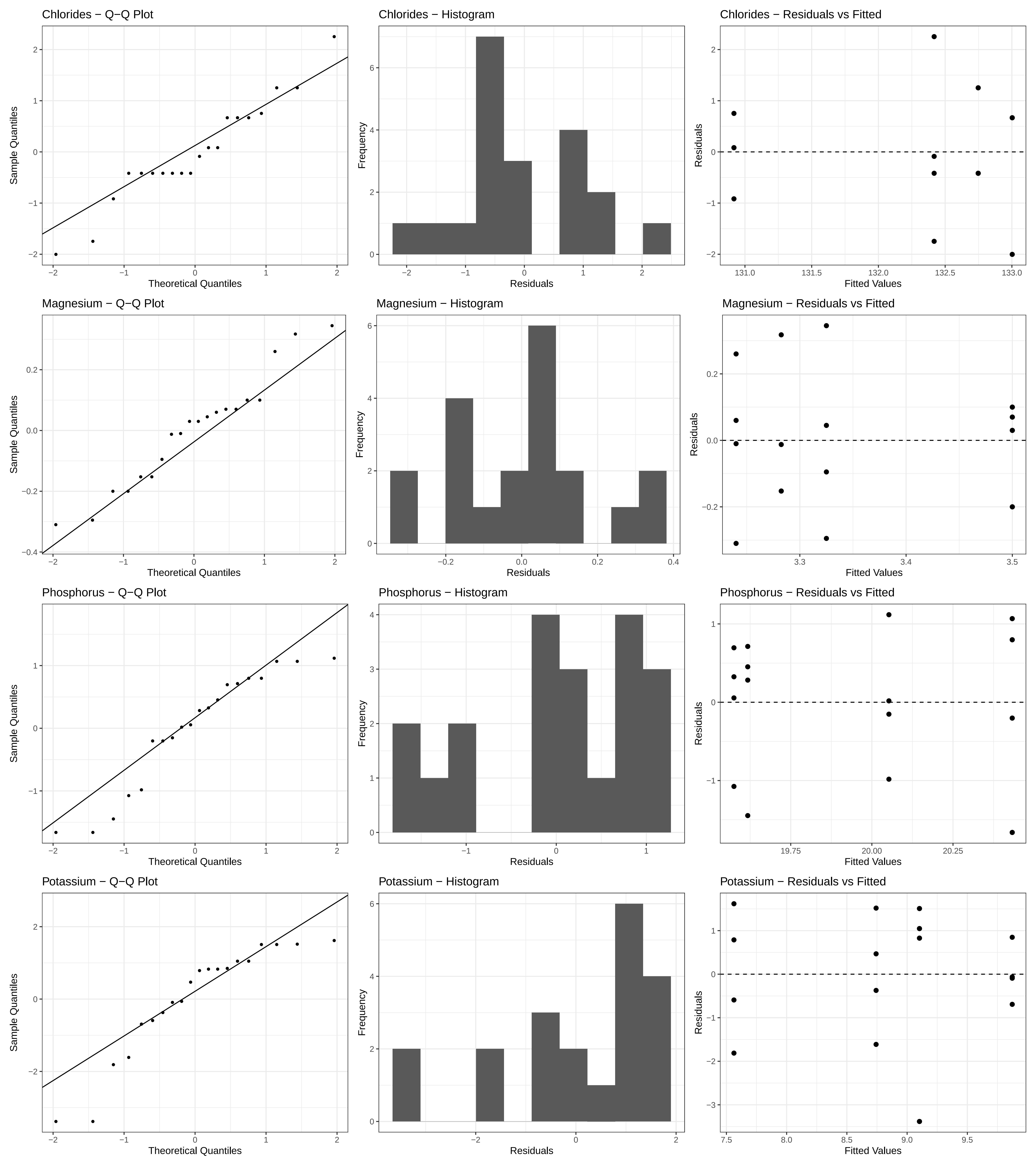
Chlorides − Q−Q Plot
Sample Quantiles
Theoretical Quantiles
Chlorides − Histogram
Frequency
Residuals
Chlorides − Residuals vs Fitted
Residuals
Fitted Values
Magnesium − Q−Q Plot
Sample Quantiles
Theoretical Quantiles
Magnesium − Histogram
Frequency
Residuals
Magnesium − Residuals vs Fitted
Residuals
Fitted Values
Phosphorus − Q−Q Plot
Sample Quantiles
Theoretical Quantiles
Phosphorus − Histogram
Frequency
Residuals
Phosphorus − Residuals vs Fitted
Residuals
Fitted Values
Potassium − Q−Q Plot
Sample Quantiles
Theoretical Quantiles
Potassium − Histogram
Frequency
Residuals
Potassium − Residuals vs Fitted
Residuals
Fitted Values

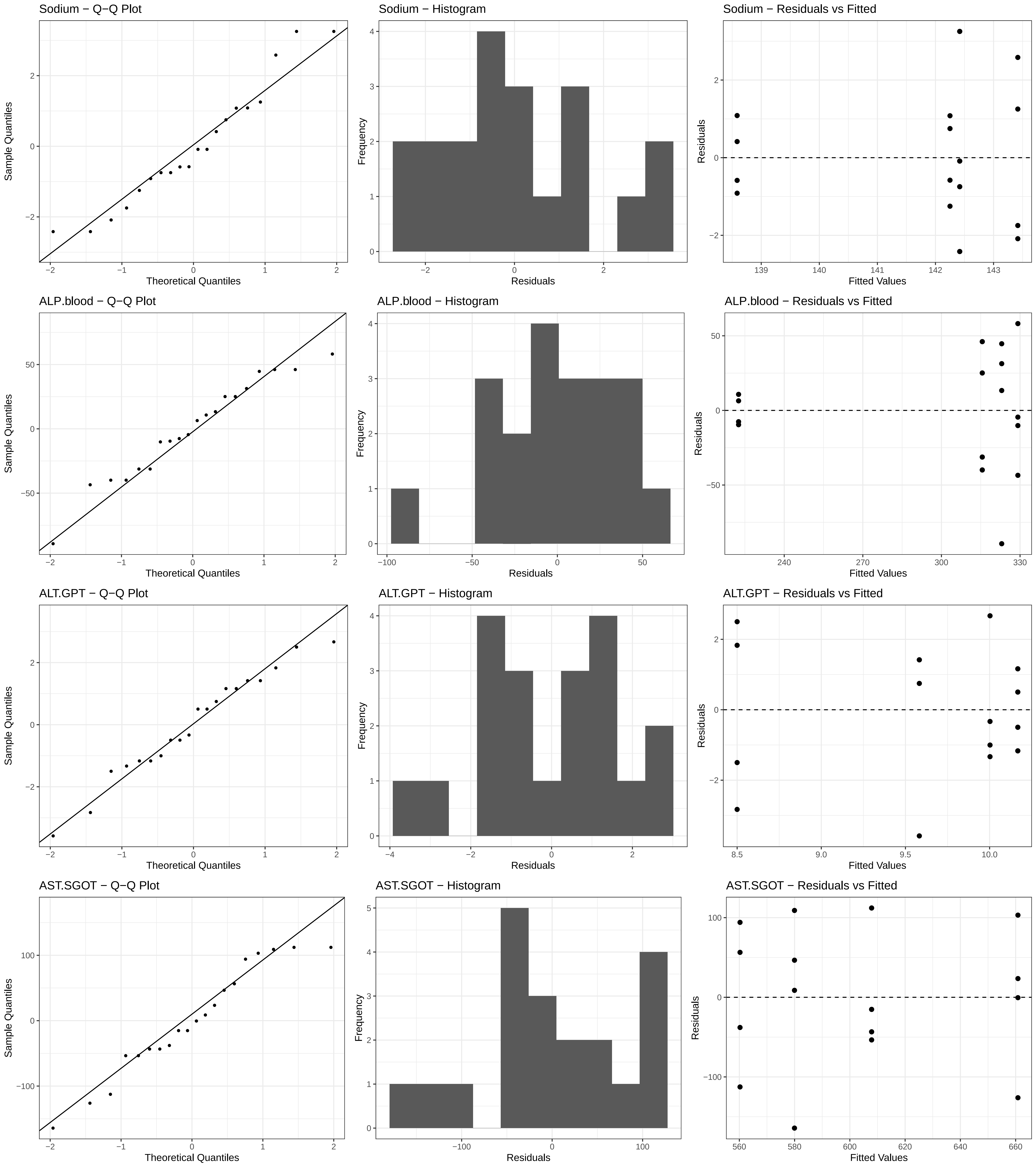
Sodium − Q−Q Plot
Sample Quantiles
Theoretical Quantiles
−2 −1 0 1 2
Sodium − Histogram
Frequency
Residuals
0 1 2 3 4
Sodium − Residuals vs Fitted
Residuals
Fitted Values
139 140 141 142 143
ALP.blood − Q−Q Plot
Sample Quantiles
Theoretical Quantiles
−50 0 50
ALP.blood − Histogram
Frequency
Residuals
−100 −50 0 50
ALP.blood − Residuals vs Fitted
Residuals
Fitted Values
240 270 300 330
ALT.GPT − Q−Q Plot
Sample Quantiles
Theoretical Quantiles
ALT.GPT − Histogram
Frequency
Residuals
−4 −2 0 2
ALT.GPT − Residuals vs Fitted
Residuals
Fitted Values
8.5 9.0 9.5 10.0
AST.SGOT − Q−Q Plot
Sample Quantiles
Theoretical Quantiles
−100 0 100
AST.SGOT − Histogram
Frequency
Residuals
0 1 2 3 4 5
AST.SGOT − Residuals vs Fitted
Residuals
Fitted Values
560 580 600 620 640 660

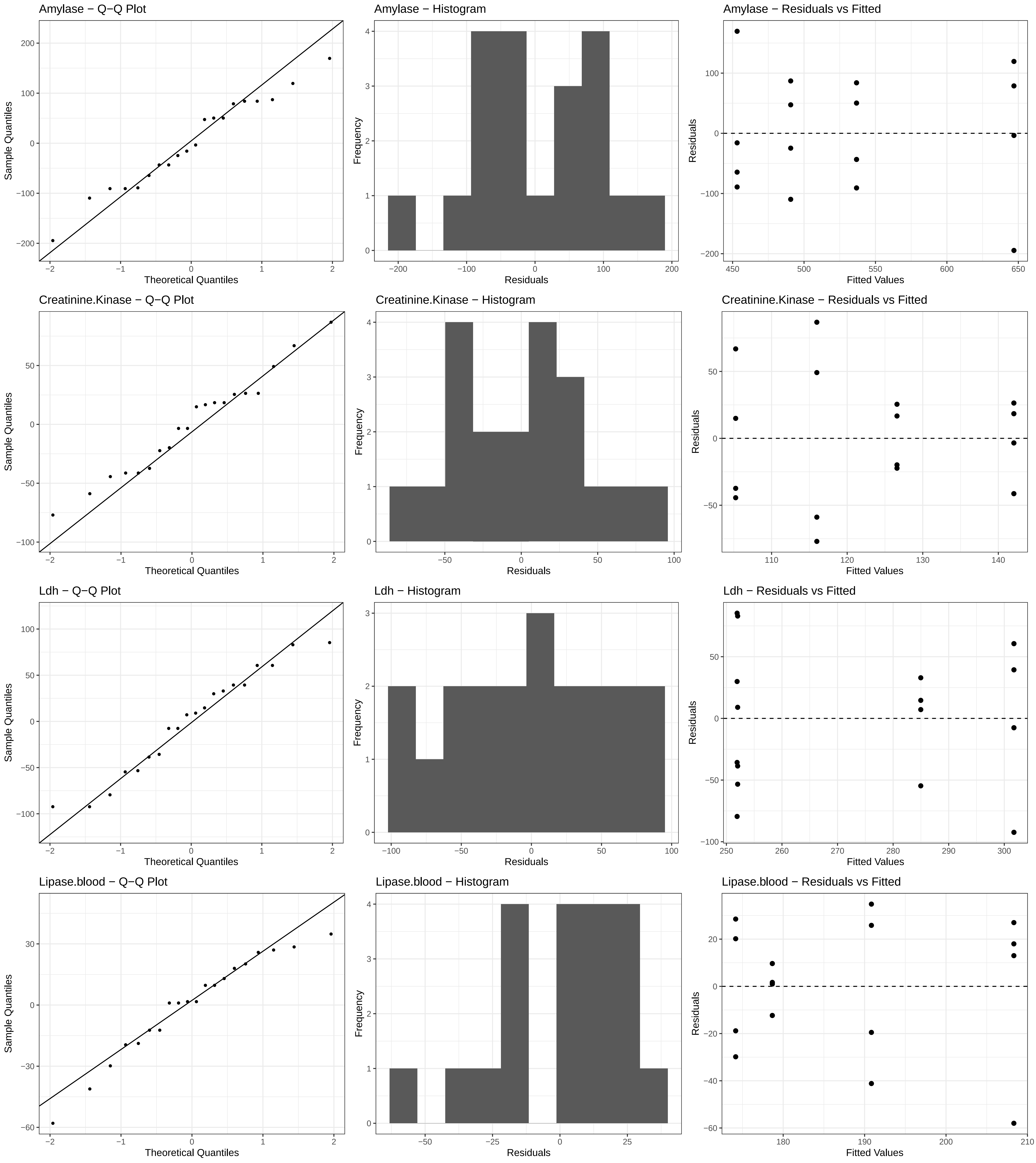

Amylase − Q−Q Plot
Sample Quantiles
Theoretical Quantiles
Amylase − Histogram
Frequency
Residuals
Amylase − Residuals vs Fitted
Residuals
Fitted Values
Creatinine.Kinase − Q−Q Plot
Sample Quantiles
Theoretical Quantiles
Creatinine.Kinase − Histogram
Frequency
Residuals
Creatinine.Kinase − Residuals vs Fitted
Residuals
Fitted Values
Ldh − Q−Q Plot
Sample Quantiles
Theoretical Quantiles
Ldh − Histogram
Frequency
Residuals
Ldh − Residuals vs Fitted
Residuals
Fitted Values
Lipase.blood − Q−Q Plot
Sample Quantiles
Theoretical Quantiles
Lipase.blood − Histogram
Frequency
Residuals
Lipase.blood − Residuals vs Fitted
Residuals
Fitted Values

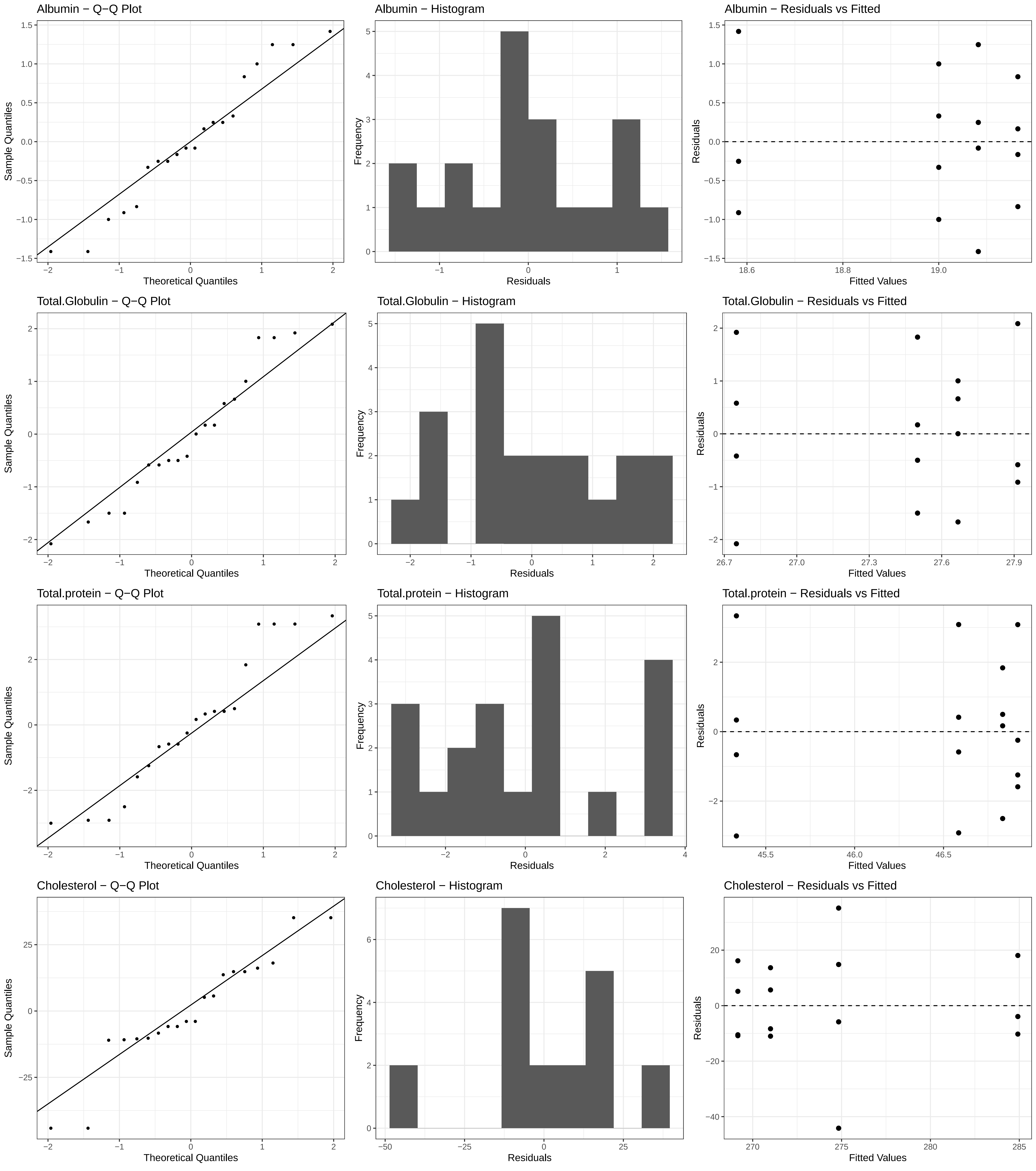

Albumin − Q−Q Plot
Sample Quantiles
Theoretical Quantiles
Albumin − Histogram
Frequency
Residuals
Albumin − Residuals vs Fitted
Residuals
Fitted Values
Total.Globulin − Q−Q Plot
Sample Quantiles
Theoretical Quantiles
Total.Globulin − Histogram
Frequency
Residuals
Total.Globulin − Residuals vs Fitted
Residuals
Fitted Values
Total.protein − Q−Q Plot
Sample Quantiles
Theoretical Quantiles
Total.protein − Histogram
Frequency
Residuals
Total.protein − Residuals vs Fitted
Residuals
Fitted Values
Cholesterol − Q−Q Plot
Sample Quantiles
Theoretical Quantiles
Cholesterol − Histogram
Frequency
Residuals
Cholesterol − Residuals vs Fitted
Residuals
Fitted Values

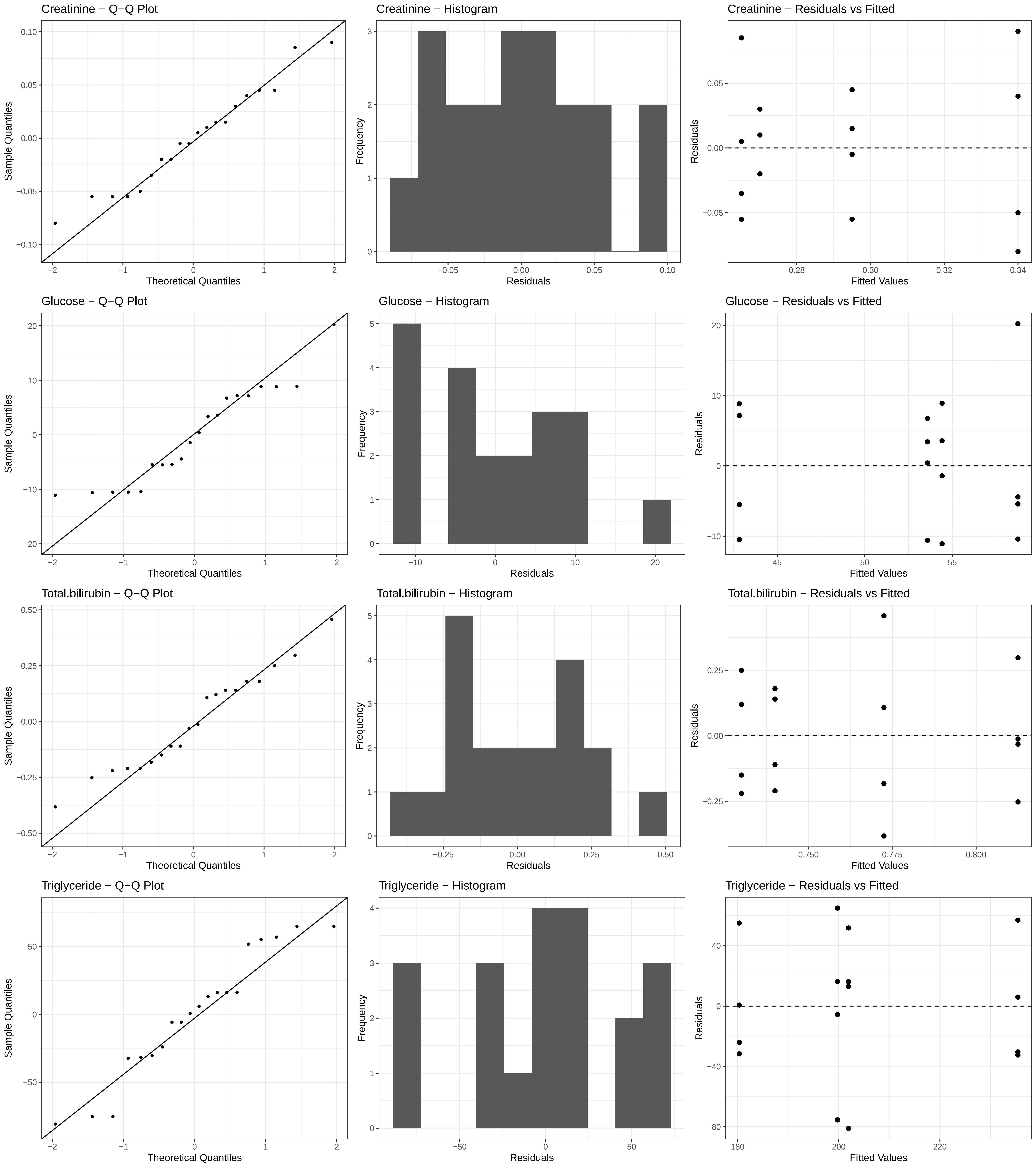

Creatinine − Q−Q Plot
Sample Quantiles
Theoretical Quantiles
Creatinine − Histogram
Frequency
Residuals
Creatinine − Residuals vs Fitted
Residuals
Fitted Values
Glucose − Q−Q Plot
Sample Quantiles
Theoretical Quantiles
Glucose − Histogram
Frequency
Residuals
Glucose − Residuals vs Fitted
Residuals
Fitted Values
Total.bilirubin − Q−Q Plot
Sample Quantiles
Theoretical Quantiles
Total.bilirubin − Histogram
Frequency
Residuals
Total.bilirubin − Residuals vs Fitted
Residuals
Fitted Values
Triglyceride − Q−Q Plot
Sample Quantiles
Theoretical Quantiles
Triglyceride − Histogram
Frequency
Residuals
Triglyceride − Residuals vs Fitted
Residuals
Fitted Values

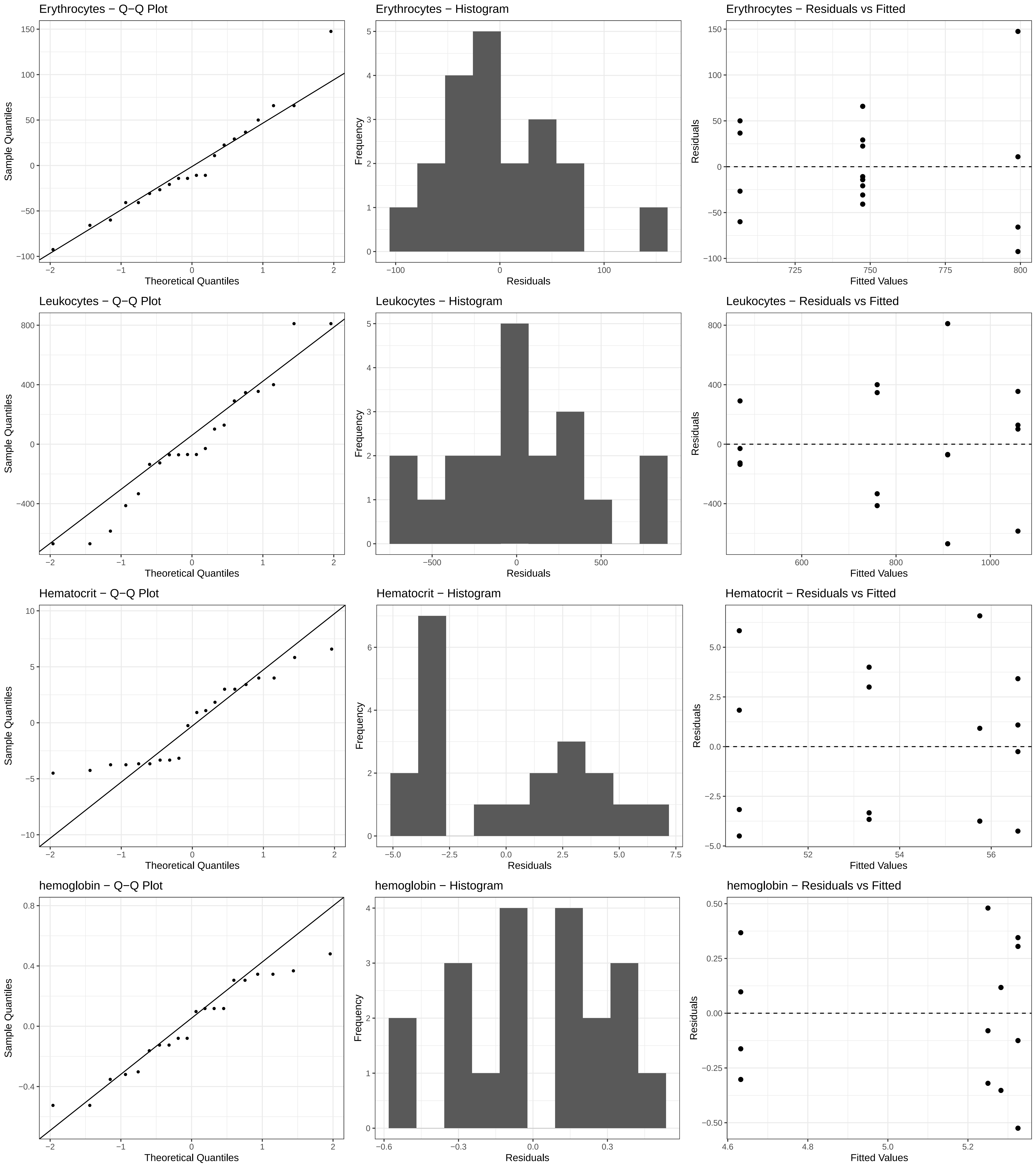

Erythrocytes − Q−Q Plot
Sample Quantiles
Theoretical Quantiles
Erythrocytes − Histogram
Frequency
Residuals
Erythrocytes − Residuals vs Fitted
Residuals
Fitted Values
Leukocytes − Q−Q Plot
Sample Quantiles
Theoretical Quantiles
Leukocytes − Histogram
Frequency
Residuals
Leukocytes − Residuals vs Fitted
Residuals
Fitted Values
Hematocrit − Q−Q Plot
Sample Quantiles
Theoretical Quantiles
Hematocrit − Histogram
Frequency
Residuals
Hematocrit − Residuals vs Fitted
Residuals
Fitted Values
hemoglobin − Q−Q Plot
Sample Quantiles
Theoretical Quantiles
hemoglobin − Histogram
Frequency
Residuals
hemoglobin − Residuals vs Fitted
Residuals
Fitted Values

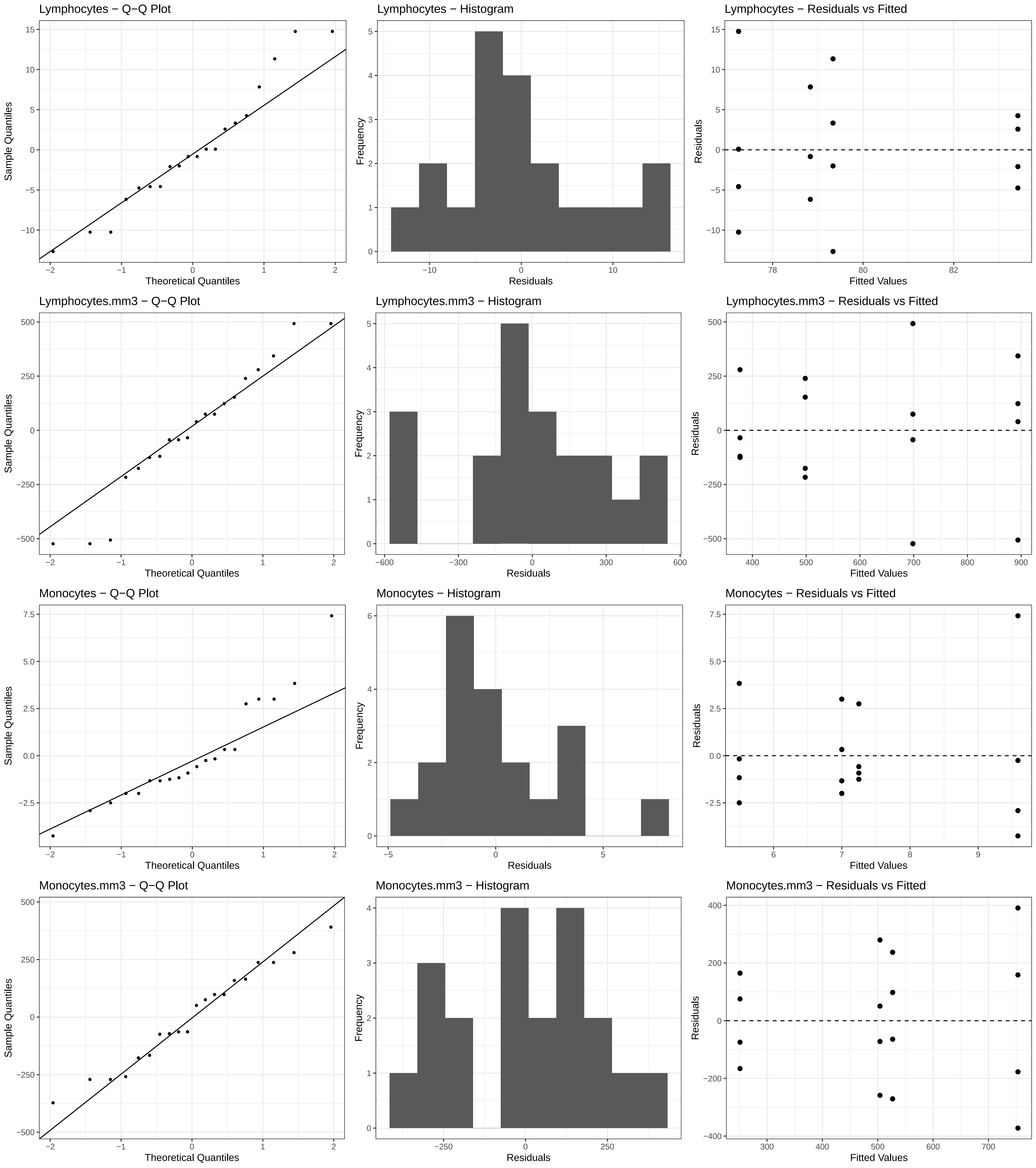

Lymphocytes − Q−Q Plot
Sample Quantiles
Theoretical Quantiles
Lymphocytes − Histogram
Frequency
Residuals
Lymphocytes − Residuals vs Fitted
Residuals
Fitted Values
Lymphocytes.mm3 − Q−Q Plot
Sample Quantiles
Theoretical Quantiles
Lymphocytes.mm3 − Histogram
Frequency
Residuals
Lymphocytes.mm3 − Residuals vs Fitted
Residuals
Fitted Values
Monocytes − Q−Q Plot
Sample Quantiles
Theoretical Quantiles
Monocytes − Histogram
Frequency
Residuals
Monocytes − Residuals vs Fitted
Residuals
Fitted Values
Monocytes.mm3 − Q−Q Plot
Sample Quantiles
Theoretical Quantiles
Monocytes.mm3 − Histogram
Frequency
Residuals
Monocytes.mm3 − Residuals vs Fitted
Residuals
Fitted Values

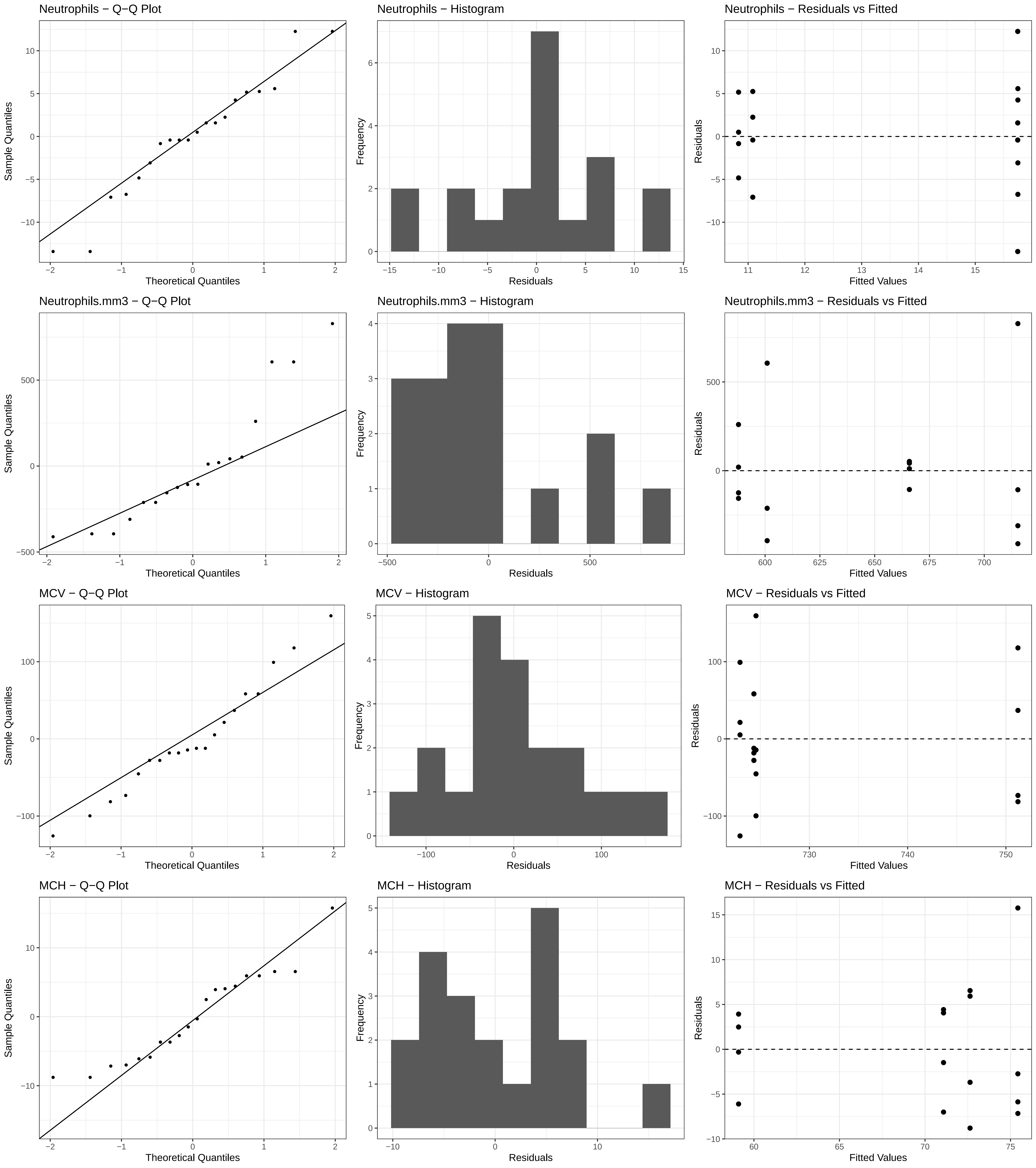
Neutrophils − Q−Q Plot
Sample Quantiles
Theoretical Quantiles
Neutrophils − Histogram
Frequency
Residuals
Neutrophils − Residuals vs Fitted
Residuals
Fitted Values
Neutrophils.mm3 − Q−Q Plot
Sample Quantiles
Theoretical Quantiles
Neutrophils.mm3 − Histogram
Frequency
Residuals
Neutrophils.mm3 − Residuals vs Fitted
Residuals
Fitted Values
MCV − Q−Q Plot
Sample Quantiles
Theoretical Quantiles
MCV − Histogram
Frequency
Residuals
MCV − Residuals vs Fitted
Residuals
Fitted Values
MCH − Q−Q Plot
Sample Quantiles
Theoretical Quantiles
MCH − Histogram
Frequency
Residuals
MCH − Residuals vs Fitted
Residuals
Fitted Values

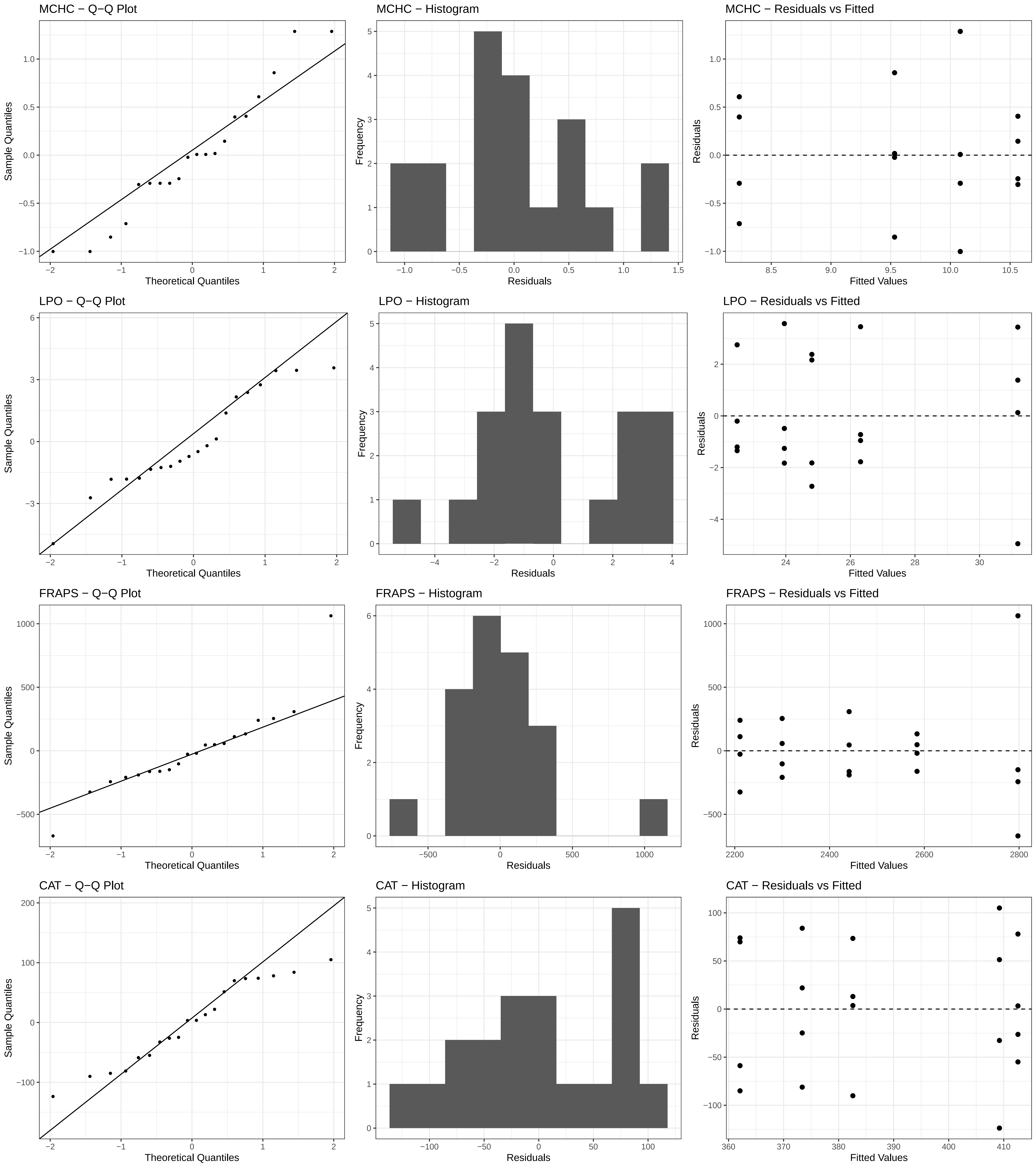

MCHC − Q−Q Plot
Sample Quantiles
Theoretical Quantiles
MCHC − Histogram
Frequency
Residuals
MCHC − Residuals vs Fitted
Residuals
Fitted Values
LPO − Q−Q Plot
Sample Quantiles
Theoretical Quantiles
LPO − Histogram
Frequency
Residuals
LPO − Residuals vs Fitted
Residuals
Fitted Values
FRAPS − Q−Q Plot
Sample Quantiles
Theoretical Quantiles
FRAPS − Histogram
Frequency
Residuals
FRAPS − Residuals vs Fitted
Residuals
Fitted Values
CAT − Q−Q Plot
Sample Quantiles
Theoretical Quantiles
CAT − Histogram
Frequency
Residuals
CAT − Residuals vs Fitted
Residuals
Fitted Values

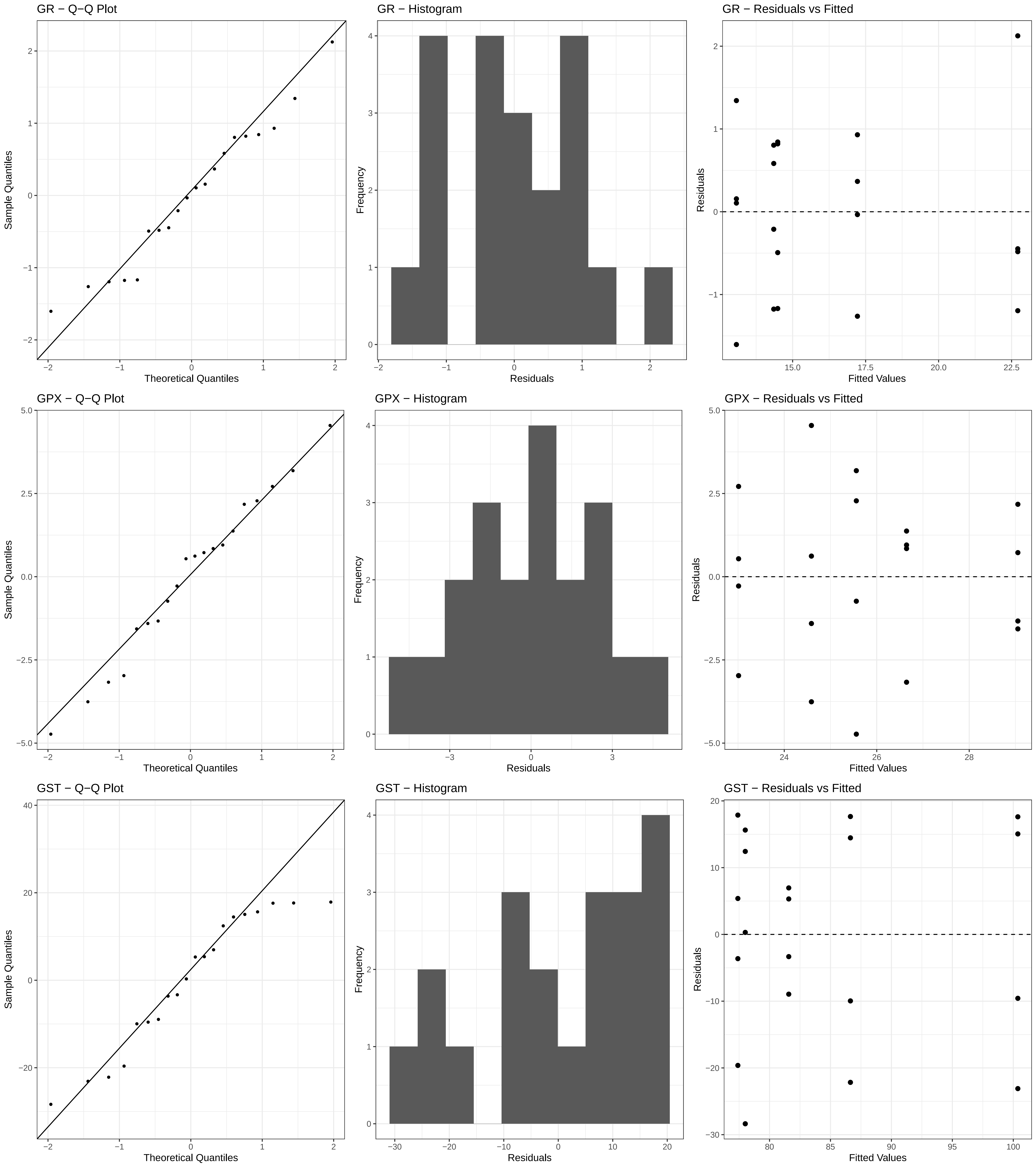
GR − Q−Q Plot
Sample Quantiles
Theoretical Quantiles
GR − Histogram
Frequency
Residuals
GR − Residuals vs Fitted
Residuals
Fitted Values
GPX − Q−Q Plot
Sample Quantiles
Theoretical Quantiles
GPX − Histogram
Frequency
Residuals
GPX − Residuals vs Fitted
Residuals
Fitted Values
GST − Q−Q Plot
Sample Quantiles
Theoretical Quantiles
GST − Histogram
Frequency
Residuals
GST − Residuals vs Fitted
Residuals
Fitted Values
