# Supplementary material for: Valorization of Mushroom By‐Products From Agaricus bisporus and Pleurotus ostreatus as Novel Feed Ingredients for Rainbow Trout (Oncorhynchus mykiss) Diets
Source: Aquac Nutr. 2026 Aug 3;2026:6294426. doi: 10.1155/anu/6294426 (PMC13434034; doi:10.1155/anu/6294426)
Supplement: Supplementary file 2 [file ANU-2026-6294426-s002.pdf]

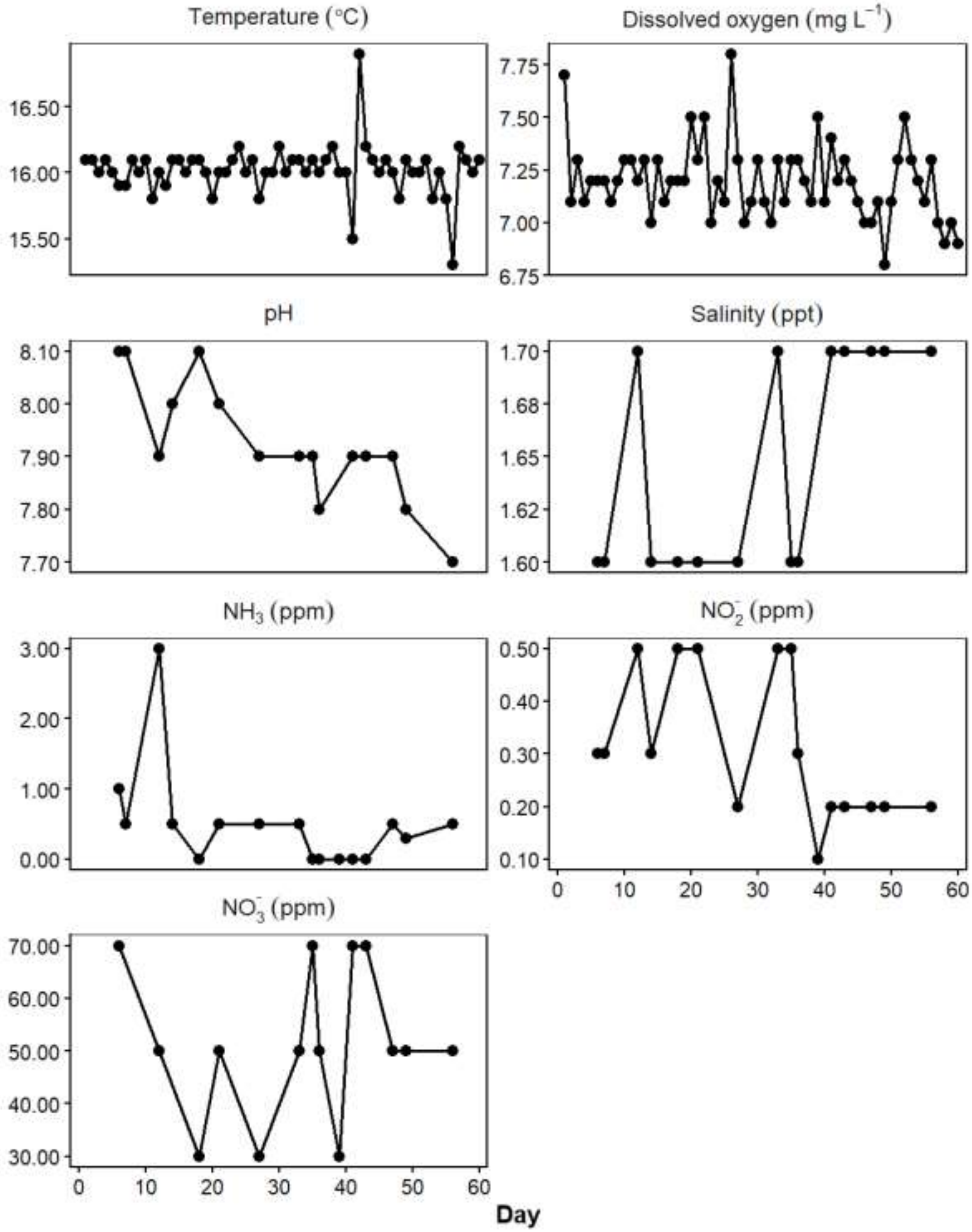

Supplementary figure 1. Temporal variation of water quality parameters monitored throughout the feeding trial of rainbow trout (*Oncorhynchus mykiss*) fed experimental diets in which soybean meal was replaced with graded levels of *Agaricus bisporus* (AB) and *Pleurotus ostreatus* (PO) meals over a 60-day period. Values correspond to routine measurements of temperature (°C), dissolved oxygen (mg L^{-1}), pH, salinity (ppt), ammonia (NH_3, ppm), nitrite (NO_2^-, ppm), and nitrate (NO_3^-, ppm). All parameters remained within the operational ranges established for rainbow trout culture throughout the experimental period.
